# Measuring progress towards reaching zero new HIV acquisitions among key populations in Québec (Canada) using routine surveillance data: a mathematical modelling study

**DOI:** 10.1002/jia2.25994

**Published:** 2022-09-01

**Authors:** Carla M. Doyle, Joseph Cox, Rachael M. Milwid, Raphaël Bitera, Charlotte Lanièce Delaunay, Michel Alary, Gilles Lambert, Cécile Tremblay, Sharmistha Mishra, Mathieu Maheu‐Giroux

**Affiliations:** ^1^ Department of Epidemiology and Biostatistics, School of Population and Global Health McGill University Montréal Québec Canada; ^2^ Direction Régionale de Santé Publique de Montréal Montréal Québec Canada; ^3^ Clinical Outcomes Research and Evaluation Research Institute ‐ McGill University Health Centre Montréal Québec Canada; ^4^ Institut national de santé publique du Québec Québec Québec Canada; ^5^ Centre de recherche du CHU de Québec – Université Laval Québec Québec Canada; ^6^ Département de médecine sociale et préventive Université Laval Québec Québec Canada; ^7^ Centre de Recherche du Centre Hospitalier de l'Université de Montréal (CRCHUM) Montréal Québec Canada; ^8^ Department of Microbiology, Infectiology and Immunology University of Montréal Montréal Québec Canada; ^9^ Department of Medicine St. Michael's Hospital University of Toronto Toronto Ontario Canada; ^10^ Institute of Medical Sciences, University of Toronto Toronto Ontario Canada; ^11^ Institute of Health Policy Management and Evaluation, Dalla Lana School of Public Health University of Toronto Toronto Ontario Canada

**Keywords:** drug use, epidemics, epidemiologic measurements, epidemiological models, HIV infections, sexual and gender minorities

## Abstract

**Introduction:**

Men who have sex with men (MSM) and people who inject drugs (PWID) are disproportionately impacted by the HIV epidemic in Canada. Having the second‐highest provincial diagnosis rate, an improved understanding of the epidemic among these populations in Québec could aid ongoing elimination efforts. We estimated HIV incidence and other epidemic indicators among MSM and PWID in Montréal and across Québec using a back‐calculation model synthesizing surveillance data.

**Methods:**

We developed a deterministic, compartmental mathematical model stratified by age, HIV status and disease progression, and clinical care stages. Using AIDS and HIV diagnoses data, including self‐reported time since the last negative test and laboratory results of CD4 cell count at diagnosis, we estimated HIV incidence in each population over 1975–2020 by modelling a cubic M‐spline. The prevalence, undiagnosed fraction, fraction diagnosed that started antiretroviral treatment (ART) and median time to diagnosis were also estimated. Since the COVID‐19 pandemic disrupted testing, we excluded 2020 data and explored this in sensitivity analyses.

**Results:**

HIV incidence in all populations peaked early in the epidemic. In 2020, an estimated 97 (95% CrI: 33–227) and 266 (95% CrI: 103–508) HIV acquisitions occurred among MSM in Montréal and Québec, respectively. Among PWID, we estimated 2 (95% CrI: 0–14) and 6 (95% CrI: 1–26) HIV acquisitions in those same regions. With 2020 data, unless testing rates were reduced by 50%, these estimates decreased, except among Québec PWID, whose increased. Among all, the median time to diagnosis shortened to <2 years before 2020 and the undiagnosed fraction decreased to <10%. This fraction was higher in younger MSM, with 22% of 15–24 year‐olds living with HIV in Montréal (95% CrI: 9–39%) and 31% in Québec (95% CrI: 17–48%) undiagnosed by 2020 year‐end. Finally, ART access neared 100% in all diagnosed populations.

**Conclusions:**

HIV incidence has drastically decreased in MSM and PWID across Québec, alongside significant improvements in diagnosis and treatment coverage—and the 2013 introduction of pre‐exposure prophylaxis. Despite this, HIV transmission continued. Effective efforts to halt this transmission and rapidly diagnose people who acquired HIV, especially among younger MSM, are needed to achieve elimination. Further, as the impacts of the COVID‐19 pandemic on HIV transmission are understood, increased efforts may be needed to overcome these.

## 1 INTRODUCTION

We have the biomedical tools to eliminate HIV [1]. Yet, transmission continues to occur in Canada. In fact, HIV diagnoses have recently stabilized at counts near those observed in the late 1990s [2,3]. Across Canada, key populations, including men who have sex with men (MSM) and people who inject drugs (PWID), are disproportionately burdened by the HIV epidemic. In Québec, which had the second‐highest provincial HIV diagnosis rate in 2019 [2], only 3.4% of adult men reported sex with another man in the previous 12 months [4]. Yet, MSM accounted for 71% of new male diagnoses that year [5]. People who ever injected drugs comprised 0.8% of Québec adults [4], but 0.7–5.5% of new diagnoses in recent years belonged to that population [5].

In 2016, Canada endorsed the *Joint United Nations Programme on HIV/AIDS* (UNAIDS) efforts to end the HIV/AIDS epidemic as a public health threat [6]. Cities, where key populations often reside, play a crucial role in HIV epidemics [7]. Québec's epidemic epicentre lies in Montréal where 61% of new 2019 diagnoses occurred [5]. In 2017, Montréal became the first UNAIDS Fast‐Track City in Canada, committing to HIV elimination by 2030 [8,9]. Interim, 2020 targets aimed for zero new HIV acquisitions, a strengthened treatment and care cascade (reaching 95% diagnosis coverage, 95% of those diagnosed on treatment, and, of those, 95% virally suppressed by 2025), zero discrimination and zero stigma [10]. Measuring the target of zero new HIV acquisitions is difficult as it cannot be directly observed. New diagnoses have been used as a proxy [10]; however, these reflect mostly past incidence and are affected by testing efforts. Analyses discerning new acquisitions from diagnoses while accounting for testing trends are needed.

Timely estimates of new HIV acquisitions are necessary to monitor progress towards elimination and identify unmet prevention needs. Doing so requires modelling tools synthesizing surveillance data, allowing the back‐calculation of HIV incidence using information, such as CD4 cell count at diagnosis and HIV testing history [11]. The *Public Health Agency of Canada* (PHAC) estimates HIV incidence using a statistical back‐calculation method based on HIV and AIDS diagnoses and HIV/AIDS‐related deaths [12]. Overall national and provincial estimates, and those stratified by exposure category (e.g. MSM and PWID), are typically produced and provided to provincial public health authorities. Overall Québec estimates have been reported up to 2018 [13]. However, the last publicly reported exposure category estimates for Québec are from 2011 [14]. Moreover, age‐stratified and city‐level estimates are not available.

To inform provincial elimination efforts, we aimed to estimate HIV incidence in Québec and its largest city (Montréal) over 1975–2020, stratified by age, for two key populations: MSM and PWID. We simultaneously estimated key HIV epidemic metrics of prevalence, undiagnosed fraction and time to diagnosis. We achieved this by developing, parameterizing and calibrating a multi‐state back‐calculation mathematical model synthesizing granular surveillance data and capturing the disease's natural progression and treatment and care cascade. These metrics can provide detailed information for communities and health authorities to identify unmet prevention needs and sustainably curb HIV transmission.

## 2 METHODS

### 2.1 Data sources

We obtained surveillance data on AIDS cases (1979–1998) and new HIV diagnoses (2003–2020) from the *Institut national de santé publique du Québec* (INSPQ). The AIDS data are aggregated and stratified by exposure category and sex. The HIV diagnosis data are annual and stratified by region, exposure category, sex, age, self‐reported time since the last negative test and CD4 cell count at diagnosis. The exposure categories are mutually exclusive and assigned hierarchically, with men who had sex with another man classified as MSM and those who injected drugs as PWID. Those who both had sex with another man and injected drugs are categorized separately and not considered here.

### 2.2 Modelling framework

**Figure 1:**
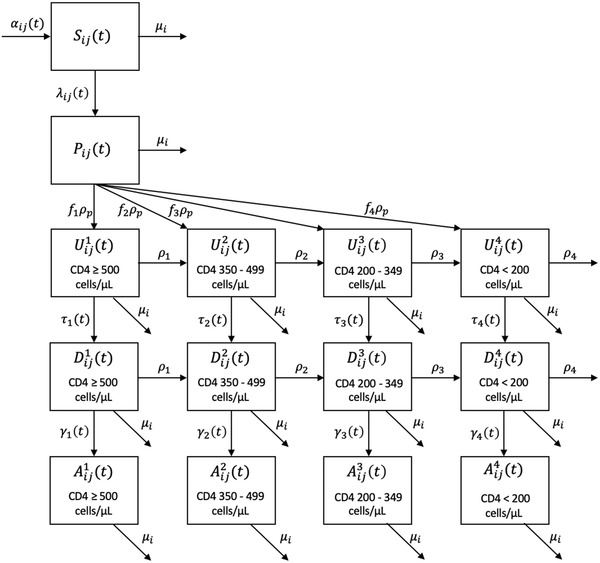
Model flow diagram. The index *i* indicates age groups (15–19, 20–24, 25–29, 30–34, 35–39, 40–44, 45–49, 50–54, 55–59, 60–64 and 65+), *j* indicates sex (male, female and overall) and *k* indicates disease stage (1 = CD4 ≥500 cells/μl; 2 = CD4 350–499 cells/μl; 3 = CD4 200–349 cells/μl; and 4 = CD4 <200 cells/μl). At time *t*, $S_{ij}\left(t\right)$ is the number of susceptible individuals, $P_{ij}\left(t\right)$ is the number of individuals with primary infection, $U_{ij}^{k}\left(t\right)$ is the number of individuals undiagnosed in each of the CD4 cell count compartments, $D_{ij}^{k}\left(t\right)$ is the number of individuals diagnosed and untreated in each of the CD4 cell count compartments and $A_{ij}^{k}\left(t\right)$ is the number of individuals diagnosed who initiated ART in each of the CD4 cell count compartments. See Table S9 for a full description of model parameters.

We developed a deterministic, compartmental mathematical model using Bayesian back‐calculation methods to estimate HIV incidence over 1975–2020 among distinct, open populations of MSM and PWID (active and past injecting history) aged 15–99 years. Extending the approaches of others [15,16], we stratified the population of interest (MSM and PWID) by HIV status and, among people living with HIV (PLHIV), by primary infection and CD4 cell count categories, diagnosed status and treatment status (untreated and ever treated) (Figure 1). The model is further stratified by 5‐year age groups.

Individuals enter the model susceptible to HIV acquisition (Figure 1). Those who acquire HIV progress to the primary stage, where the model assumes no diagnoses occur. After this short period, individuals enter the different CD4 cell count compartments, some at lower counts [17,18]. The subsequent horizontal flow models disease progression through decreasing CD4 cell count. The vertical flow models the clinical care cascade of diagnosis and starting antiretroviral treatment (ART). Individuals exit the model due to all‐cause, injection‐related (active PWID), or AIDS‐related mortality.

The model is solved using an Euler algorithm and 0.01‐year time step, coded in R (v.4.0.3) using a C++ back‐end via the Rcpp library [19, 20, 21]. Analyses are conducted separately by exposure category, sex and region (Montréal and the whole province), with Montréal defined by the public health unit “*région sociosanitaire 06*.”

### 2.3 HIV incidence estimation

We modelled HIV transmission over 1975–2020 using a smooth incidence curve formed with cubic M‐splines [16,22], setting the first coefficient to zero. The incidence rate denominator comprises those at risk of acquiring HIV: all susceptible MSM and actively injecting PWID, respectively. Where feasible, we modelled age‐stratified incidence by estimating a random effect per 10‐year age group. Finally, we varied the number and location of spline knots and determined the best fitting incidence curve considering both the Watanabe–Akaike information criterion (WAIC) and leave‐one‐out cross‐validation information criterion.

### 2.4 Parameterization: demography, testing and ART initiation

Demographic information was drawn from census data, population‐based health surveys and vital statistics (Supplementary Materials). The MSM and PWID (ever injected) population sizes were assumed proportional to the male and total populations, respectively. The active PWID population decreased with time (Figure S1), as informed by local estimates [23, 24, 25]. Disease progression was parameterized using published literature. Where possible, local studies informed the testing and ART parameters. Table S9 details all parameter sources.

We modelled HIV testing and diagnosis among those susceptible to and living with HIV. Testing rates differed by CD4 cell count, reflecting asymptomatic and symptomatic testing. Except for symptomatic diagnoses among those with CD4 <200 cells/μl, testing started in 1985 and subsequently increased over time. It was modelled using a flexible logistic growth function capturing testing trends that reflect treatment advances, testing recommendations and recent empirical estimates. Upon a first positive test, a certain proportion of HIV diagnoses are reported to the surveillance database. Others, at times, may be delayed and classified as such (“delayed report”). The model accounts for these using a reporting fraction varying between 2003–2011 and 2012–2020, as the surveillance system started including those without Québec's universal health insurance in 2012.

ART initiation rates varied by time and CD4 cell count to match guideline changes. These indicated ART for those with CD4 ≤350 cells/μl from 1996 to 2012 [26, 27, 28], ≤500 cells/μl from 2013 to 2015 [27, 28, 29] and all PLHIV from 2016 onward [29]. Accordingly, all ART initiation rates were zero until 1996 (upon highly active ART availability) or until the eligibility criteria indicated use. The rate additionally varied between 1996–2003 and 2004–2012, with a higher rate in the latter period reflecting increasing access and acceptability over time. The rates were fixed and assumed equal across eligible categories.

### 2.5 Model calibration

We calibrated the model to the following outcomes, excluding 2020 data due to uncertainties in how testing disruptions during the COVID‐19 pandemic impacted the observed diagnoses: (1) aggregated AIDS cases (1979–1998); (2) annual HIV diagnoses by sex and age (2003–2019), and CD4 cell count at diagnosis (2013–2019); and (3) annual proportion of diagnoses reporting a negative HIV test result <12 months ago by sex and age (2003–2019). In estimating the age‐specific incidence, we grouped the data by 10‐year age categories to avoid small counts of diagnoses and ensure the stability of estimates. CD4 cell count had missing data (12% and 11% among MSM and 22% and 19% among PWID in Montréal and Québec, respectively), which were assumed to be missing completely at random. Regarding testing history, individuals who were unsure when last tested (<4% of observations) were considered not to have been tested for HIV in the past 12 months. As reported elsewhere [30], initial cross‐validation suggested that self‐reports of a negative test <12 months ago were subject to a telescoping bias. We accounted for this by assuming these self‐reports referred to periods up to 18 months.

We adopted a Bayesian calibration framework. Specifically, we used maximum a posteriori estimation [31] with prior distributions elicited for each unknown parameter (Table S9). Point estimates were obtained by minimizing the negative posterior log‐likelihood of the model. This optimization occurred in two steps. First, we defined a distribution using a Broyden–Fletcher–Goldfarb–Shanno algorithm with starting values from the Nelder–Mead algorithm [32]. Secondly, to approximate the posterior distribution, we performed sampling importance resampling using 50,000 parameter sets from that proposal (i.e. multivariate *t*‐distribution) and resampling 1000 sets without replacement, applying standardized importance weights. We summarized posterior distributions using the median. The 2.5th and 97.5th percentiles approximated 95% credible intervals (CrI).

### 2.6 Additional epidemic metrics

With the estimated incidence curve, we calculated the HIV prevalence (total PLHIV/total population), the fraction undiagnosed (total in primary and undiagnosed compartments/total PLHIV) and the fraction of diagnosed PLHIV that started ART (total in ever treated compartments/total in diagnosed [untreated] and ever treated compartments). Lastly, we calculated the median time to diagnosis annually using period life tables [33]. Each life table began with the total new HIV acquisitions from a given year and, over time, counted diagnoses from each CD4 cell count category or upon AIDS‐related death, assuming those who acquired HIV were subject to that same year's testing rate throughout their lifetime.

### 2.7 Sensitivity analyses

Due to uncertainties in the active PWID population size, we varied the assumed size to assess the impact on mortality. We also varied some of the assumed parameters for disease progression and treatment initiation, as described in the Supplementary Materials (Section 5). Lastly, calibrating up to 2019 data and predicting incidence through 2020 assumes incidence was unaffected by the COVID‐19 pandemic. In sensitivity analyses, we included 2020 data in calibration and assessed the incidence with 0%, 25% and 50% reductions in testing rates over March–December 2020.

### 2.8 Ethics

This study received ethics approval from the *McGill University Research Ethics Board* (REB*#*: *A12‐E84‐18A*). Consent was not necessary as we used aggregated information (from de‐identified and anonymized databases) from routinely collected surveillance data.

## 3 RESULTS

### 3.1 Surveillance data

From 1979 to 1998, 3582 AIDS cases classified as MSM were reported across Québec [34], with 2791 from Montréal [35]. Over that same period, 375 AIDS cases classified as PWID were reported provincially [34], with 300 from Montréal [35]. The new HIV diagnoses reported over 2003–2019 among MSM totalled 3488 and 2286 across Québec and Montréal, respectively. Of these, 23% and 26% reported recently testing negative in those same regions. Among PWID, 390 and 174 new HIV diagnoses were reported in Québec and Montréal, respectively, 10% of which recently tested negative in both regions. Lastly, since 2013, the CD4 ≥500 cells/μl category had the highest proportion of reported new diagnoses, comprising 40% and 43% of MSM diagnoses and 31% and 38% of PWID diagnoses in Québec and Montréal, respectively.

### 3.2 Model selection and fits

**Figure 2:**
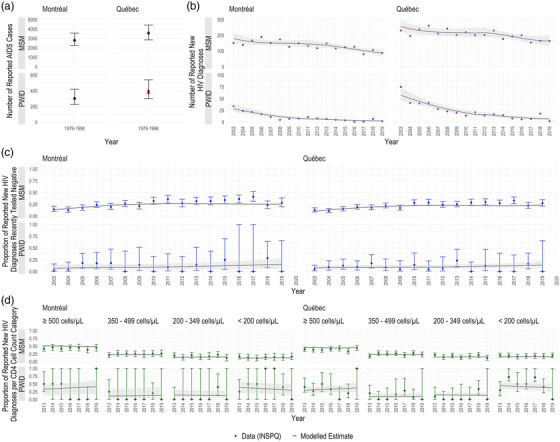
Model fits. Model fits to the calibration outcomes among men who have sex with men (MSM) and people who inject drugs (PWID) in Montréal and the province of Québec: (a) the number of reported AIDS cases; (b) the number of reported new HIV diagnoses; (c) proportion of reported new HIV diagnoses that recently tested negative (<18 months ago); and (d) proportion of reported new HIV diagnoses per CD4 cell count category. The black points and lines display the model‐predicted outcomes, with the black bars and grey bands showing their corresponding 95% credible intervals. The coloured points and bars display the outcomes from the *Institut national de santé publique du Québec* (INSPQ) data and their corresponding 95% confidence intervals, where applicable.

Our calibrated models reproduced the surveillance data and key epidemic features (Figure 2 and Figures S4–S9), with almost all data points falling within the modelled uncertainty bounds. The best‐fitting models had 3–5 knots depending on strata (Table S13). In general, the WAIC did not change substantially across the models explored.

### 3.3 Epidemic trajectory: men who have sex with men

**Figure 3:**
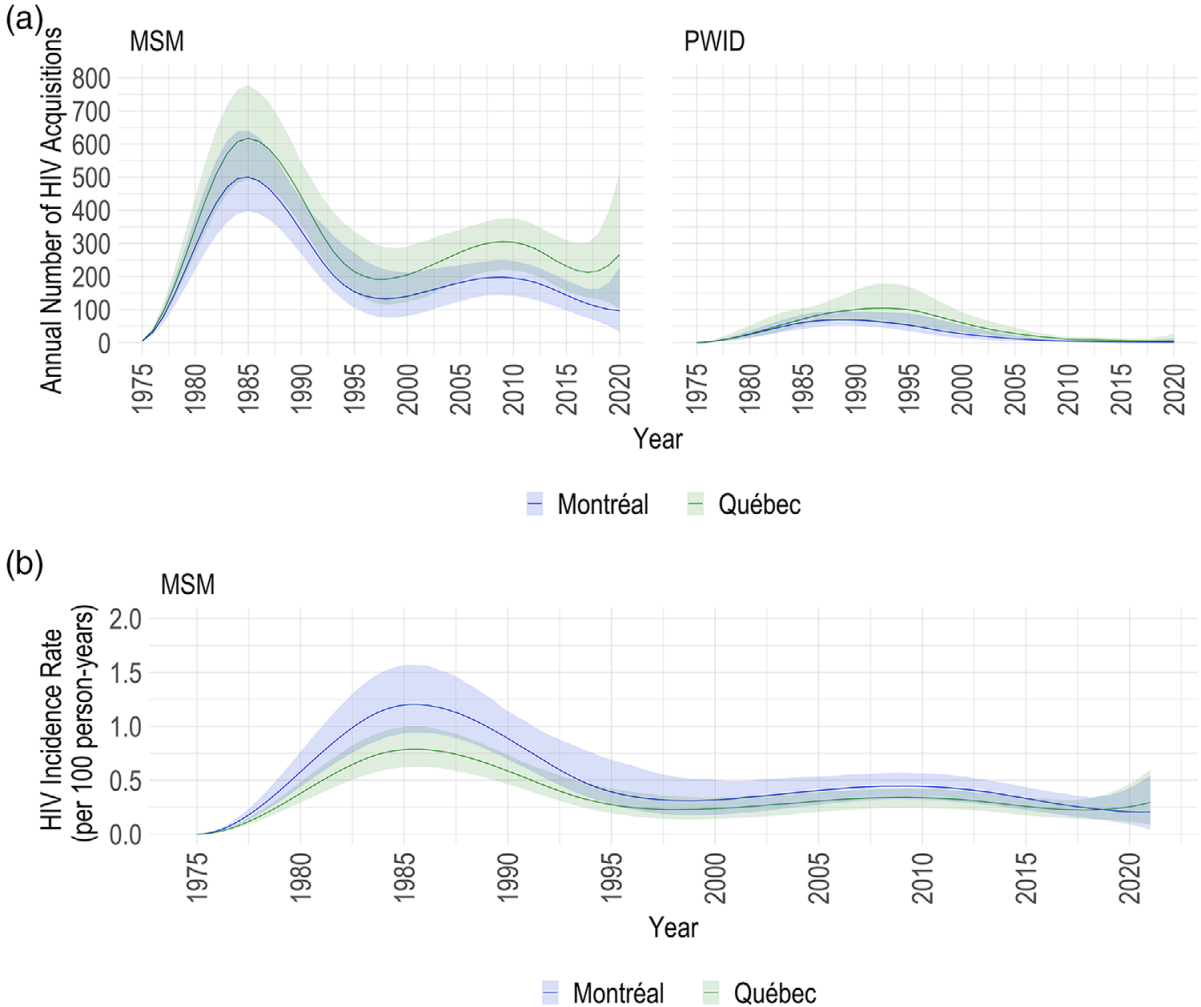
Overall HIV incidence. Estimated HIV incidence over 1975–2020 among men who have sex with men (MSM) and people who injected drugs (PWID) in Montréal and the province of Québec: (a) the annual number of HIV acquisitions among MSM and active PWID; and (b) the HIV incidence rate among MSM. Incidence rates are not presented for PWID due to uncertainties in the denominator (the active PWID population size over time). The coloured lines and bands display the posterior median and 95% credible intervals, respectively, with blue representing estimates from the Montréal region and green representing estimates for the province of Québec.

**Figure 4:**
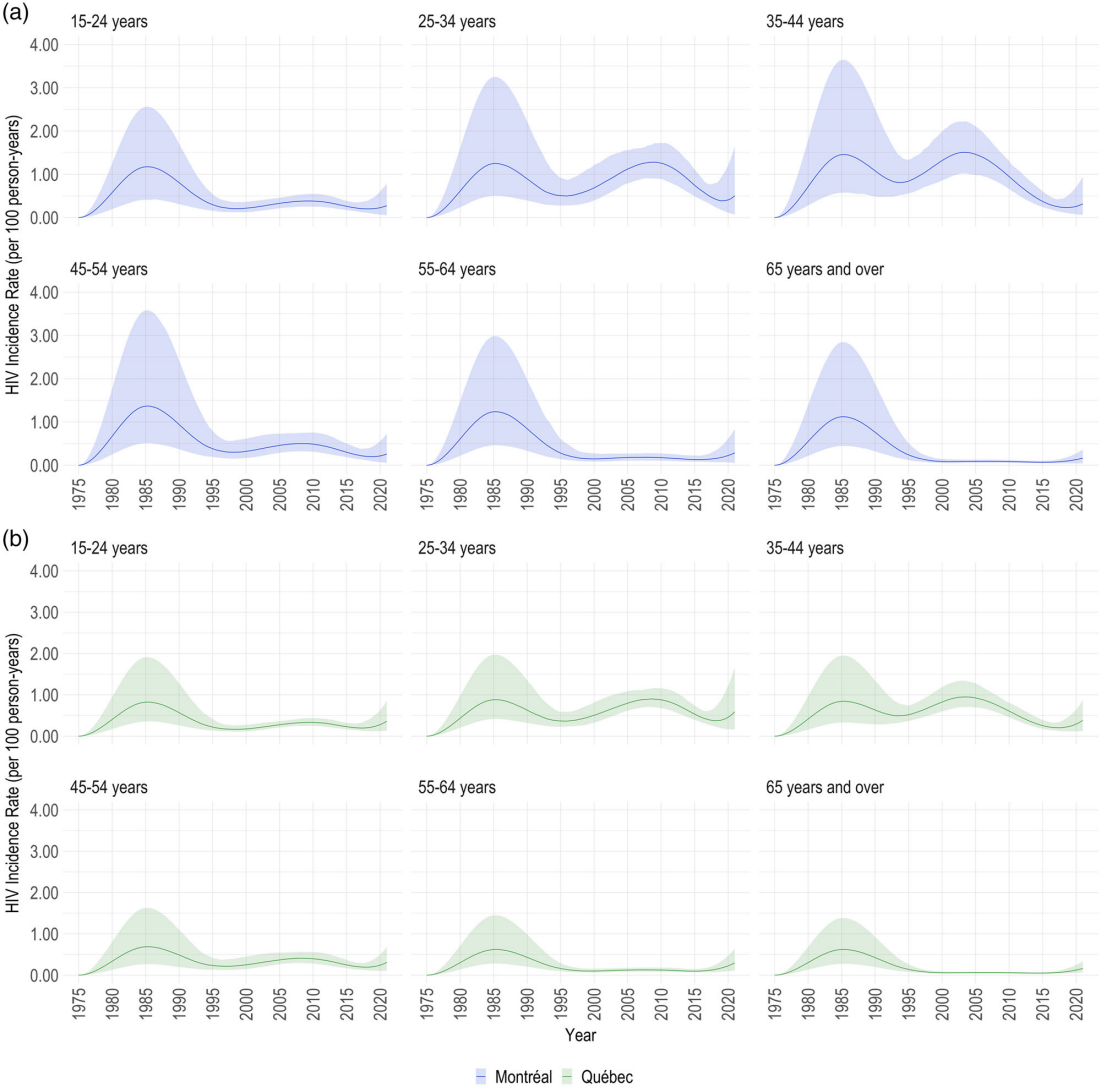
Age‐stratified HIV incidence. Estimated age‐stratified HIV incidence rate over 1975–2020 among men who have sex with men (MSM) in Montréal and the province of Québec, aggregated by 10‐year age groups. The coloured lines and bands display the posterior median and 95% credible intervals, respectively, with blue representing estimates from the Montréal region (panel a) and green representing estimates for the whole province (panel b).

The HIV incidence curves among MSM exhibited two peaks throughout the epidemic. The first, in the mid‐1980s, reached its highest and impacted all ages. Montréal MSM had the highest overall incidence rate of 1.2 per 100 person‐years (PY; 95% CrI: 0.9–1.6 per 100 PY) at its first peak in 1985, with 501 (95% CrI: 397–640) HIV acquisitions that year (Figure 3). The provincial MSM epidemic closely followed this trend, albeit with a smaller peak of 0.8 per 100 PY (95% CrI: 0.6–1.0 per 100 PY), but 617 (95% CrI: 493–778) HIV acquisitions. For more than a decade afterward, incidence among MSM declined. Nevertheless, it again started trending upward by the end of the 1990s. Around then, differences in the estimated age‐stratified incidence became apparent, increasing considerably in those aged 25–34 and 35–44 years (Figure 4 and Figure S10). After reaching an overall rate of 0.4 per 100 PY (95% CrI: 0.3–0.6 per 100 PY) and 0.3 per 100 PY (95% CrI: 0.2–0.4 per 100 PY) in the mid‐to‐late‐2000s in Montréal and Québec, respectively, this trend reversed. Finally, in 2020, incidence potentially increased. At 2020 year‐end, the estimated incidence rate was 0.2 per 100 PY (95% CrI: 0.0–0.5 per 100 PY) and 0.3 (95% CrI: 0.1–0.6 per 100 PY) in Montréal and Québec, with 97 (95% CrI: 33–227) and 266 (95% CrI: 103–508) HIV acquisitions estimated that year in each region, respectively (Table 1 and Figure 3).

**Table 1:** Estimated HIV incidence rate, annual number of HIV acquisitions and percentage of people living with HIV undiagnosed in recent years (2017–2020) among men who have sex with men and people who inject drugs in Montréal and the province of Québec.

Location	Year	Incidence rate (95% CrI) per 100 PY at year end^a^	Annual number of HIV acquisitions (95% CrI)	% PLHIV undiagnosed (95% CrI) at year end
*Men who have sex with men*				
Montréal	2017	0.2 (0.2–0.3)	118 (75–162)	5.3 (3.5–7.3)
	2018	0.2 (0.1–0.4)	108 (65–162)	4.8 (3.1–6.9)
	2019	0.2 (0.1–0.4)	100 (51–181)	4.5 (2.8–6.9)
	2020	0.2 (0.0–0.5)	97 (33–227)	4.3 (2.3–7.2)
Province of Québec	2017	0.2 (0.1–0.3)	212 (136–301)	8.0 (5.7–11.0)
	2018	0.2 (0.1–0.4)	217 (132–330)	7.7 (5.3–10.9)
	2019	0.3 (0.1–0.5)	234 (122–397)	7.6 (5.0–11.2)
	2020	0.3 (0.1–0.6)	266 (103–508)	7.9 (4.6–12.4)
*People who inject drugs (includes active and past injectors)*				
Montréal	2017	–	3 (1–8)	2.1 (0.7–6.2)
	2018	–	3 (1–9)	1.9 (0.6–6.0)
	2019	–	2 (0–11)	1.8 (0.5–6.0)
	2020	–	2 (0–14)	1.6 (0.4–7.1)
Province of Québec	2017	–	6 (3–14)	3.4 (1.6–7.4)
	2018	–	6 (2–15)	3.1 (1.4–7.2)
	2019	–	6 (2–20)	2.9 (1.2–7.2)
	2020	–	6 (1–26)	2.8 (1.0–8.0)

Abbreviations: CrI, credible interval; PLHIV, people living with HIV; PY, person‐years.

^a^ Only presented for men who have sex with men, due to uncertainties in the denominator for people who inject drugs (active injectors).

**Figure 5:**
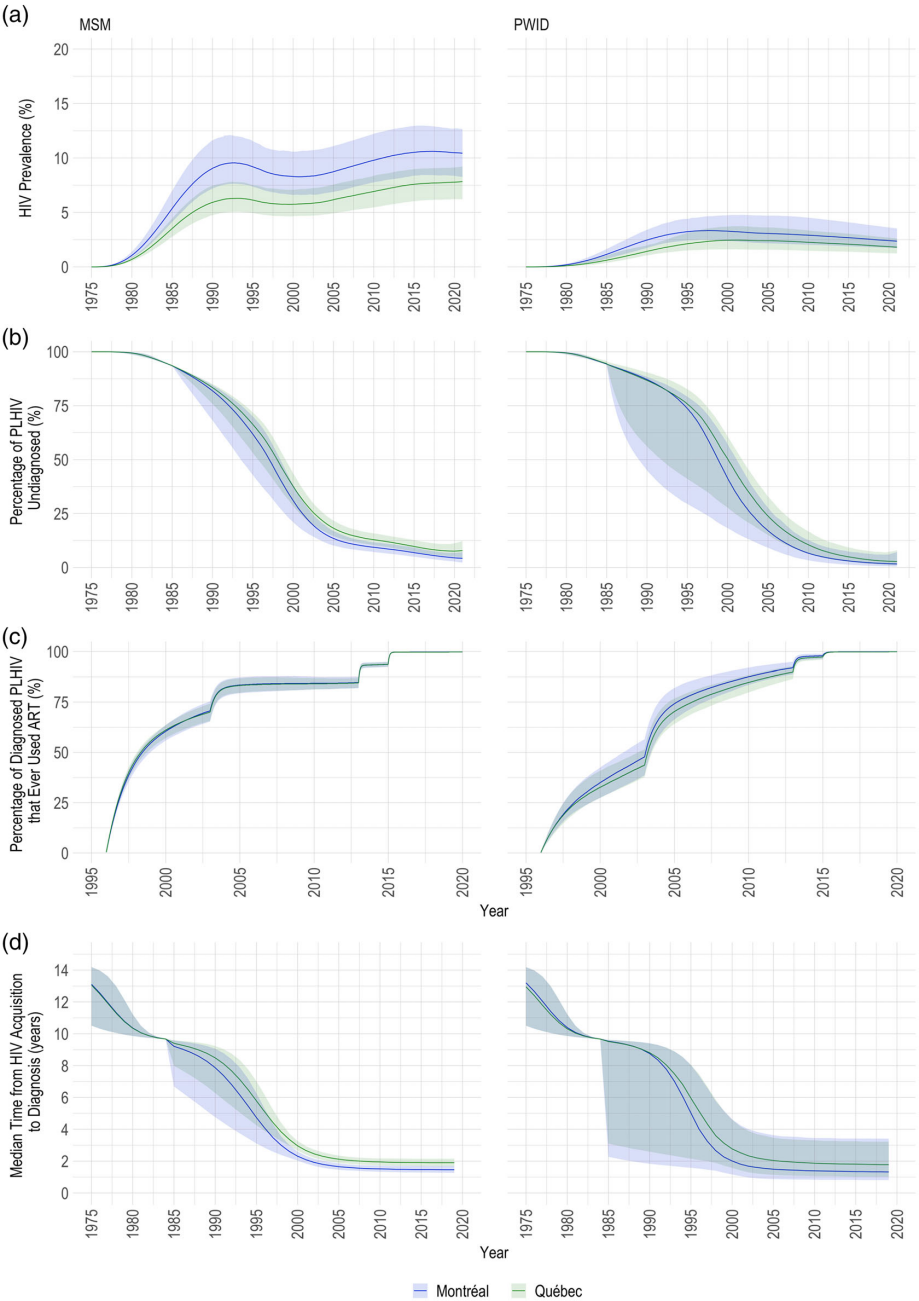
Additional epidemic metrics. Estimated HIV prevalence (panel a), percentage of people living with HIV (PLHIV) undiagnosed (panel b), percentage of diagnosed PLHIV that ever used antiretroviral treatment (ART; panel c) and the median time from HIV acquisition to diagnosis (panel d) over 1975–2020 among men who have sex with men (MSM) and people who ever injected drugs (PWID) in Montréal and the province of Québec. The coloured lines and bands display the posterior median and 95% credible intervals, respectively, with blue representing estimates from the Montréal region and green representing estimates for the whole province. Note: Estimates of the median time from HIV acquisition to diagnosis are presented up 2019 year‐end due to uncertainties in testing rates at the start of the COVID‐19 pandemic in 2020.

HIV prevalence followed a trend similar to incidence, peaking in the early 1990s at 9.5% (95% CrI: 7.6–12.0%) in Montréal and 6.3% (95% CrI: 5.1–7.8%) in Québec (Figure 5). After dipping slightly, following the peak in AIDS deaths (Figure S18), the prevalence continued rising until the mid‐to‐late‐2010s. These trends were largely mimicked across age groups, except for those <35 years, whose prevalence stabilized (Figure S11). By 2020 year‐end, prevalence in MSM was estimated at 10.4% (95% CrI: 8.3–12.6%) in Montréal and 7.8% (95% CrI: 6.2–9.2%) provincially (Figure 5).

The undiagnosed fraction decreased upon testing availability in 1985 (Figure 5). Among MSM, an estimated 4.3% (95% CrI: 2.3–7.2%) and 7.9% (95% CrI: 4.6–12.4%) of PLHIV in Montréal and Québec, respectively, were undiagnosed at 2020 year‐end (Table 1 and Figure 5). Correspondingly, up to the start of 2020, the median time to diagnosis decreased to 1.5 (95% CrI: 1.3–1.7 years) and 1.9 years (95% CrI: 1.7–2.1 years) in those same regions (Figure 5). Important differences in the percentage undiagnosed were observed in younger MSM, especially 15–24 year‐olds, among whom 21.2% in Montréal (95% CrI: 8.5–39.0%) and 31.1% in Québec (95% CrI: 17.0–47.5%) remained undiagnosed by the end of 2020 (Figure S12). Lastly, the percentage of diagnosed PLHIV that started ART increased over time, reaching almost 100% (Figure 5 and Figure S13).

Sensitivity analyses incorporating testing reductions and including 2020 data in calibration resulted in lower incidence unless testing was reduced by 50%, where it was similar to our main results (Figures S19–S21 and Table S14). The undiagnosed fraction was also lower unless testing was reduced by 25% and 50%, where it was similar to and possibly higher than our main estimates.

### 3.4 Epidemic trajectory: people who inject drugs

The HIV incidence curve among actively injecting PWID had one peak. The highest annual incidence occurred in the late‐1980s‐to‐early‐1990s with 70 (95% CrI: 53–96) and 105 (95% CrI: 56–179) HIV acquisitions in Montréal and the province, respectively. After these peaks, incidence trended downward (Figure 3). In 2020, 2 (95% CrI: 0–14) and 6 (95% CrI: 1–26) HIV acquisitions were estimated in Montréal and Québec, respectively (Table 1 and Figure 3). Similar regional trends were seen among active male and female PWID (Figure S14), but with wider uncertainty ranges and more acquisitions estimated among males. Due to uncertainties in the time‐varying active PWID population size, incidence rates are not presented for this population.

HIV prevalence among lifetime PWID increased until the late‐1990s‐early‐2000s, reaching 3.3% (95% CrI: 2.5–4.7%) and 2.4% (95% CrI: 1.6–3.7%) in Montréal and Québec, respectively (Figure 5). As AIDS‐related deaths diminished (Figure S18) and incidence somewhat levelled off, prevalence stabilized and decreased slowly to 2.4% (95% CrI: 1.7–3.5%) in Montréal and 1.8% (95% CrI: 1.2–2.6%) in Québec by 2020 year‐end. When stratified by sex, similar trends were estimated (Figure S15).

The PWID population also saw large declines in the undiagnosed fraction (Figure 5 and Figure S15). By 2020 year‐end, 1.6% (95% CrI: 0.4–7.1%) and 2.8% (95% CrI: 1.0–8.0%) of PLHIV were undiagnosed in Montréal and Québec, respectively (Table 1 and Figure 5). The median time to diagnosis also decreased up to 2020, reaching 1.3 (95% CrI: 0.8–3.4) and 1.8 (95% CrI: 1.0–3.2) years in those regions (Figure 5). Finally, the percentage of diagnosed PLHIV that started ART similarly increased to almost 100% (Figure 5 and Figure S15).

None of these results were appreciably impacted by the active PWID population size or ART initiation rates. Including 2020 data in calibration did affect results (Figures S19–S21 and Table S14). Among Montréal PWID, there were potentially fewer HIV acquisitions in 2020, even with testing reduced by 50%. In contrast, acquisitions and the undiagnosed fraction increased among PWID across Québec as, in this case, the observed diagnoses substantially increased from 2019 to 2020.

## 4 DISCUSSION

Accurately measuring progress towards elimination is challenging, yet, synthesizing complex information from surveillance and other data can help overcome some limitations. We developed a multi‐state back‐calculation Bayesian model and estimated key elimination indicators of new HIV acquisitions, the burden of disease and the cascade of care. Our findings suggest that—among key populations historically bearing the highest burden—HIV incidence has drastically decreased and, in MSM, may have reached near 0.1 per 100 PY, the proposed elimination threshold for HIV as a public health threat. However, the goal of zero new HIV acquisitions was not reached and could be further from sight due to HIV service interruptions during the COVID‐19 pandemic. While less than 14 HIV acquisitions could have occurred among PWID in Montréal, we projected 97 (95% CrI: 33–227) HIV acquisitions in 2020 among MSM. Across the province, 6 (95% CrI: 1–26) acquisitions in PWID and 266 (95% CrI: 103–508) in MSM were projected.

Our results also point to rapid and robust improvements in the treatment and care cascade. The fraction of undiagnosed PLHIV is estimated at 4.3% (95% CrI: 2.3–7.2%) among Montréal's MSM, a finding supported by the *Engage* cohort [36,37], and 1.6% (95% CrI: 0.4–7.1%) among PWID. Therefore, Montréal has already reached the 2025 goal of 95% diagnosed among these populations. However, setbacks could have occurred since the COVID‐19 pandemic began. Provincially, slightly higher proportions were unaware of their status: 7.9% of MSM (95% CrI: 4.6–12.4%) and 2.8% of PWID (95% CrI: 1.0–8.0%). Previous studies suggested PLHIV residing outside of urban areas could face additional barriers in accessing healthcare, including stigma, further distance to providers coupled with inadequate transportation and local providers being less experienced with HIV and/or key populations [38,39]. There were also proportionally more young MSM unaware of their status, highlighting their unmet prevention needs. Lower diagnosis coverage among younger PLHIV also reflects HIV's transmission dynamics: young people have a higher incidence and smaller cumulative testing exposure [33,40]. Concomitant with diagnosis coverage improvements, we estimated shortened diagnostic delays. Still, further reductions could impact onward HIV transmission if linkage to care is prompt.

Finally, almost 100% of diagnosed PLHIV have taken ART—in line with empirical estimates from a Montréal clinical cohort [41] and *Engage* [36], suggesting high proportions of diagnosed PLHIV are being linked to care. While this does not speak to current use or levels of viral suppression, these studies did estimate >95% of those diagnosed were on ART and, of those, >88% were virally suppressed [36,41]. Although we do not model the impact of ART on transmission directly, its successful scale‐up likely played a part in the incidence declines over the last decade. Moreover, pre‐exposure prophylaxis (PrEP) was introduced for MSM in 2013 and could have accelerated incidence reductions, especially amidst increased usage in recent years [42,43], where coverage (i.e. those currently on PrEP) rose from 5% to 10% between 2017 and 2018 in Montréal [43].

Given the richness and granularity of Québec's HIV surveillance data, we tailored our model to its intricacies. PHAC's back‐calculation method is general to accommodate the different provincial data streams. We explicitly describe the clinical course of HIV, allow testing rates per disease stage and calibrate to multiple sources of recency information, all of which could more accurately estimate incidence. Moreover, modelling ART captures reduced mortality and improves our prevalence estimation. The trends in incidence did follow those modelled by PHAC until 2011 among MSM and PWID in Canada [14]. Their 2011 Québec‐specific estimates suggested 425 (290–560) and 9690 (7880–11,500) MSM acquired and were living with HIV that year, respectively [14]. Among PWID, they estimated that there were 60 (40–80) HIV acquisitions and 3000 (2400–3600) PLHIV [14]. In that same year, we projected fewer HIV acquisitions (297, 95% CrI: 215–370 in MSM and 10, 95% CrI: 6–17 in PWID) and a smaller population of PLHIV (6928, 95% CrI: 5469–8334 MSM and 1046, 95% CrI: 689–1544 PWID). Despite possibly underestimating incidence in the mid‐1990s, from 2000 onward, our MSM incidence rate and prevalence estimates reassuringly aligned with that observed by cohort studies in Montréal (Figures S16 and S17).

This study has several limitations. First, the paucity of data informing the active PWID population sizes, and the potential underrepresentation of active PWID in the available estimates, necessitated additional assumptions in this regard. Therefore, we did not provide HIV incidence rates for PWID and focused on HIV acquisitions. Moreover, we lacked appropriate PWID cross‐validation data as local studies [44,45] have focused solely on individuals actively injecting, whereas we modelled all people who ever injected drugs. Further, despite modelling reporting delays, we did not have strong estimates to inform them. However, we believe most transmissions occurring and diagnosed in Québec have a high probability of being reported without delay, and our calibrated reporting fractions reflect this (approximately 70–80% across populations). Concerning demography, we do not capture the migration of PLHIV. Not unlike other models, this could affect the prevalence and undiagnosed fraction, the extent to which depends on the level and direction of the net migration of PLHIV in the MSM and PWID populations. In our setting, we expect net migration to be small and our estimates to be robust. Our cross‐validation to the Engage study's prevalence is also reassuring. Finally, HIV testing could have fluctuated in recent years. Potential increases alongside PrEP scale‐up, for example, could identify more undiagnosed PLHIV through consultations, leading our model to overestimate incidence [46]. Despite this, the number of diagnoses remained relatively stable. On the other hand, the COVID‐19 pandemic disrupted testing in 2020, and the extent to which is not yet known [47]. Thus, we explored this in sensitivity analyses.

## 5 CONCLUSIONS

The UNAIDS Fast‐Track City goals are ambitious. Meeting and maintaining targets of zero new HIV acquisitions, 95% diagnosis coverage, 95% treatment coverage and 95% viral suppression requires an actionable response and epidemic monitoring. Leveraging detailed surveillance data complemented by other sources, we provided up‐to‐date estimates of important epidemic metrics among key populations in Montréal and across Québec. This work demonstrates how such back‐calculation mathematical models can be applied in a Canadian context to estimate incidence, compared to only monitoring diagnoses, and other jurisdictions with similar data could do the same. Identifying effective policies to progress towards and sustain elimination is crucial, especially in light of the possible setbacks from the decline in prevention service access and use during the COVID‐19 pandemic [47]. Future work exploring the population‐level impact of interventions that could be scaled‐up, such as PrEP, could inform these.

## COMPETING INTERESTS

JC has investigator‐sponsored research grants from Gilead Sciences Canada and ViiV Healthcare. He has also received financial support for conference travel and advisory work for Gilead Sciences Canada, Merck Canada and ViiV Healthcare. MM‐G reports an investigator‐sponsored research grant from *Gilead Sciences Inc*., outside of the submitted work, and contractual arrangements from the *World Health Organization*, the *Joint United Nations Programme on HIV/AIDS* (UNAIDS), the *Institut national de santé publique du Québec* (INSPQ) and the *Institut d'excellence en santé et services sociaux* (INESSS) also outside of the submitted work. CT has investigator‐sponsored research grants from Merck and Gilead, and has received financial support for advisory work and conferences from Gilead, Merck, Medicago, Astra‐Zeneca and GSK. MA is a recipient of a Foundation grant from the Canadian Institutes of Health Research outside the submitted work and reports contractual arrangements with the Public Health Agency of Canada and the Ministère de la santé et des services sociaux du Québec supporting the study that provided the data on people who inject drugs used in the present study.

## AUTHORS’ CONTRIBUTIONS

CMD, MM‐G and JC contributed to the study's conception and design. RB, MA and GL were involved in the design and data collection. Analyses were performed by CMD, with support from MM‐G. The manuscript was drafted by CMD. All authors contributed to the interpretation of the results and reviewed the manuscript for important intellectual content. Overall supervision for this project was provided by MM‐G and JC. All authors approved the final manuscript.

## FUNDING

CMD is supported by a doctoral award from the Fonds de recherche du Québec—Santé (FRQS). CLD received a PhD trainee fellowship from the Canadian Network on Hepatitis C. The Canadian Network on Hepatitis C is funded by a joint initiative of the Canadian Institutes of Health Research (CIHR) (NHC‐142832) and the Public Health Agency of Canada. CLD also received a doctoral award from the FRQS. SM is supported by a New Investigator Award from CIHR and the Ontario HIV Treatment Network. Grants from the CIHR and the Canadian Foundation for AIDS Research to MM‐G. MM‐G's research programme is funded by the *Tier 2 Canada Research Chair* in *Population Health Modeling*. CT is the Pfizer/University of Montreal Chair on HIV Translational Research.

## DISCLAIMER

This work is the sole product of the authors and has never been submitted for publication.

## Supporting information


**Figure S1**. Modeled active PWID population size aged ≥15 in Montréal and Québec over 1975–2020.
**Figure S2**. All cause‐mortality rates for Québec by age group.
**Figure S3**. Diagram of the main inter‐compartmental flows for HIV testing histories.
**Figure S4**. Model fits to the age‐stratified calibration outcomes among men who have sex with men in Montréal: A) number of reported AIDS cases; B) number of reported new HIV diagnoses by age group; C) proportion of reported new HIV diagnoses that recently tested negative by age group; and D) proportion of reported new HIV diagnoses per CD4 cell count category and age group. The black points and lines display the model‐predicted outcomes, with the black bars and grey bands showing their corresponding 95% credible intervals. The coloured points and bars display the outcomes from the *Institut national de santé publique du Québec* (INSPQ) data and their corresponding 95% confidence intervals, where applicable.
**Figure S5**. Model fits to the age‐stratified calibration outcomes among men who have sex with men in the province of Québec: A) number of reported AIDS cases; B) number of reported new HIV diagnoses by age group; C) proportion of reported new HIV diagnoses that recently tested negative by age group; and D) proportion of reported new HIV diagnoses per CD4 cell count category and age group. The black points and lines display the model‐predicted outcomes, with the black bars and grey bands showing their corresponding 95% credible intervals. The coloured points and bars display the outcomes from the *Institut national de santé publique du Québec* (INSPQ) data and their corresponding 95% confidence intervals, where applicable.
**Figure S6**. Model fits to the calibration outcomes among males who injected drugs in Montréal: A) number of reported AIDS cases; B) number of reported new HIV diagnoses; C) proportion of reported new HIV diagnoses that recently tested negative; and D) proportion of reported new HIV diagnoses per CD4 cell count category. The black points and lines display the model‐predicted outcomes, with the black bars and grey bands showing their corresponding 95% credible intervals. The coloured points and bars display the outcomes from the *Institut national de santé publique du Québec* (INSPQ) data and their corresponding 95% confidence intervals, where applicable.
**Figure S7**. Model fits to the calibration outcomes among females who injected drugs in Montréal: A) number of reported AIDS cases; B) number of reported new HIV diagnoses; C) proportion of reported new HIV diagnoses that recently tested negative; and D) proportion of reported new HIV diagnoses per CD4 cell count category. The black points and lines display the model‐predicted outcomes, with the black bars and grey bands showing their corresponding 95% credible intervals. The coloured points and bars display the outcomes from the *Institut national de santé publique du Québec* (INSPQ) data and their corresponding 95% confidence intervals, where applicable.
**Figure S8**. Model fits to the calibration outcomes among males who injected drugs in the province of Québec: A) number of reported AIDS cases; B) number of reported new HIV diagnoses; C) proportion of reported new HIV diagnoses that recently tested negative; and D) proportion of reported new HIV diagnoses per CD4 cell count category. The black points and lines display the model‐predicted outcomes, with the black bars and grey bands showing their corresponding 95% credible intervals. The coloured points and bars display the outcomes from the *Institut national de santé publique du Québec* (INSPQ) data and their corresponding 95% confidence intervals, where applicable.
**Figure S9**. Model fits to the calibration outcomes among females who injected drugs in the province of Québec: A) number of reported AIDS cases; B) number of reported new HIV diagnoses; C) proportion of reported new HIV diagnoses that recently tested negative; and D) proportion of reported new HIV diagnoses per CD4 cell count category. The black points and lines display the model‐predicted outcomes, with the black bars and grey bands showing their corresponding 95% credible intervals. The coloured points and bars display the outcomes from the *Institut national de santé publique du Québec* (INSPQ) data and their corresponding 95% confidence intervals, where applicable.
**Figure S10**. Estimated age‐stratified annual number of HIV acquisitions over 1975–2020 among men who have sex with men in Montréal and the province of Québec, with incidence estimated per 10‐year age group. The coloured lines and bands display the posterior median and 95% credible intervals, respectively, with blue representing estimates from the Montréal region (panel A) and green representing estimates for the whole province (panel B).
**Figure S11**. Estimated age‐stratified HIV prevalence over 1975–2020 among men who have sex with men (MSM) in Montréal and the province of Québec, by 10‐year age groups. The coloured lines and bands display the posterior median and 95% credible intervals, respectively, with blue representing estimates from the Montréal region (panel A) and green representing estimates for all of Québec (panel B).
**Figure S12**. Estimated age‐stratified percentage of people living with HIV (PLHIV) undiagnosed over 1975–2020 among men who have sex with men (MSM) in Montréal and the province of Québec, by 10‐year age groups. The coloured lines and bands display the posterior median and 95% credible intervals, respectively, with blue representing estimates from the Montréal region (panel A) and green representing estimates for all of Québec (panel B).
**Figure S13**. Estimated age‐stratified percentage of diagnosed people living with HIV (PLHIV) that ever used antiretroviral treatment (ART) over 1975–2020 among men who have sex with men (MSM) in Montréal and the province of Québec, by 10‐year age groups. The coloured lines and bands display the posterior median and 95% credible intervals, respectively, with blue representing estimates from the Montréal region (panel A) and green representing estimates for all of Québec (panel B).
**Figure S14**. Estimated annual number of HIV acquisitions over 1975–2020 among active females (panel A) and males (panel B) who injected drugs (PWID) in Montréal and the province of Québec. The coloured lines and bands display the posterior median and 95% credible intervals, respectively, with blue representing estimates from the Montréal region and green representing estimates for all of Québec.
**Figure S15**. Estimated HIV prevalence (panel A), percentage of people living with HIV (PLHIV) undiagnosed (Panel B), percentage of diagnosed PLHIV that ever used antiretroviral treatment (ART; panel C), and average time from HIV acquisition to diagnosis (panel D) over 1975–2020 among females and males who ever injected drugs (PWID) overall in Montréal and the province of Québec. The coloured lines and bands display the posterior median and 95% credible intervals, respectively, with blue representing estimates from the Montréal region and green representing estimates for all of Québec.
**Figure S16**. Estimated HIV incidence rate over 1975–2020 among men who have sex with men (MSM) in Montréal and the province of Québec. The coloured lines and bands display the posterior median and 95% credible intervals, respectively, with blue representing estimates from the Montréal region and green representing estimates for the province of Québec. The points and bars display the Omega study41 and Engage (Lambert G, personal communication, Dec. 2021) incidence rate estimates and corresponding 95% confidence intervals, respectively.
**Figure S17**. Estimated HIV prevalence (panel A), percentage of people living with HIV (PLHIV) undiagnosed (panel B), and percentage of diagnosed PLHIV that ever used antiretroviral treatment (ART; panel C) including cross‐validation data over 1975–2020 among men who have sex with men (MSM) in Montréal and the province of Québec. The coloured lines and bands display the posterior median and 95% credible intervals, respectively, with blue representing estimates from the Montréal region and green representing estimates for all of Québec. The points and bars in 2007 and 2010 display the estimates from Argus and their corresponding 95% confidence intervals, respectively. The points and bars from 2018 display the Engage estimates and corresponding 95% confidence intervals, respectively.
**Figure S18**. Estimated counts of AIDS‐related mortality over 1975–2020 among men who have sex with men (MSM) and people who injected drugs (PWID) in Montréal and the province of Québec: A) the annual number of AIDS‐related deaths; and B) the cumulative number of AIDS‐related deaths. The coloured lines and bands display the posterior median and 95% credible intervals, respectively, with blue representing estimates from the Montréal region and green representing estimates for the province of Québec.
**Figure S19**. Estimated HIV incidence over 1975–2020 among men who have sex with men (MSM) and people who injected drugs (PWID) in Montréal and the province of Québec when 2020 data are excluded from model calibration and testing rates are not reduced during the COVID‐19 pandemic: A) the annual number of HIV acquisitions among MSM and active PWID; and B) the HIV incidence rate among MSM. Incidence rates are not presented for PWID due to uncertainties in the denominator (the active PWID population size over time). The coloured lines and bands display the posterior median and 95% credible intervals, respectively, with blue representing estimates from the Montréal region and green representing estimates for the province of Québec.
**Figure S20**. Estimated HIV incidence over 1975–2020 among men who have sex with men (MSM) and people who injected drugs (PWID) in Montréal and the province of Québec when 2020 data are excluded from model calibration and testing rates are reduced by 25% during the COVID‐19 pandemic (March 2020‐year‐end): A) the annual number of HIV acquisitions among MSM and active PWID; and B) the HIV incidence rate among MSM. Incidence rates are not presented for PWID due to uncertainties in the denominator (the active PWID population size over time). The coloured lines and bands display the posterior median and 95% credible intervals, respectively, with blue representing estimates from the Montréal region and green representing estimates for the province of Québec.
**Figure S21**. Estimated HIV incidence over 1975–2020 among men who have sex with men (MSM) and people who injected drugs (PWID) in Montréal and the province of Québec when 2020 data are excluded from model calibration and testing rates are reduced by 50% during the COVID‐19 pandemic (March 2020‐year‐end): A) the annual number of HIV acquisitions among MSM and active PWID; and B) the HIV incidence rate among MSM. Incidence rates are not presented for PWID due to uncertainties in the denominator (the active PWID population size over time). The coloured lines and bands display the posterior median and 95% credible intervals, respectively, with blue representing estimates from the Montréal region and green representing estimates for the province of Québec.
**Table S1**. Population size of Québec in 1975 by sex and age.
**Table S2**. Population size and exponential growth factor of Québec over 1975–2020 by sex.
**Table S3**. Population size of the administrative region of Montréal in 1986 by sex and age.
**Table S4**. Population size and exponential growth factor of the Montréal administrative region over 1986–2020 by sex.
**Table S5**. Estimates of the MSM and PWID population sizes in Québec and Montréal by sex.
**Table S6**. Probability of injection drug use initiation by sex and age group for Québec and Montréal, assuming a zero‐truncated negative binomial distribution.
**Table S7**. Estimated size of the active PWID population aged 15–64 years in Montréal and Québec by sex.
**Table S8**. Proportion of the population ≥15 years assumed to belong to each exposure category over time by sex in Québec and Montréal
**Table S9**. Model parameter values and prior distributions used to estimate HIV incidence among MSM, PWID, and heterosexual populations in Montréal and Québec.
**Table S10**. Local data sources used to inform MSM and PWID model parameters.
**Table S11**. Incidence M‐spline scenarios for knot placement.
**Table S12**. Outcomes used for model calibration and cross‐validation.
**Table S13**. Number and placement of knots included in incidence M‐spline of final models.
**Table S14**. Estimated HIV incidence, annual number of acquisitions, and percentage of people living with HIV undiagnosed in recent years (2017–2020) among men who have sex with men and people who inject drugs in Montréal and the province of Québec when 2020 data are included in model calibration and reductions in testing rates are explored.

## Data Availability

The data that support the findings of this study are available from the *Institut national de santé publique du Québec* (INSPQ). Restrictions do apply to the data availability. The INSPQ permitted the use of these data for this study. Data are available from the authors upon reasonable request and additional approval from the INSPQ.
