# Supplementary material for: Measuring progress towards reaching zero new HIV acquisitions among key populations in Québec (Canada) using routine surveillance data: a mathematical modelling study
Source: J Int AIDS Soc. 2022 Sep 1;25(9):e25994. doi: 10.1002/jia2.25994 (PMC9437443; doi:10.1002/jia2.25994)
Supplement: Supplementary file 1 — Figure S1. Modeled active PWID population size aged ≥15 in Montréal and Québec over 1975–2020. Figure S2. All cause‐mortality rates for Québec by age group. Figure S3. Diagram of the main inter‐compartmental flows for HIV testing histories. Figure S4. Model fits to the age‐stratified calibration outcomes among men who have sex with men in Montréal: A) number of reported AIDS cases; B) number of reported new HIV diagnoses by age group; C) proportion of reported new HIV diagnoses that recently tested negative by age group; and D) proportion of reported new HIV diagnoses per CD4 cell count category and age group. The black points and lines display the model‐predicted outcomes, with the black bars and grey bands showing their corresponding 95% credible intervals. The coloured points and bars display the outcomes from the Institut national de santé publique du Québec (INSPQ) data and their corresponding 95% confidence intervals, where applicable. Figure S5. Model fits to the age‐stratified calibration outcomes among men who have sex with men in the province of Québec: A) number of reported AIDS cases; B) number of reported new HIV diagnoses by age group; C) proportion of reported new HIV diagnoses that recently tested negative by age group; and D) proportion of reported new HIV diagnoses per CD4 cell count category and age group. The black points and lines display the model‐predicted outcomes, with the black bars and grey bands showing their corresponding 95% credible intervals. The coloured points and bars display the outcomes from the Institut national de santé publique du Québec (INSPQ) data and their corresponding 95% confidence intervals, where applicable. Figure S6. Model fits to the calibration outcomes among males who injected drugs in Montréal: A) number of reported AIDS cases; B) number of reported new HIV diagnoses; C) proportion of reported new HIV diagnoses that recently tested negative; and D) proportion of reported new HIV diagnoses per CD4 cell count cate [file JIA2-25-e25994-s001.docx]

**Measuring progress towards reaching zero new HIV acquisitions among key populations in Québec using routine surveillance data: a mathematical modeling study**

**Supplementary Materials**

Carla M Doyle^1^, Joseph Cox^1,2,3^, Rachael M Milwid^1^, Raphaël Bitera^4^, Charlotte Lanièce Delaunay^1,3^, Michel Alary^4,5,6^, Gilles Lambert^2^, Cécile Tremblay^7,8^, Sharmistha Mishra^9,10,11^, Mathieu Maheu-Giroux^1§^

^1^Department of Epidemiology and Biostatistics, School of Population and Global Health, McGill University, Montréal, QC

^2^Direction Régionale de Santé Publique de Montréal, Montréal, QC

^3^Clinical Outcomes Research and Evaluation, Research Institute - McGill University Health Centre, Montréal, QC

^4^Institut national de santé publique du Québec, Québec, QC

^5^Centre de recherche du CHU de Québec – Université Laval, Québec, QC

^6^Département de médecine sociale et préventive, Université Laval, Québec, QC

^7^Centre de Recherche du Centre Hospitalier de l'Université de Montréal (CRCHUM), Montréal, QC

^8^Department of Microbiology Infectiology and Immunology, University of Montréal, Montréal, QC

^9^Department of Medicine, St. Michael's Hospital, University of Toronto, Toronto, ON

^10^Institute of Medical Sciences, University of Toronto, Toronto, ON

^11^Institute of Health Policy Management and Evaluation, Dalla Lana School of Public Health, University of Toronto, Toronto, ON

**Table of Contents**

[1. Model equations 1](#_Toc109227736)

[2. Model demography 1](#_Toc109227737)

[3. Model parameterization 7](#_Toc109227738)

[4. Model calibration and cross-validation 12](#_Toc109227739)

[5. Final models 15](#_Toc109227740)

[6. Additional figures of model fits (calibration outcomes) 16](#_Toc109227741)

[7. Additional results: main analyses 25](#_Toc109227742)

[8. Additional results: sensitivity analyses 32](#_Toc109227743)

[9. References 37](#_Toc109227744)

# Model equations

Let *i* indicate age group (15-19, 20-24, 25-29, 30-34, 35-39, 40-44, 45-49, 50-54, 55-59, 60-64, 65+), *j* indicate sex (male, female, overall), *t* indicate time in years, and *k* indicate CD4 cell count compartment (CD4≥500 cells/µL, CD4 350-499 cells/µL, CD4 200-349 cells/µL, and CD4<200 cells/µL). The following system of ordinary differential equations (with parameters as defined in Table S9) describes the occurrence of events over time *t* in the model.

1. HIV-susceptible (S) individuals are modeled according to:

$$\begin{aligned} \frac{dS_{ij}\left( t \right)}{dt}=\alpha_{ij}(t)-\lambda_{ij}\left( t \right)\delta_{j}(t)S_{ij}\left( t \right)- \mu_{i}(t)S_{ij}\left( t \right) \#\left( 1 \right) \end{aligned}$$

where $\delta_{j}(t)$ is a weight to obtain the number of susceptible individuals that are at-risk of HIV acquisition.

1. The number of people in the primary infection phase (P) are modeled according to:

$$\begin{aligned} \frac{dP_{ij}\left( t \right)}{dt}=\lambda_{ij}\left( t \right){\delta_{j}(t)S}_{ij}\left( t \right)-\left( \rho_{p}+\mu_{i}(t) \right)P_{ij}\left( t \right)\#\left( 2 \right) \end{aligned}$$

1. The number of undiagnosed individuals (U) in the different CD4^+^ cell count compartments *k* are modeled according to:

$$\begin{aligned} \frac{dU_{ij}^{k}\left( t \right)}{dt}=\rho_{p}f_{k}P_{ij}\left( t \right)-\left( \tau_{k}\left( t \right)+\rho_{k}+\mu_{i}(t) \right)U_{ij}^{k}\left( t \right); if k=1 \#\left( 3.1 \right) \end{aligned}$$

$$\begin{aligned} \frac{dU_{ij}^{k}\left( t \right)}{dt}=\rho_{p}f_{k}P_{ij}\left( t \right)+\rho_{\left( k-1 \right)}U_{ij}^{k-1}\left( t \right)-\left( \tau_{k}\left( t \right)+\rho_{k}+\mu_{i}(t) \right)U_{ij}^{k}\left( t \right); if k=2,3,4 \#\left( 3.2 \right) \end{aligned}$$

1. The number of diagnosed individuals that are not on treatment (D) are modeled according to:

$$\begin{aligned} \frac{dD_{ij}^{k}\left( t \right)}{dt}={\tau_{k}\left( t \right)U}_{ij}^{k}\left( t \right) -{(\gamma_{k}\left( t \right)+\rho}_{k}+\mu_{i}(t))D_{ij}^{k}\left( t \right); if k=1 \#\left( 4.1 \right) \end{aligned}$$

$$\begin{aligned} \frac{dD_{ij}^{k}\left( t \right)}{dt}={\tau_{k}\left( t \right)U}_{ij}^{k}\left( t \right)+\rho_{\left( k-1 \right)}D_{ij}^{k-1}\left( t \right) -{(\gamma_{k}\left( t \right)+\rho}_{k}+\mu_{i}(t))D_{ij}^{k}\left( t \right); if k=2,3,4 \#\left( 4.2 \right) \end{aligned}$$

1. The number of diagnosed individuals on treatment (A) are modeled according to:

$$\begin{aligned} \frac{dA_{ij}^{k}\left( t \right)}{dt}=\gamma_{k}\left( t \right)D_{ij}^{k}\left( t \right) -\mu_{i}(t)A_{ij}^{k}\left( t \right) \#\left( 5 \right) \end{aligned}$$

# Model demography

#### Population size and growth

## Québec

We used provincial population estimates from Statistics Canada^3^ to model the population size of Québec. As the province experience periods of growth over 1975–2021, we set the initial model population to the 1975 population size by sex and age group (Table S1) and applied a time-varying growth factor calculated from the overall and sex-stratified population data over 5-year periods assuming an exponential distribution (Table S2).

**Table S1**: Population size of Québec in 1975 by sex and age^3^

| **Age group (years)** | **Population size** | | |
| --- | --- | --- | --- |
|  | **Male** | **Female** | **Total** |
| 15 – 19 | 342,560 | 332,085 | 674,645 |
| 20 – 24 | 315,832 | 310,638 | 626,470 |
| 25 – 29 | 286,538 | 280,007 | 566,545 |
| 30 – 34 | 238,742 | 231,951 | 470,693 |
| 35 – 39 | 188,075 | 183,920 | 371,995 |
| 40 – 44 | 181,940 | 180,788 | 362,728 |
| 45 – 49 | 174,267 | 178,320 | 352,587 |
| 50 – 54 | 158,232 | 167,433 | 325,665 |
| 55 – 59 | 127,130 | 137,522 | 264,652 |
| 60 – 64 | 111,039 | 123,272 | 234,311 |
| $\geq$65 | 199,612 | 263,468 | 463,080 |
| Total (15 and over) | 2,326,861 | 2,394,636 | 4,721,497 |

**Table S2:** Population size and exponential growth factor of Québec over 1975–2020 by sex^3^

| **Population size** | | | | **Growth factor** | | | |
| --- | --- | --- | --- | --- | --- | --- | --- |
| **Year** | **Male** | **Female** | **Total** | **Year** | **Male** | **Female** | **Total** |
| 1975 | 3,150,965 | 3,179,338 | 6,330,303 | 1975 | - | - | - |
| 1976 | 3,182,546 | 3,214,215 | 6,396,761 | 1976 – 1980 | 0.003 | 0.006 | 0.005 |
| 1981 | 3,237,077 | 3,310,130 | 6,547,207 | 1981 – 1985 | 0.004 | 0.005 | 0.005 |
| 1986 | 3,308,452 | 3,399,718 | 6,708,170 | 1986 – 1990 | 0.010 | 0.011 | 0.010 |
| 1991 | 3,481,065 | 3,586,331 | 7,067,396 | 1991 – 1995 | 0.005 | 0.005 | 0.005 |
| 1996 | 3,568,418 | 3,678,479 | 7,246,897 | 1996 – 2000 | 0.004 | 0.004 | 0.004 |
| 2001 | 3,647,738 | 3,748,718 | 7,396,456 | 2001 – 2005 | 0.007 | 0.006 | 0.006 |
| 2006 | 3,777,953 | 3,854,013 | 7,631,966 | 2006 – 2010 | 0.010 | 0.009 | 0.010 |
| 2011 | 3,971,988 | 4,033,102 | 8,005,090 | 2011 – 2015 | 0.006 | 0.005 | 0.005 |
| 2016 | 4,095,582 | 4,130,368 | 8,225,950 | 2016 – 2020 | 0.010 | 0.008 | 0.009 |
| 2021 | 4,304,047 | 4,300,448 | 8,604,495 | - | - | - | - |

## Montréal

To model the population size of Montréal, we used Canadian census data (1976–1991)^4-7^ and Institut de la statistique du Québec (ISQ) population size estimates (1996–2021)^8^ of the Montréal administrative region (i.e. the island of Montréal). From 1975-1986, this region experienced a population decline, after which the population again began to increase. As there remains uncertainty in the demographics of those who migrated out of Montréal during that time, like another mathematical model of HIV transmission among Montréal MSM developed by Milwid et al.^9^, we assumed the Montréal population size was constant over 1975–1986. We used the population size of males and females per age group in 1986 to set Montréal's initial population in the model (Table S3). Subsequent growth of the Montréal population over 1986–2021 was modeled using a time-varying growth factor calculated from the overall and sex-stratified population data over 5-year periods, assuming an exponential distribution (Table S4).

**Table S3:** Population size of the administrative region of Montréal in 1986 by sex and age^6^

| **Age group (years)**^*^ | **Population size** | | |
| --- | --- | --- | --- |
|  | **Male** | **Female** | **Total** |
| 15 – 19 | 58,765 | 57,315 | 116,080 |
| 20 – 24 | 85,020 | 87,110 | 172,130 |
| 25 – 29 | 81,135 | 81,838 | 162,973 |
| 30 – 34 | 81,135 | 81,838 | 162,973 |
| 35 – 39 | 58,473 | 62,828 | 121,300 |
| 40 – 44 | 58,473 | 62,828 | 121,300 |
| 45 – 49 | 47,085 | 51,993 | 99,078 |
| 50 – 54 | 47,085 | 51,993 | 99,078 |
| 55 – 59 | 44,988 | 53,343 | 98,330 |
| 60 – 64 | 44,988 | 53,343 | 98,330 |
| $\geq$65 | 83,855 | 140,495 | 224,350 |
| Total (15 and over) | 691,000 | 784,920 | 1,475,920 |

^*^Available data summarized using the following age groups: 15–19, 20–24, 25–34, 45–54, 55–59, 60–64, and $\geq$65. To model 5-year age groups, it was assumed that 50% of each 10-year age group belonged to each of the corresponding 5-year age groups.

**Table S4:** Population size and exponential growth factor of the Montréal administrative region over 1986–2020 by sex^3^

| **Population size** | | | | **Growth factor** | | | |
| --- | --- | --- | --- | --- | --- | --- | --- |
| **Year** | **Male** | **Female** | **Total** | **Year** | **Male** | **Female** | **Total** |
| 1986 | 832,155 | 920,430 | 1,752,585 | 1986 – 1990 | 0.003 | 0.002 | 0.003 |
| 1991 | 846,305 | 929,565 | 1,775,870 | 1991 – 1995 | 0.004 | 0.001 | 0.002 |
| 1996 | 863,685 | 934,227 | 1,797,912 | 1996 – 2000 | 0.007 | 0.004 | 0.006 |
| 2001 | 895,533 | 954,613 | 1,850,146 | 2001 – 2005 | 0.004 | 0.001 | 0.002 |
| 2006 | 915,017 | 958,008 | 1,873,025 | 2006 – 2010 | 0.005 | 0.004 | 0.004 |
| 2011 | 938,707 | 975,049 | 1,913,756 | 2011 – 2015 | 0.006 | 0.004 | 0.005 |
| 2016 | 965,396 | 993,618 | 1,959,014 | 2016 – 2020 | 0.008 | 0.005 | 0.007 |
| 2021 | 1,005,485 | 1,020,443 | 2,025,928 | - | - | - | - |

#### Exposure category population sizes

Exposure category population size estimates were obtained from various population-based health surveys and are summarized in Table S5. The main survey, *l’Enquête Québécoise sur la santé de la population* (EQSP)^10, 11^, was implemented in 2008 and 2014–2015 by the ISQ and captured behavioural information on sexual partnerships and drug use for all of Québec, as well as by region. Supplemental sources included *l’Enquête sur les attitudes et comportements reliés au VIH/sida dans la population générale Québécoise* (EHAQ; 1995–1997)^44^ and summarized information from multiple Canadian Health Monitor Surveys (CHMS; 1994, 1995–1996, 1997), as reported by Archibald et al.^12^

**Table S5:** Estimates of the MSM and PWID population sizes in Québec and Montréal by sex

| **Location** | **Year** | **Male** | **Female** | **Overall** | **Source** |
| --- | --- | --- | --- | --- | --- |
| *MSM: % of male population aged* $\geq$*15 years that had sex with another man in the past 12 months* | | | | | |
| Québec | 1995-1997 | 3.4%^*^ | - | - | EHAQ^44^ |
| Québec | 2008 | 2.5% [2.1% had sex only with men; 0.4% had sex with men and women] | - | - | EQSP^11^ |
| Québec | 2014-2015 | 3.4% [2.7% (2.3 – 3.2%)^†^ had sex only with men; 0.7% (0.5 – 0.9%)^†^ had sex with men and women] | - | - | EQSP^10^ |
| Montréal | 1994-1997 | 1.4 – 3.9% [interval mid-point: 2.65%] | - | - | CHMS^12^ |
| Montréal | 2008 | 5.2% [4.7% (2.9 – 6.7%)^‡^ had sex only with men; 0.5% (0.0 – 1.1%)^‡^ had sex with men and women] | - | - | EQSP^11^ |
| Montréal | 2014-2015 | 6.4% [5.6% (4.5 – 6.8%)^‡^ had sex only with men; 0.8% (0.5 – 1.4%)^‡^ had sex with men and women] | - | - | EQSP^10^ |
| *PWID: % of population aged* $\geq$*15 years that ever used injection drugs* | | | | | |
| Québec | 1995-1997 | 0.8%^*^ | 0.2%^*^ | 0.5%^*^ | EHAQ^44^ |
| Québec | 2008 | 0.7% | 0.2% | 0.5% | EQSP^11^ |
| Québec | 2014-2015 | 1.1% (0.9 – 1.4%)^†^ | 0.5% (0.4 – 0.7%)^†^ | 0.8% (0.7 – 1.0%)^†^ | EQSP^10^ |
| Montréal | 1994-1997 | Not available | Not available | 0.6 – 1.8% [interval mid-point: 1.2%] | CHMS^12^ |
| Montréal | 2008 | 0.9% (0.2 – 1.8%)^‡^ | Not available | 0.6% (0.20 – 1.1%)^‡^ | EQSP^11^ |
| Montréal | 2014-2015 | 1.8% (1.3 – 2.5%)^‡^ | 0.9% (0.5 – 1.3%)^†^ | 1.3% (1.0 – 1.7%)^‡^ | EQSP^10^ |

Abbreviations: *l’Enquête sur les attitudes et comportements reliés au VIH/sida dans la population générale Québécoise* (EHAQ); *l’Enquête Québécoise sur la santé de la population* (EQSP); Canadian Health Monitor Surveys (CHMS).

^*^% of population aged 15-64 years

^†^99% confidence interval

^‡^95% confidence interval

## Men who have sex with men

Over 2014-2015, an estimated 3.4% of sexually active men aged $\geq$15 years had sex with another man in the past 12 months in Québec, with 2.7% (99% CI: 2.3-3.2%) having had sex exclusively with men and 0.7% (99% CI: 0.5-0.9%) having had sex with both men and women^10^. In Montréal, 6.4% of sexually active men aged 15 years and older were estimated to have had sex with another man in the past 12 months, with 5.6% (95% CI: 4.5-6.8%) having had sex only with men and 0.8% (95% CI: 0.5-1.4%) having had sex with both men and women^10^. These proportions were similar to that estimated in the 2008 EQSP^11^, as well as the other reports identified^12, 13^. Therefore, we used the 2014-2015 EQSP estimates to model the MSM populations of Québec and Montréal, assuming the proportion of MSM remained constant in time and that all MSM enter the model at 15 years of age.

## People who use injection drugs

##### Lifetime injection drug use

The provincial HIV surveillance system attributes an HIV diagnosis to injection drug use (IDU) if the diagnosed individual reports having ever used injection drugs in their lifetime. We correspondingly modeled the population aged $\geq$15 years who ever used injection drugs over time, thereby, including both active and former PWID in the model.

Few estimates of lifetime IDU for Québec and Montréal were identified, with the earliest in both areas being captured in the mid-1990s (Table S5). In Québec, the overall and sex-stratified estimates remained consistent over time, with the most recent estimates from the 2014-2015 EQSP suggesting 1.1% (99% CI: 0.9-1.4%) of males and 0.5% (99% CI: 0.4-0.7%) of females aged $\geq$15 years in Québec ever used injection drugs^10^. In Montréal, few sex-stratified estimates were available prior to the 2014-2015 EQSP, where an estimated 1.8% (95% CI: 1.3 – 2.5%) of males and 0.9% (95% CI: 0.5 – 1.3%) of females aged $\geq$15 years reported lifetime IDU^10^. Comparing the overall prevalence of lifetime IDU among those aged $\geq$15 years in Montréal, however, indicated that the proportion remained stable over time.

Given the paucity of data prior to 1994-1995, as well as sex-stratified estimates for Montréal, and the similarity of the identified estimates over time, we assumed the proportion of lifetime IDU overall and among males and females in Québec and Montréal remained constant at the values determined in 2014-2015^10^. An overall and sex-specific probability distribution for the age of IDU initiation was used to allow PWID to enter the model at each age group. This distribution was informed by data from the SurvUDI network (see Section 3), which indicated that over 2005-2008, the mean and standard deviation (SD) of reported age of IDU initiation among active male and female PWID in Québec and the city of Ottawa, Ontario was 23.5 years (SD: 8.5) and 22.6 years (SD: 8.6), respectively^14^. Assuming the number of individuals initiating IDU at each age follows a zero-truncated negative binomial distribution (equations 6.1–6.3), we used the mean and SD values described above to determine the probability of initiation per age group overall and among males and females (Table S6). We assumed the same probability distribution also applied to PWID in Montréal.

$$\begin{aligned} A{ge at IDU initiation}_{male}\sim ZTNB\left( mean=23.5 , size=\frac{{23.5}^{2}}{{8.5}^{2}- {23.5}^{2}} \right) \#(6.1) \end{aligned}$$

$$\begin{aligned} A{ge at IDU initiation}_{female}\sim ZTNB\left( mean=22.6 , size=\frac{{22.6}^{2}}{{8.6}^{2}- {22.6}^{2}} \right) \#(6.2) \end{aligned}$$

$$\begin{aligned} A{ge at IDU initiation}_{overall}\sim ZTNB\left( mean=23.3, size=\frac{{23.3}^{2}}{{8.5}^{2}- {23.3}^{2}} \right) \#(6.3) \end{aligned}$$

**Table S6**: Probability of injection drug use initiation by sex and age group for Québec and Montréal, assuming a zero-truncated negative binomial distribution

| **Age group (years)** | **Probability** | | |
| --- | --- | --- | --- |
|  | **Male** | **Female** | **Overall** |
| < 15 | 0.137 | 0.172 | 0.143 |
| 15 – 19 | 0.211 | 0.223 | 0.214 |
| 20 – 24 | 0.239 | 0.232 | 0.238 |
| 25 – 29 | 0.191 | 0.175 | 0.188 |
| 30 – 34 | 0.119 | 0.105 | 0.116 |
| 35 – 39 | 0.061 | 0.054 | 0.059 |
| 40 – 44 | 0.027 | 0.024 | 0.026 |
| 45 – 49 | 0.011 | 0.010 | 0.010 |
| 50 – 54 | 0.004 | 0.004 | 0.004 |
| 55 – 59 | 0.001 | 0.001 | 0.001 |
| 60 – 64 | 0.000 | 0.000 | 0.000 |
| $\geq$65 | 0.000 | 0.000 | 0.000 |

##### Active injection drug use

While the entire model captures lifetime IDU, HIV acquisition and IDU-related mortality can only occur among the proportion actively injecting. Available estimates of the active PWID population sizes in Québec and Montréal are in Table S7.

In Montréal, two data points from 1996 and 2009-2010 are available and indicate the number of active PWID in the city declined from 11,680 to 3,910 people over that period^15, 16^. In the model, we assumed the number of active PWID in Montréal remained constant at 11,680 people over 1975-1996, declined linearly over 1996-2009 according to annual estimates obtained by linear interpolation, and afterward remained constant at 3,910 people.

In Québec, the earliest estimate from the 1995-1997 EHAQ survey^44^ cannot be considered reliable due to the small number of reported active PWID in the sample. Therefore, only data from more recent years (2011-2016) can inform the population of active PWID in the province. To approximate the Québec active PWID population size before 2011, we triangulated the Montréal data to determine the proportion of lifetime PWID active in 1996 (59.7%). We assumed the same proportion applied to the 1996 population of lifetime PWID in Québec, resulting in 28,017 active PWID. As done in Montréal, we assumed the active PWID population size in Québec was constant at 28,017 people over 1975-1996 and performed linear interpolation to approximate the population decline to 11,300 people over 1996-2011^17^, following which we used the available data points. Finally, the 2016 estimate of 14,900 people was assumed constant over 2016-2020.

In Montréal and Québec, we assumed the proportion of the active PWID population that were male and female was constant over time at 0.721 and 0.279, respectively, consistent with the 2009-2010 sex-stratified Montréal estimate. Figure S1 displays the assumed population size of active PWID in Montréal and Québec over time.

**Table S7**: Estimated size of the active PWID population aged 15-64 years in Montréal and Québec by sex

| **Location** | **Year** | **Male** | **Female** | **Overall** | **Source** |
| --- | --- | --- | --- | --- | --- |
| Québec | 1995-1997 | 0.1% | 0.06% | 0.1% | EHAQ^44^ |
| Québec | 2011 | Not available | Not available | 11,300 (10,000-12,500)^*^ | Jacka^17^ |
| Québec | 2012 | Not available | Not available | 12,300 (10,900-13,700)^*^ | Jacka^17^ |
| Québec | 2013 | Not available | Not available | 13,900 (12,300-15,500)^*^ | Jacka^17^ |
| Québec | 2014 | Not available | Not available | 13,700 (12,200-15,300)^*^ | Jacka^17^ |
| Québec | 2015 | Not available | Not available | 14,700 (13,000-16,400)^*^ | Jacka^17^ |
| Québec | 2016 | Not available | Not available | 14,900 (13,200-16,600)^*^ | Jacka^17^ |
| Montréal | 1996 | Not available | Not available | 11,680 (8,640-16,460)^†^ | Remis et al.^15^, as reported by Archibald et al.^12^ |
| Montréal | 2009-2010 | 2,820 (2,300-3,540)^†^ | 1,090 (810-1,500)^†^ | 3,910 (3,180 – 4,900)^†^ | Leclerc^16^ |

^*^Range of the estimated population size

^†^95% confidence interval

| 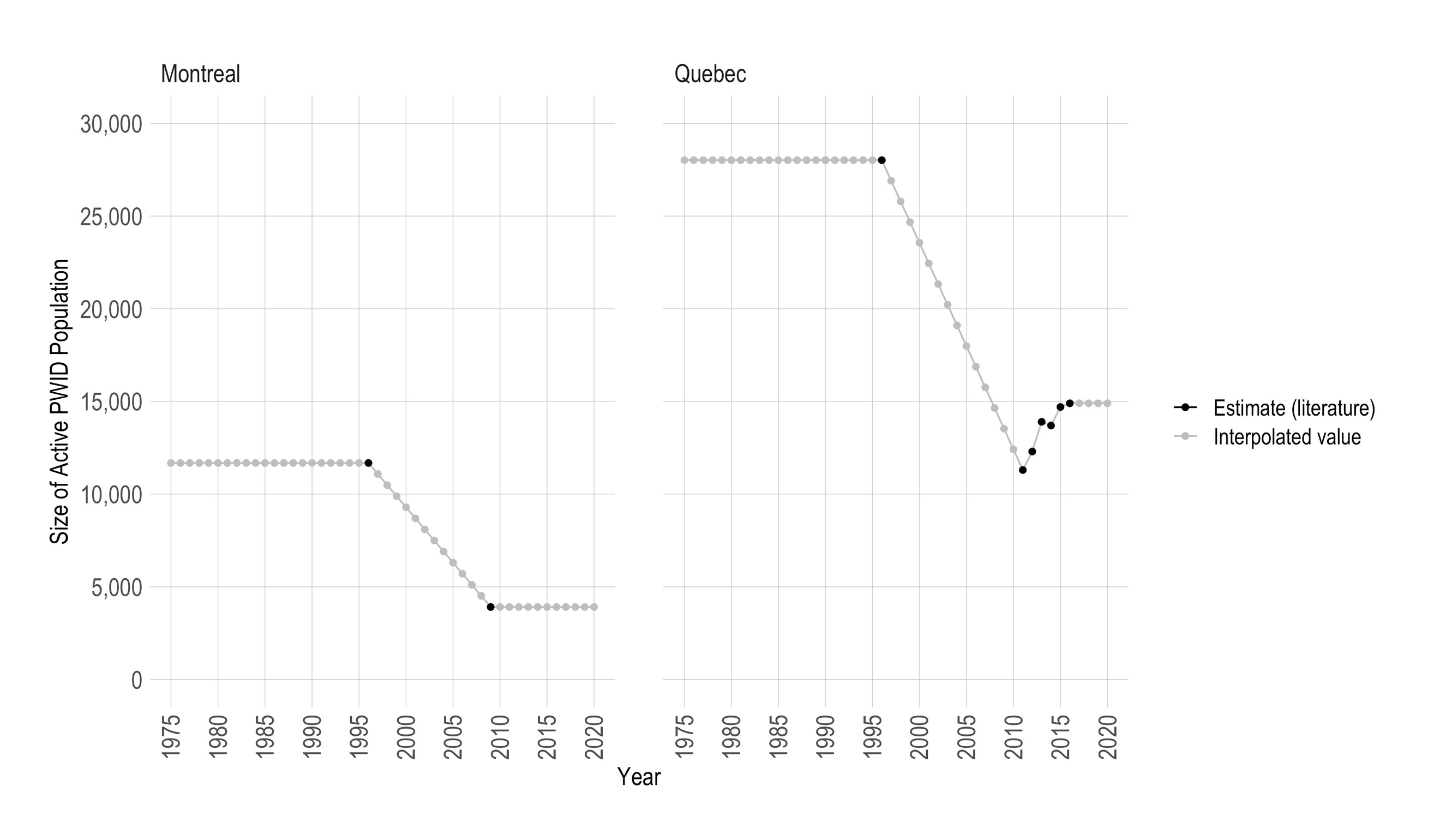 |
| --- |
| **Figure S1.** Modeled active PWID population size aged $\geq$15 in Montréal and Québec over 1975-2020 |

## Summary

Table S8 summarizes the proportion of the population assumed to belong to each exposure category and used to establish the model populations.

**Table S8:** Proportion of the population $\geq$15 years assumed to belong to each exposure category over time by sex in Québec and Montréal

| **Exposure category** | **Sex** | **Estimated proportion** | | **Source** |
| --- | --- | --- | --- | --- |
|  |  | **Québec** | **Montréal** |  |
| MSM | Male | 3.4% | 6.4% | EQSP, 2014-2015^10^ |
| PWID | Male | 1.1% | 1.8% |  |
|  | Female | 0.5% | 0.9% |  |
|  | Overall | 0.8% | 1.3% |  |

#### Mortality

Individuals can exit the model due to all-cause or AIDS-related mortality. Mortality data from vital statistics^18^ and population data from Statistics Canada^3^ were used to calculate annual age-specific all-cause mortality rates for Québec (Figure S2). Among PWID, the all-cause mortality rates additionally included a weighted IDU-related mortality rate to reflect the increased mortality observed among active PWID^19^ (see *IDU-related mortality* below). PLHIV can also experience AIDS-related mortality (Table S9).

| 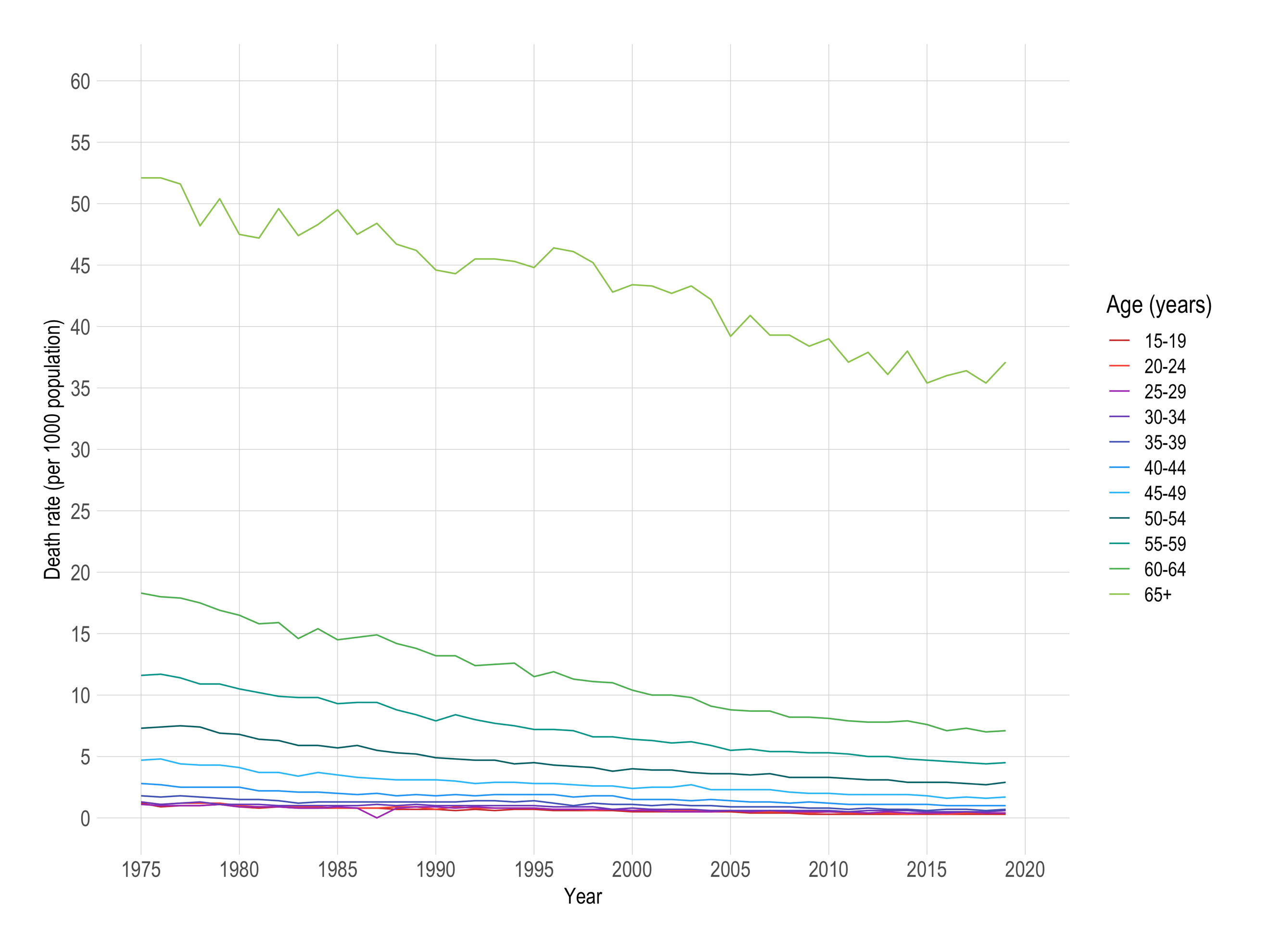 |
| --- |
| **Figure S2.** All cause-mortality rates for Québec by age group |

## IDU-related mortality

Compared to those who do not use injection drugs, active PWID have a higher risk of death^19^. A previous systematic review determined that active PWID experience a crude all-cause mortality rate of 2.35 deaths per 100 person–years (95% CI: 2.12–2.58), with drug overdose being a leading cause of death^19^. Since our model captures both active and former PWID, we weighted this rate by the proportion of active PWID in the model population at each iteration.

# Model parameterization

**Table S9**: Model parameter values and prior distributions used to estimate HIV incidence among MSM, PWID, and heterosexual populations in Montréal and Québec.

| **Parameter** | **Symbol**^*^ | **Unit** | **Value, range, or prior distribution** | **Source** |
| --- | --- | --- | --- | --- |
| *Demography* | | | | |
| Population growth rate | $\zeta_{j}(t)$ | year^-1^ | See Tables S2 and S4 | Census^4-7^ and population estimates^3, 8^ |
| Background mortality rate | $\mu_{i}(t)$ | year^-1^ | See Figure S2 | Vital statistics^18^ and population estimates^3^ |
| IDU-related mortality rate | $\mu_{IDU}(t)$ | 1000 person-years^-1^ | 23.5^†^ | Mathers^19^ |
| Population recruitment size | $\alpha_{ij}(t)$ | people | $\theta_{j}(t-1)$ + $\zeta_{j}\left( t \right)*N_{j}(t-1)$, where $\theta_{j}\left( t-1 \right)$ and$N_{j}(t-1)$ are the total number of deaths and the population size in the model at time$t-1$, respectively. | See Section 2 |
| Proportion of HIV-susceptible population at-risk due to sexual activity or active IDU | $\delta_{j}(t)$ | - | MSM: 1.0  PWID: see Figure S1 | See Section 2 |
| *Natural history* | | | | |
| HIV incidence rate^‡^ | $\lambda_{ij}\left( t \right)$ | year^-1^ | When estimating incidence overall:  ${b_{j}}_{1}=0$  ${b_{j}}_{2,3}\sim{logit}^{-1}\left( N\left( 1, 1.5 \right) \right)*0.25$  ${b_{j}}_{n}\sim{logit}^{-1}\left( N\left( -1, 1.5 \right) \right)*0.15,$ for $n\in$ [4, *m* + 3]  When estimating incidence by age:  ${b_{j}}_{2,3}\sim{logit}^{-1}\left( N\left( 0, 1.55 \right) \right)*\left( b_{2,3}-a_{2,3} \right)+a_{2,3}$  ${b_{j}}_{n}\sim{logit}^{-1}\left( N\left( 0, 1.55 \right) \right)*\left( b_{n}-a_{n} \right)+a_{n},$ for $n\in$ [4, *m* + 3]  ${u_{ij}}_{n}\sim N\left( 0, 0.2 \right)$ | Calibration |
| Duration of primary infection | ${1/\rho}_{p}$ | year | 0.242 | Hollingsworth^20^ |
| Disease progression rate (in absence of ART)^§^ | $\rho_{k}$ | year^-1^ | $\rho_{1}$ = 1/3.32  $\rho_{2}$ = 1/2.70 $\rho_{3}$ = 1/5.50 $\rho_{4}$= 1/5.06 | Cori^21^ |
| Proportion of primary infection subsequently allocated to each disease stage | $f_{k}$ | N/A | $f_{1}$ = 0.76  $f_{2}$ = 0.19  $f_{3}$ = 0.05  $f_{4}$ = 0.00 | Cori^21^ |
| *Diagnostic and treatment rates* | | | | |
| Testing rates^¶^ | $\tau_{jk}\left( t \right)$ | year^-1^ | $s_{k = S,1,2,3} \sim{logit}^{-1}\left( N\left( 0, 1.55 \right) \right)*\left( 0.5-0.05 \right)+0.05$  $s_{k = 4} \sim{logit}^{-1}\left( N\left( 0, 1.55 \right) \right)*\left( 0.75-0.25 \right)+0.25$  $c_{k =S,1,2,3}= 2000$  $c_{k = 4}= 1985$  $l_{S}=1.65$ for MSM  $l_{S} \sim{logit}^{-1}\left( N\left( 0, 1.55 \right) \right)*\left( 2-0.05 \right)+0.05$ for PWID  $l_{k=1,2,3} \sim{logit}^{-1}\left( N\left( 0, 1.55 \right) \right)*\left( 2-0.05 \right)+0.05$  $l_{k=4}=8$ | Calibration |
| Proportion of AIDS diagnoses reported | $\phi_{A}(t)$ | - | MSM: 0.85 for $t\in$ [1975, 1996], 0.75 for $t$ $\in$ [1997, 2002]  PWID: 0.75 for *t* $\in$ [1975, 2002] | Remis^22^, assumption |
| Proportion of new HIV diagnoses reported^#^ | $\phi_{H}(t)$ | - | $\sim{logit}^{-1}\left( N\left( 0, 1.55 \right) \right)*\left( 1-0.6 \right)+0.6$ for *t* $\in$ [2003, 2011]  $\sim{logit}^{-1}\left( N\left( 0, 1.55 \right) \right)*\left( 1-0.6 \right)+0.6$ for *t* $\geq$ 2012 | Calibration |
| ART treatment rate | $\gamma_{k}\left( t \right)$ | year^-1^ | MSM:  $\gamma_{3,4}\left( t \right)= \left\{ \begin{aligned} 0, t<1996 \\ \frac{1}{2.06}, t\in\left[ 1996, 2003 \right) \\ \frac{1}{0.40}, t\in\left[ 2003, 2012 \right) \\ \frac{1}{0.08}, t\geq2013 \end{aligned} \right.$  $\gamma_{2}\left( t \right)= \left\{ \begin{aligned} 0, t<2013 \\ \frac{1}{0.08}, t\geq2013 \end{aligned} \right.$  $\gamma_{1}\left( t \right)= \left\{ \begin{aligned} 0, t<2015 \\ \frac{1}{0.08}, t\geq2015 \end{aligned} \right.$  PWID:  $\gamma_{3,4}\left( t \right)= \left\{ \begin{aligned} 0, t<1996 \\ \frac{1}{4.12}, t\in\left[ 1996, 2003 \right) \\ \frac{1}{0.80}, t\in\left[ 2003, 2012 \right) \\ \frac{1}{0.16}, t\geq2013 \end{aligned} \right.$  $\gamma_{2}\left( t \right)= \left\{ \begin{aligned} 0, t<2013 \\ \frac{1}{0.16}, t\geq2013 \end{aligned} \right.$  $\gamma_{1}\left( t \right)= \left\{ \begin{aligned} 0, t<2015 \\ \frac{1}{0.16}, t\geq2015 \end{aligned} \right.$ | Assumption^**^ |

Abbreviations: injection drug use (IDU); men who have sex with men (MSM); people who inject drugs (PWID); antiretroviral therapy (ART).

^*^The symbol *t* indicates time. Index *i* indicates age group (15-19, 20-24, 25-29, 30-34, 35-39, 40-44, 45-49, 50-54, 55-59, 60-64, 65+), *j* indicates sex (male, female), and *k* indicates disease stage (S=susceptible; 1=CD4 >500 cells/µL; 2=CD4 350-500 cells/µL; 3=CD4 200-349 cells/µL; and 4=CD4 <200 cells/µL).

^†^Only applicable to the PWID model and weighted by the proportion of active PWID in the model population over time. See Section 2 for more detail.

^‡^Modeled by a cubic M-spline with $m$ internal knots and $n=m+3$ coefficients${\beta_{ij}}_{n}=e^{log({b_{j}}_{n})+{u_{ij}}_{n}}\geq0$, where ${u_{ij}}_{n}=0$ when estimating incidence overall, rather than varied by age (see Section 3 for more detail). To estimate incidence by age, an empirical Bayes approach was taken and the priors for the coefficients${\beta_{ij}}_{n}$ were narrowed to that of the posterior range $\left( a_{n}, b_{n} \right)$ of the coefficients when estimated overall.

^§^$\rho_{4}$ corresponds to the AIDS-related mortality rate.

^¶^Modeled by logistic growth curves with scale (*s*), midpoint (*c*), and limiting (*l*) parameters such that the testing rate at time *t* is calculated as $\tau_{jk}\left( t \right)=\frac{l_{k}}{1 + e^{-s_{k} * \left( t - c_{k} \right)}}$. For MSM, $l_{S}$ was estimated from the RDS-adjusted annual testing rate of the Engage Study (see Section 3). All other fixed values or distribution parameters were assumed.

^#^For simplicity, we assumed reporting was uncorrelated with age or CD4 count.

^**^For MSM, the time-varying ART initiation rates for $\gamma_{3,4}\left( t \right)$over $t\in\left[ 1996, 2003 \right)$ and $t\in\left[ 2003, 2012 \right)$were calculated from the Argus I and Engage studies (see Section 3) and were assumed from 2013 onward. For PWID, we assumed the time to ART initiation was twice that among MSM.

#### Data sources

Peer-reviewed literature and data from three local studies were used to inform the model parameters. Key features of these studies and the parameters they informed are summarized in Table S10. Sources for all other input parameters are provided in Table S9.

**Table S10**: Local data sources used to inform MSM and PWID model parameters.

| **Study** | **Population** | **Location** | **Year (N)** | **Design** | **Sample type** | **Parameters** |
| --- | --- | --- | --- | --- | --- | --- |
| Argus^23-25^ | MSM | Montréal | 2005-2007 (1,957);  2008-2009 (1,873) | Repeated cross-sectional | Convenience (modified time-location sampling) | ART initiation rate |
| Engage^26^ | MSM | Montréal | 2017-2018 (1,179) | Cohort | Respondent driven sampling | HIV testing rate, ART initiation rate |
| SurvUDI^27, 28^ | PWID | Québec and Ottawa, Ontario | 1995 – present (15,200) | Repeated cross-sectional | Convenience | Model entry (age at IDU initiation) |

Abbreviations: men who have sex with men (MSM); antiretroviral treatment (ART); people who use injection drugs (PWID); injection drug use (IDU).

## Men who have sex with men

Data from two studies of Montréal MSM were used to inform the model parameters. The first, Argus, was a repeated cross-sectional survey with two cycles over 2005-2007 (Argus I, N=1,957)^23, 24^ and 2008-2009 (Argus II, N=1,873)^25^. At each cycle, MSM aged ≥18 years were recruited into the study at various community venues using a modified time-location sampling method. The second study, Engage, is an ongoing prospective cohort study of MSM aged ≥16 years in Montréal, Toronto, and Vancouver. For these purposes, we used the baseline data of the Montréal participants (N=1,179), recruited over 2017-2018^26^ by respondent-driven sampling (RDS), constituting a more representative sample of Montréal MSM. To account for the convenience sampling used in Argus, the data were standardized to the Engage study based on age and ethnicity (Canadian-born vs. elsewhere).

Together, these surveys informed the HIV testing rate among HIV-negative MSM and the time-varying ART initiation rates among PLHIV. Specifically, the annual testing rate among HIV-negative participants was calculated and adjusted using RDS-II weights to account for the sampling strategy. The resulting estimate was assumed to be the limiting value of the logistic growth curve used to model the testing rate among those susceptible to HIV acquisition ($l_{S}$). To determine the ART initiation rates ($\gamma_{k}\left( t \right)$), we used Poisson regression to estimate the mean duration from HIV diagnosis to first use of ART among men aware they were living with HIV in the Argus I (those diagnosed over 1996-2005) and Engage (those diagnosed after 2005) studies. We stratified the estimate by period (1997-2003 and 2004-2013) to allow the parameter value to time-vary. We again adjusted for the sampling method by applying RDS-II weights to the Engage participants and standardization weights to those from Argus I.

## People who inject drugs

Among PWID, we used reported information from the SurvUDI network to inform the age at IDU initiation (as described above in *Model demography*). SurvUDI is an ongoing, repeated cross-sectional surveillance study of active PWID that has been conducted annually since 1995 across regions of Québec and the city of Ottawa, Ontario^27, 28^. By the end of March 2018, N=15,200 unique PWID aged ≥14 years that used drugs in the past six months were recruited into the study from various sites frequented by active PWID, including needle exchange program sites (constituting around 90% of the recruitment), rehabilitation centers, detention centers and shelters^28^. Individuals could participate in the study multiple times and at any location, with a minimum period of at least six months between visits.

#### Incidence spline

The time-varying HIV incidence rate was flexibly modeled over 1975–2020 by a cubic M-spline^2, 29^, a continuous function of non-negative piecewise polynomials joining together at $m$ internal knot points $\upsilon_{1}<\ldots<\upsilon_{m}$. Let *t* indicate time, *i* age, *j* sex, *p* the polynomial degree (where *p*=3 corresponds to cubic polynomials), ${\beta_{ij}}_{n}$ the spline coefficients, and M(t) the M-spline basis^29^. The HIV incidence curve over time, $\lambda_{ij}\left( t \right),$ is as formulated in equation 7.0:

$$\begin{aligned} \lambda_{ij}\left( t \right)= \sum_{n=1}^{m+p} {\beta_{ij}}_{n}*M\left( t \right)\#\left( 7.0 \right), \end{aligned}$$

where ${\beta_{ij}}_{n}=e^{log({b_{j}}_{n})+{u_{ij}}_{n}}$ and ${u_{ij}}_{n}=0$ when modeling incidence overall, rather than by age.

The R package *splines2^30^* was used to formulate M(t) in our model. The first spline coefficient ${\beta_{ij}}_{1}$was set to 0 so that incidence would begin at 0 in 1975$.$ The remaining coefficients, which are constrained to be positive, were obtained by estimating $b_{i}$ and $u_{ij}$ (when estimating incidence by age) in model calibration on the log-scale. The number and location of knots was varied to determine the best fitting incidence curve. Three scenarios of knot placements were explored (Table S11): 1) equidistant knots; 2) knots focused earlier in the epidemic, where increased flexibility might be needed; and 3) knots focused where more data is available. The model resulting in the lowest Watanabe–Akaike information criterion (WAIC) or leave-one-out information criteria (LOOIC) was selected as the final model.

**Table S11**: Incidence M-spline scenarios for knot placement.

| **N knots** | **Knot locations** | | |
| --- | --- | --- | --- |
|  | **1. Equally spaced** | **2. Focused where increased flexibility needed** | **3. Focused where more available data** |
| 3 | 1986.3, 1997.5, 2008.8 | 1985, 1996, 2000 | 1997, 2003, 2007 |
| 4 | 1984, 1993, 2002, 2011 | 1985, 1990, 1996, 2000 | 1997, 2003, 2007, 2010 |
| 5 | 1982.5, 1990.0, 1997.5, 2005.0, 2012.5 | 1980, 1985, 1990, 1996, 2000 | 1997, 2003, 2007, 2010, 2014 |
| 6 | 1981.4, 1987.9, 1994.3, 2000.7, 2007.1, 2013.6 | 1980, 1985, 1990, 1996, 2000, 2007 | 1997, 2003, 2007, 2010, 2014, 2018 |
| 7 | 1980.6, 1986.3, 1991.9, 1997.5, 2003.1, 2008.8, 2014.4 | 1980, 1985, 1990, 1996, 2000, 2007, 2014 | 1985, 1997, 2003, 2007, 2010, 2014, 2018 |

# Model calibration and cross-validation

#### Calibration and cross-validation outcomes

Model calibration is performed to estimate values of the unknown parameters. This involves comparing the model outcomes to independent, empirical data points to obtain the set of parameter values that best fit the data and match the epidemic trends^33, 34^. Table S12 details the outcomes used for model calibration and cross-validation. The main calibration data source was provincial surveillance data from *l'Institut national de santé publique du Québec* (INSPQ). At the beginning of the epidemic, only AIDS cases were collected by Québec’s surveillance program^35, 36^. While the reporting of AIDS cases continued until 2002, the availability of ART in 1997 led to a subsequent reduction in the number of PLHIV advancing to the AIDS stage and a greater uncertainty in the completeness of reporting after that time. Part way through 2002, HIV came under surveillance in Québec, becoming reportable by the provincial laboratories responsible for carrying out tests, along with some relevant epidemiological data^36, 37^. Correspondingly, the INSPQ data utilized includes the annual number of AIDS cases over 1979-1998 and the annual number of HIV diagnoses from 2003-2020. The AIDS data is stratified by exposure category and sex. The HIV diagnosis data is stratified by exposure category, sex, age, and self-reported time since last negative HIV test (2003-2020). More recently, HIV diagnoses are also stratified by CD4 cell count at new diagnosis (2013-2020).

Data from the Argus surveys and the Engage study were used as cross-validation outcomes for Montréal MSM. Again, Argus survey estimates were standardized to the Engage study. Among PWID, there were no comparable data for cross-validation (SurvUDI estimates do not apply due to differences in the population, i.e. active vs. lifetime PWID).

**Table S12:** Outcomes used for model calibration and cross-validation.

| **Outcomes** | **Time range** | **Data sources** |
| --- | --- | --- |
| *Calibration* | | |
| AIDS diagnoses (overall and by sex) | 1979-2002 | INSPQ^35, 36^ |
| New HIV diagnoses (overall and by sex and age) | 2003-2020^*^ | INSPQ |
| New HIV diagnoses by CD4 cell count at diagnosis (overall and by sex and age)^†^ | 2013-2020^*^ | INSPQ |
| Proportion of new diagnoses recently tested (P12M; overall and by sex and age)^‡^ | 2003-2020^*^ | INSPQ |
| *Cross-validation* | | |
| HIV prevalence | 2005-2007 (Argus I)  2008-2010 (Argus II)  2017-2018 (Engage) | Argus (Montréal)  Engage (Montréal)^26^ |
| Percentage of PLHIV undiagnosed | 2017 – 2018 (Engage) | Engage (Montréal)^26, 38^ |
| Percentage of diagnosed PLHIV that initiated ART | 2005 – 2007 (Argus I)  2008 – 2010 (Argus II)  2017 – 2018 (Engage) | Argus (Montréal)  Engage (Montréal) |

Abbreviations: Institut national de santé publique du Québec (INSPQ); past 12 months (P12M); people living with HIV (PLHIV); antiretroviral treatment (ART).

^*^Due to the COVID-19 pandemic, the data from 2020 were excluded from model calibration in the main analyses and included in sensitivity analyses.

^†^The CD4 cell count contained missing values. These were assumed to be missing completely at random.

^‡^The testing data is categorized as *never previously tested*, *unsure when last tested*, *tested <12 months ago*, and *tested ≥12 months ago*. In calculating the proportion of new diagnoses recently tested, it is assumed that those reporting as *unsure when last tested* (<4% of observations) did not test in the past 12 months.

#### Calibration method: optimization

The calibration of our model was carried out using optimization under a Bayesian framework, allowing uncertainty in the estimated parameters to be reflected in the model’s predictions and ensuring identifiability of the unknown parameters given the number of degrees of freedom. Maximum a posteriori estimation (MAP), a Bayesian analog of maximum likelihood estimation that maximizes the joint posterior probability, can obtain estimators of unknown parameters in such optimization problems^39^. We specifically obtained the MAP parameter point estimates by minimizing the negative posterior log-likelihood of the model. The prior distributions placed over the unknown parameters are as formulated in Table S9. The model posterior likelihood is described below. As optimization searches over the entire set of real numbers for all parameters, we logit transformed and scaled the optimized values to ensure all parameter estimates were on the appropriate scale and within relevant bounds, as needed.

A two-step optimization method was performed. First, we used the Nelder-Mead algorithm with starting values randomly selected from the parameter prior distributions and a maximum of 10,000 iterations. We then used the BFGS algorithm, starting from the Nelder-Mead optimized parameter values and continuing to convergence. Secondly, we used sampling importance resampling (SIR) to approximate the posterior distribution, taking the final BFGS optimized parameter values as the posterior mode. The initial samples (n=50,000) from the posterior distribution were drawn from a multivariate *t*-distribution, using the estimated hessian matrix as the scale matrix. Using standardized importance weights as sampling probabilities, 1000 resamples were then taken without replacement, as suggested by Gelman et al.^40^ to be appropriate when few of the resulting importance weights are large. Posterior distributions are summarized by the median and the 2.5^th^ and 97.5^th^ percentiles estimated 95% credible intervals.

#### Model posterior likelihood

Let $\theta$ be the vector of model parameters. For each time *t* (specific to each outcome), age group *i*, and sex *j*, let $D_{t,j,i}$ correspond to outcomes observed in the data and $M_{t,j,i}$ correspond to outcomes predicted by the model.

## Number of reported AIDS cases

The observed number of reported AIDS cases, $D_{t,j}^{AIDS}$, a cumulative count over 1975-1998, followed a negative binomial distribution with mean and variance:

$E\left[ D_{t,j}^{AIDS} \right]=M_{t,j}^{AIDS}$ and$Var\left[ D_{t,j}^{AIDS} \right]=M_{t,j}^{AIDS}+\frac{{M_{t,j}^{AIDS}}^{2}}{r}$,

where $M_{t,j}^{AIDS}$ is the model-predicted number of AIDS diagnoses and the dispersion parameter, $r$, was fixed at 100. Reported AIDS cases $\left( D_{t,j}^{AIDS} \right)$, met the clinical case definition in use at the time of diagnosis^35, 36^. The definition was initially based on the diagnosis of opportunistic infections, changing over time as the list of these infections expanded, and a positive HIV test result when it was added to the case definition in 1985. In the model, we proxy this case definition by defining AIDS cases as individuals with a CD4<200 cells/µL. The annual model-predicted number of AIDS diagnoses $\left( M_{t,j}^{AIDS} \right)$ is taken as the sum of 1) the number of PLHIV that transition from the undiagnosed to diagnosed CD4<200 cells/µL compartment over a calendar year and 2) the number of PLHIV that are diagnosed and transition from the CD4 200-349 cells/µL compartment to the CD4<200 cells/µL compartment.

The observed likelihood function is as follows:

$$\begin{aligned} \mathcal{L}_{AIDS} \left( \theta| D_{t,j}^{AIDS} \right)= \prod_{t} \left( \frac{\Gamma\left( D_{t,j}^{AIDS}+r \right)}{\Gamma\left( D_{t,j}^{AIDS}+1 \right)\Gamma\left( r \right)} \right)\left( \frac{r}{M_{t,j}^{AIDS}+r} \right)^{r}\left( \frac{M_{t,j}^{AIDS}}{M_{t,j}^{AIDS}+r} \right)^{D_{t,j}^{AIDS}}\#(9.1) \end{aligned}$$

## Number of reported new HIV diagnoses

The observed number of reported new HIV diagnoses, $D_{t,i,j}^{HIV}$, for $t\in\left[ 2003, \ldots, 2020 \right]$, similarly followed a negative binomial distribution with mean and variance:

$E\left[ D_{t,i,j}^{HIV} \right]=M_{t,i,j}^{HIV}$ and $Var\left[ D_{t,i,j}^{HIV} \right]=M_{t,i,j}^{HIV}+\frac{{M_{t,i,j}^{HIV}}^{2}}{r}$,

where $M_{t,i,j}^{HIV}$ is the model-predicted number of HIV diagnoses and the dispersion parameter $r$ remained fixed at 100. The observed likelihood function is as follows:

$$\begin{aligned} \mathcal{L}_{HIV} \left( \theta| D_{t,i,j}^{HIV} \right)= \prod_{t} \prod_{i} \left( \frac{\Gamma\left( D_{t,i,j}^{HIV}+r \right)}{\Gamma\left( D_{t,i,j}^{HIV}+1 \right)\Gamma\left( r \right)} \right)\left( \frac{r}{M_{t,i,j}^{HIV}+r} \right)^{r}\left( \frac{M_{t,i,j}^{HIV}}{M_{t,i,j}^{HIV}+r} \right)^{D_{t,i,j}^{HIV}}\#(9.2) \end{aligned}$$

## Proportion of reported new HIV diagnoses that recently tested negative

The observed proportion of reported new HIV diagnoses recently tested negative, $p_{D_{t,i,j}^{test}}$, for $t\in\left[ 2003, \ldots, 2020 \right]$, followed a binomial distribution. The observed likelihood is:

$$\begin{aligned} \mathcal{L}_{test} \left( \theta| p_{D_{t,i,j}^{test}} \right)=\prod_{t} \prod_{i} \binom{D_{t,i,j}^{HIV}}{D_{t,i,j}^{test}}{p_{M_{t,i,j}^{test}}}^{D_{t,i,j}^{test}}\left( 1-p_{M_{t,i,j}^{test}} \right)^{D_{t,i,j}^{HIV}-D_{t,i,j}^{test}}\#\left( 9.3 \right) \end{aligned}$$

where $D_{t,i,j}^{test}$ indicates the observed number of reported new HIV diagnoses that received a negative HIV test in the past 12 months and $p_{M_{t,i,j}^{test}}= M_{t,i,j}^{test}/M_{t,i,j}^{HIV}$ is the model-predicted proportion of reported new HIV diagnoses that tested negative in the past 18 months.$M_{t,i,j}^{test}$, the model-predicted number of new HIV diagnoses that tested negative in the past 18 months, is determined using compartments that specifically track HIV testing histories (Figure S3).

|  |
| --- |
| **Figure S3. Diagram of the main inter-compartmental flows for HIV testing histories.** The index *i* indicates age group (15-19, 20-24, 25-29, 30-34, 35-39, 40-44, 45-49, 50-54, 55-59, 60-64, 65+), *j* indicates sex (male, female), and *k* indicates disease stage (1=CD4≥500 cells/µL; 2=CD4 350-499 cells/µL; 3=CD4 200-349 cells/µL; and 4=CD4<200 cells/µL). Susceptible individuals that do not acquire HIV can transition into the recently tested negative (<18 months ago) and susceptible compartment at the rate $\tau_{1}\left( t \right)$. Those individuals can progress back into the not recently tested negative compartments (≥18 months ago) via the susceptible, primary infection, and undiagnosed compartments at a rate of 1/1.5 years^-1^. |

## Number of reported new HIV diagnoses by CD4 cell count

The observed number of new HIV diagnoses per CD4 cell count category $D_{t,i,j, k}^{CD4}$, for $t\in\left[ 2013, \ldots, 2020 \right]$ and $k\in\left[ 1, 2, 3, 4 \right]$, follows a multinomial distribution. The observed likelihood is as follows:

$$\begin{aligned} \mathcal{L}_{CD4} \left( \theta|D_{t,i,j, k=1}^{CD4}, \ldots, D_{t,i,j, k=4}^{CD4} \right)=\prod_{t} \prod_{i} \frac{N_{t,i,j}^{CD4}}{D_{t,i,j, k=1}^{CD4}! \cdots D_{t,i,j, k=4}^{CD4}!}\prod_{k} p_{t,i,j,k}^{M_{t,i,j, k}^{CD4}}\#\left( 9.4 \right) \end{aligned} ,$$

where $N_{t,i,j}^{CD4}=\sum_{k} D_{t,i,j, k}^{CD4}$ and $p_{t,i,j,k}^{M_{t,i,j, k}^{CD4}}$ is the model-predicted proportion of reported new HIV diagnoses per CD4 cell count category.

The overall model posterior likelihood is then taken as the prior likelihood multiplied by the likelihood of all data.

# Final models

#### Attributes of the modeled incidence M-spline

**Table S13:** Number and placement of knots included in incidence M-spline of final models.

| **Location** | **Sex** | **N knots** | **Knot location** |
| --- | --- | --- | --- |
| *Men who have sex with men* | | | |
| Montréal^*^ | Male | 4 | 1984, 1993, 2002, 2011 (equally spaced)^†^ |
| Province of Québec^*^ | Male | 4 | 1985, 1990, 1996, 2000 (focused where increased flexibility needed) |
| *People who inject drugs (includes active and past injectors)* | | | |
| Montréal | Overall | 3 | 1997, 2003, 2007 (focused where more available data) |
|  | Male | 3 | 1985, 1996, 2000 (focused where increased flexibility needed) |
|  | Female | 4 | 1985, 1990, 1996, 2000 (focused where increased flexibility needed) |
| Province of Québec | Overall | 4 | 1984, 1993, 2002, 2011 (equally spaced) |
|  | Male | 3 | 1986.3, 1997.5, 2008.8 (equally spaced) |
|  | Female | 3 | 1997, 2003, 2007 (focused where more available data) |

^*^The same number and placement of knots was used when estimating incidence by age.

^†^The final models were selected considering both the WAIC and LOOIC.

#### Sensitivity analyses

We assessed the sensitivity of the model to some of the assumed parameter inputs. First, we varied the assumed ART initiation rates among PWID, as empirical information to inform parameters was not available. We further allowed the ART initiation rates to vary between the treatment-eligible CD4 cell count categories over 1996-2013, setting the 1996-2012 ART initiation rates among those with CD4 350-200 cells/µL equal to 0.75 and 0.5 times that of those with CD4 <200 cells/µL. From 2013 onward, we maintained the assumption of equal rates across categories since treatment initiation is rapid from then on. Lastly, we assessed alternate disease progression parameters available in the literature to ensure the chosen inputs did not impact the results. In all, we found that our model was not very sensitive to these assumptions, as none of the results were meaningfully impacted by these changes.

# Additional figures of model fits (calibration outcomes)

## Men who have sex with men

| 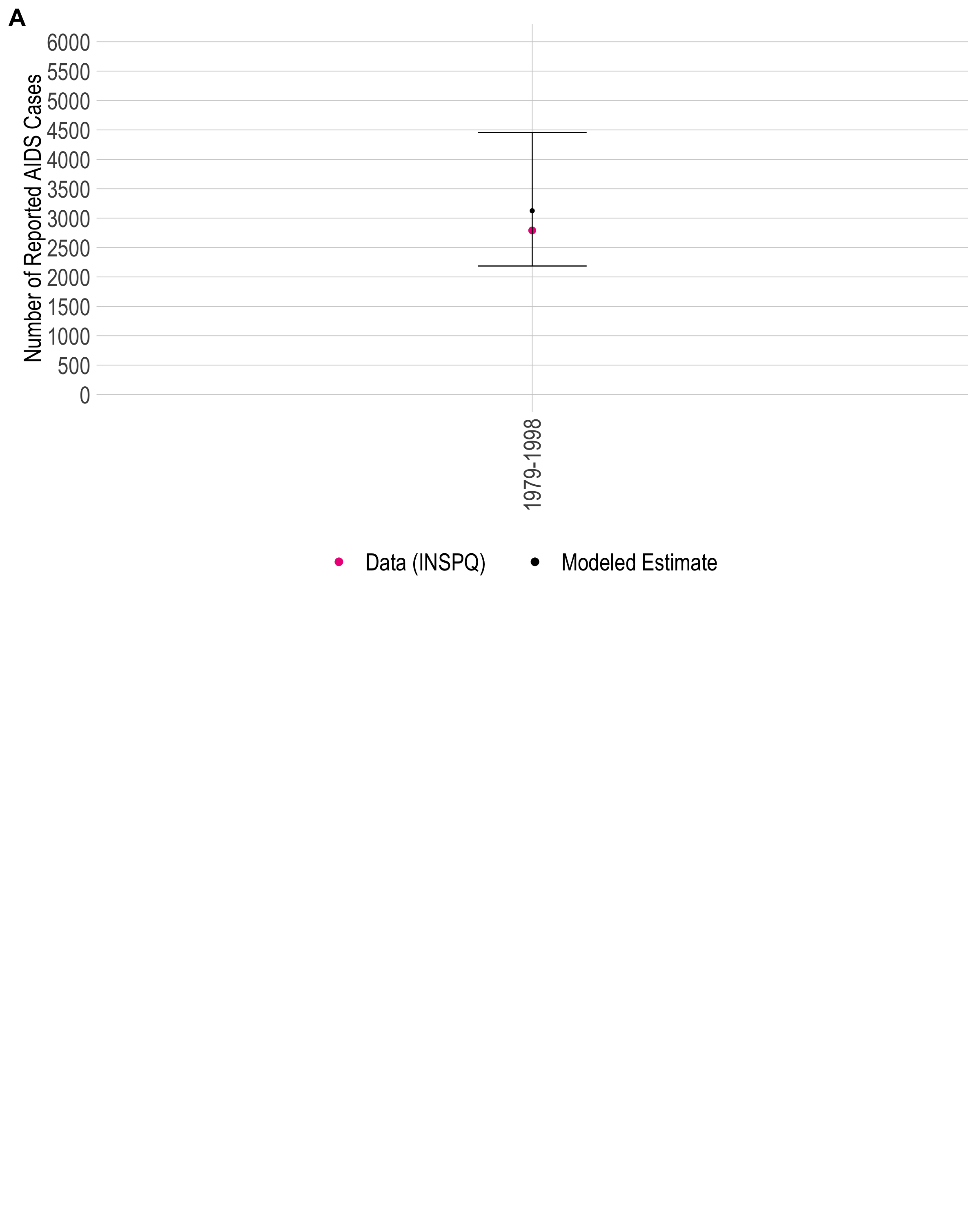 |
| --- |
| 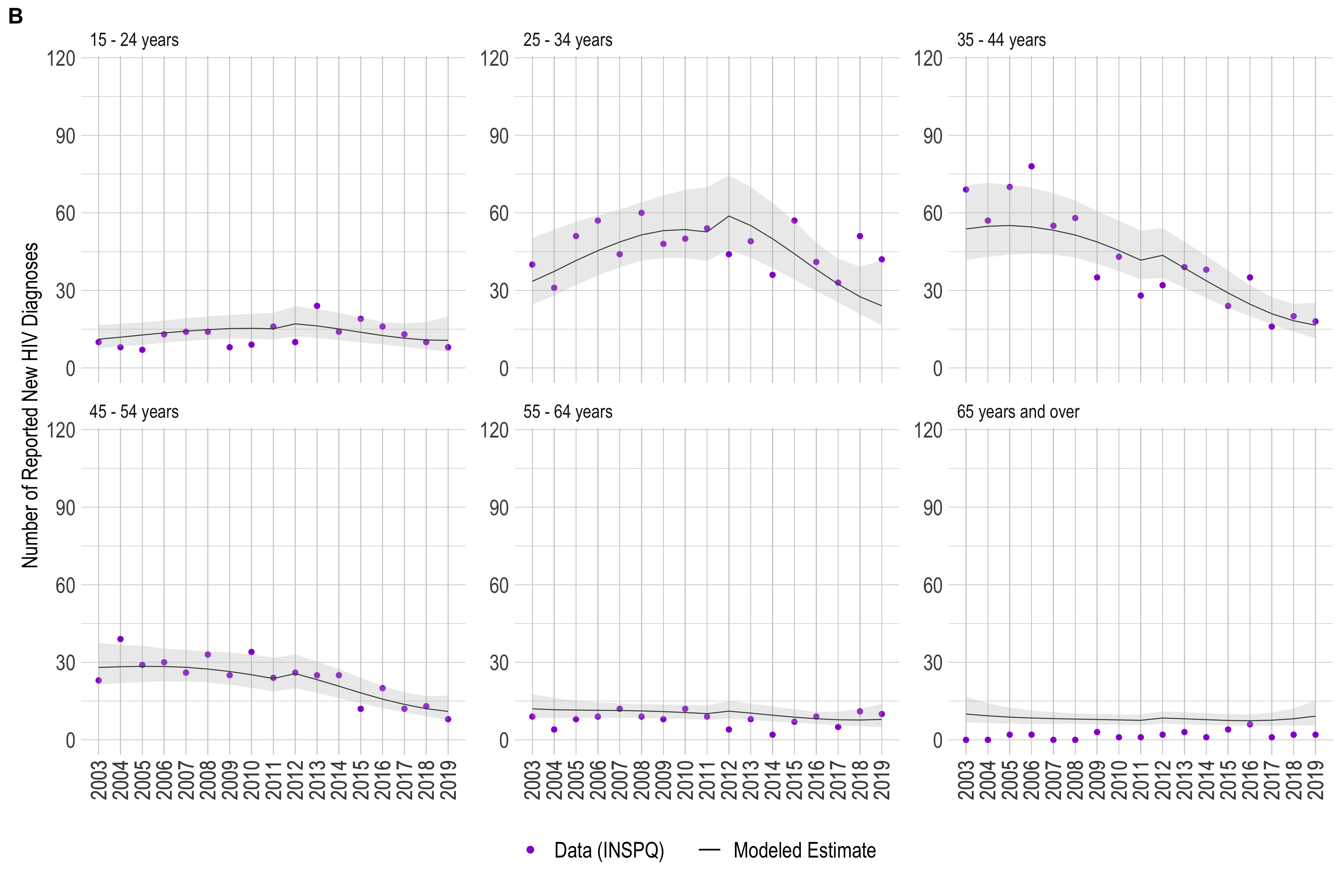 |
| 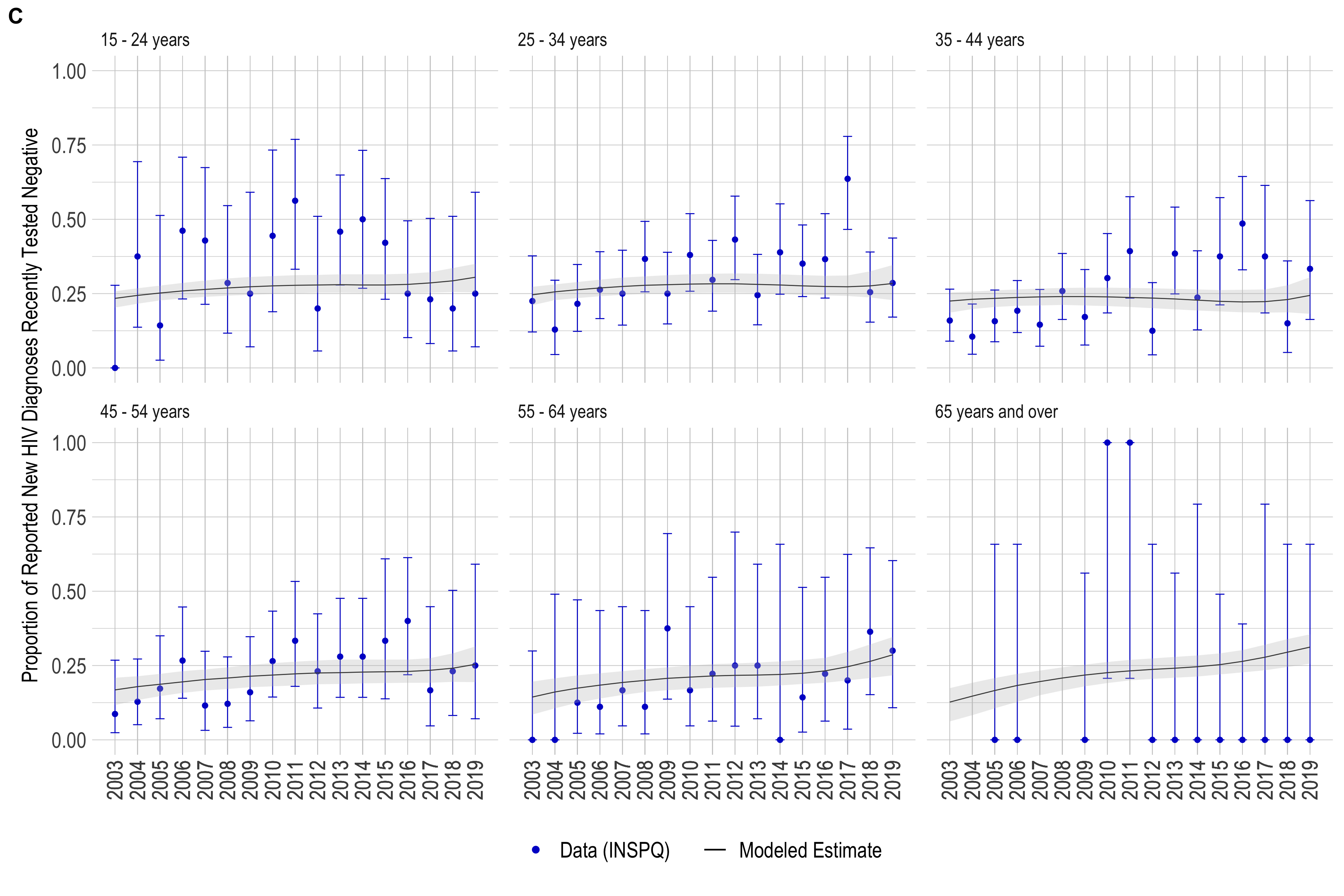 |
| 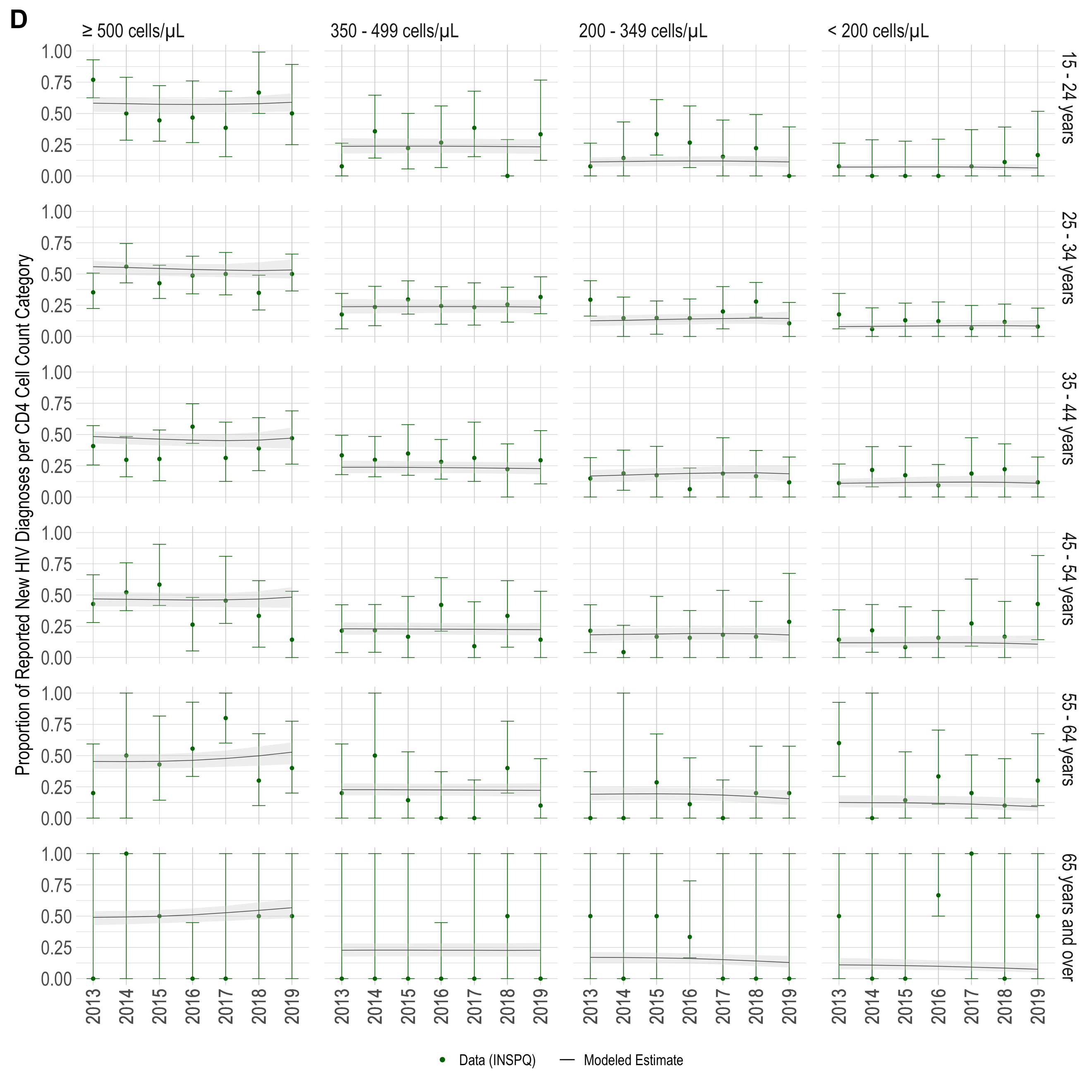 |
| **Figure S4.** Model fits to the age-stratified calibration outcomes among men who have sex with men in Montréal: A) number of reported AIDS cases; B) number of reported new HIV diagnoses by age group; C) proportion of reported new HIV diagnoses that recently tested negative by age group; and D) proportion of reported new HIV diagnoses per CD4 cell count category and age group. The black points and lines display the model-predicted outcomes, with the black bars and grey bands showing their corresponding 95% credible intervals. The coloured points and bars display the outcomes from the *Institut national de santé publique du Québec* (INSPQ) data and their corresponding 95% confidence intervals, where applicable. |

| 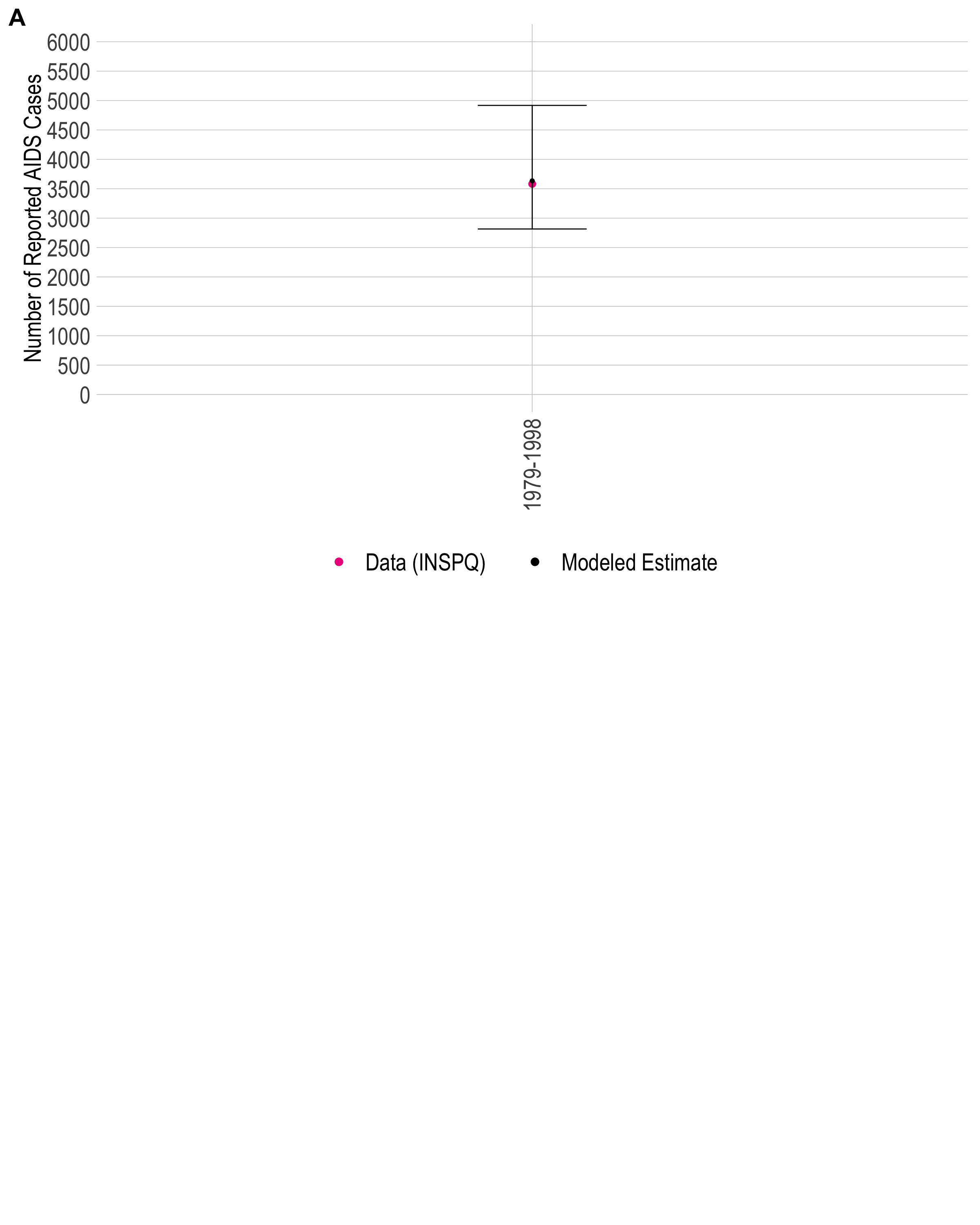 |
| --- |
| 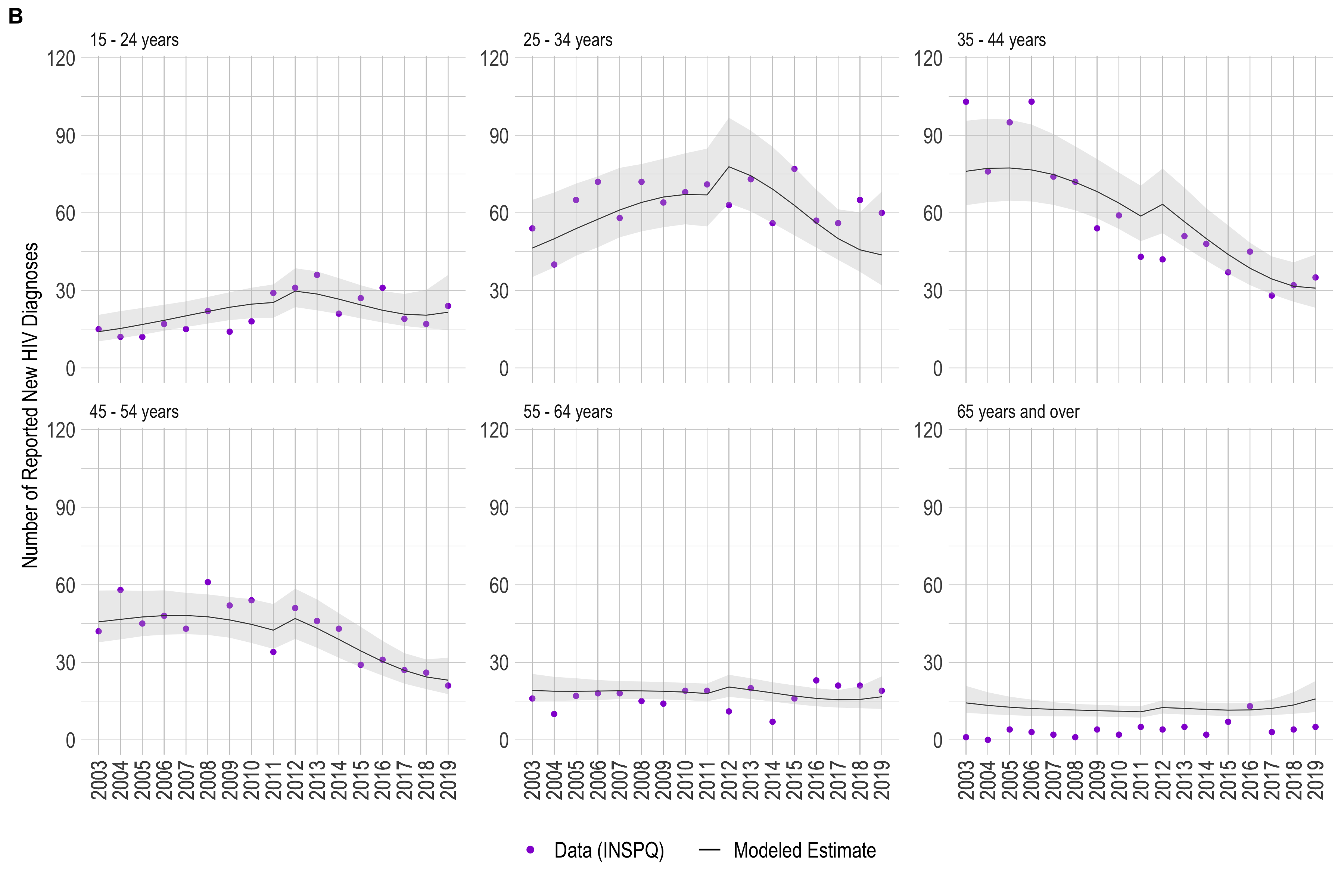 |
| 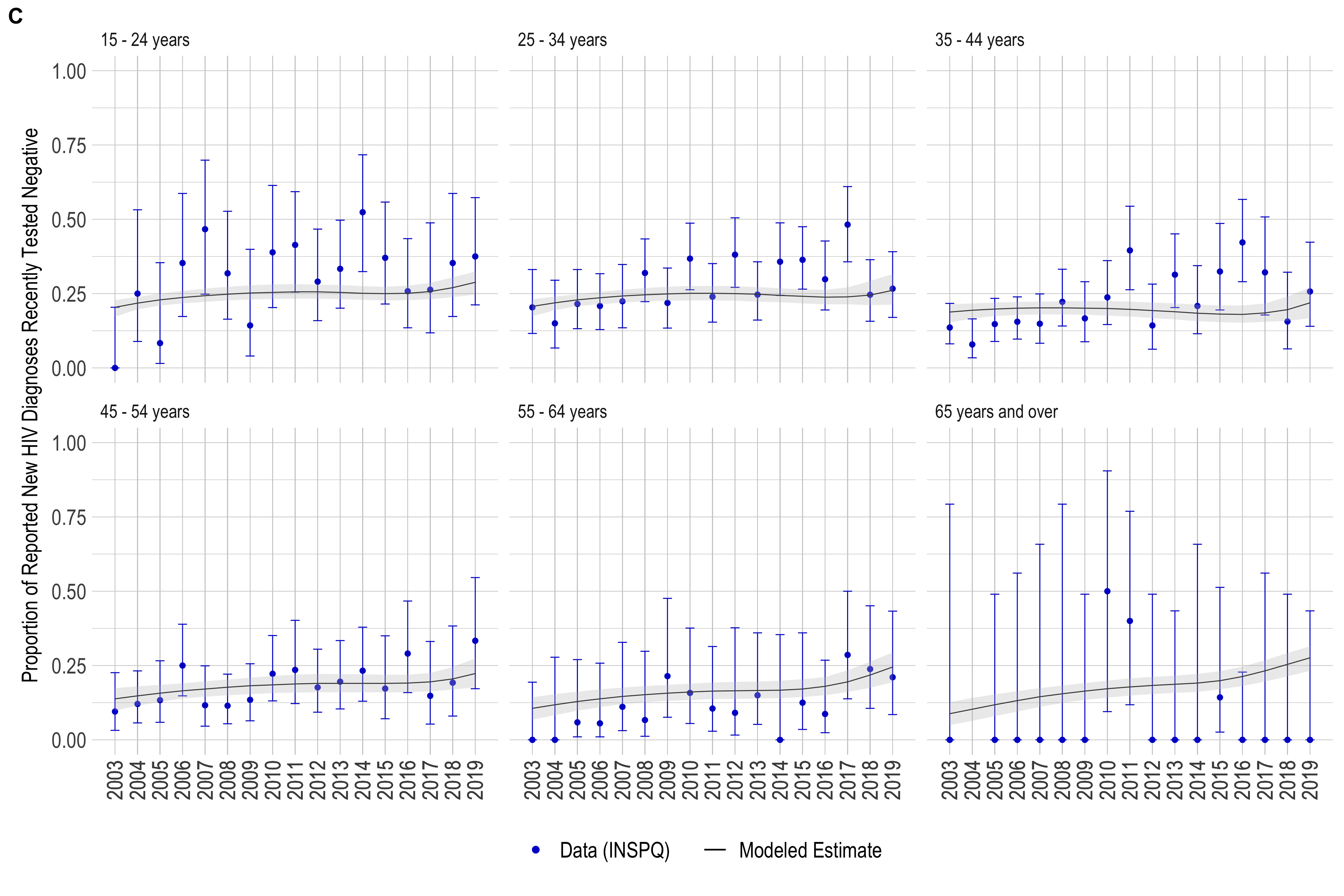 |
| 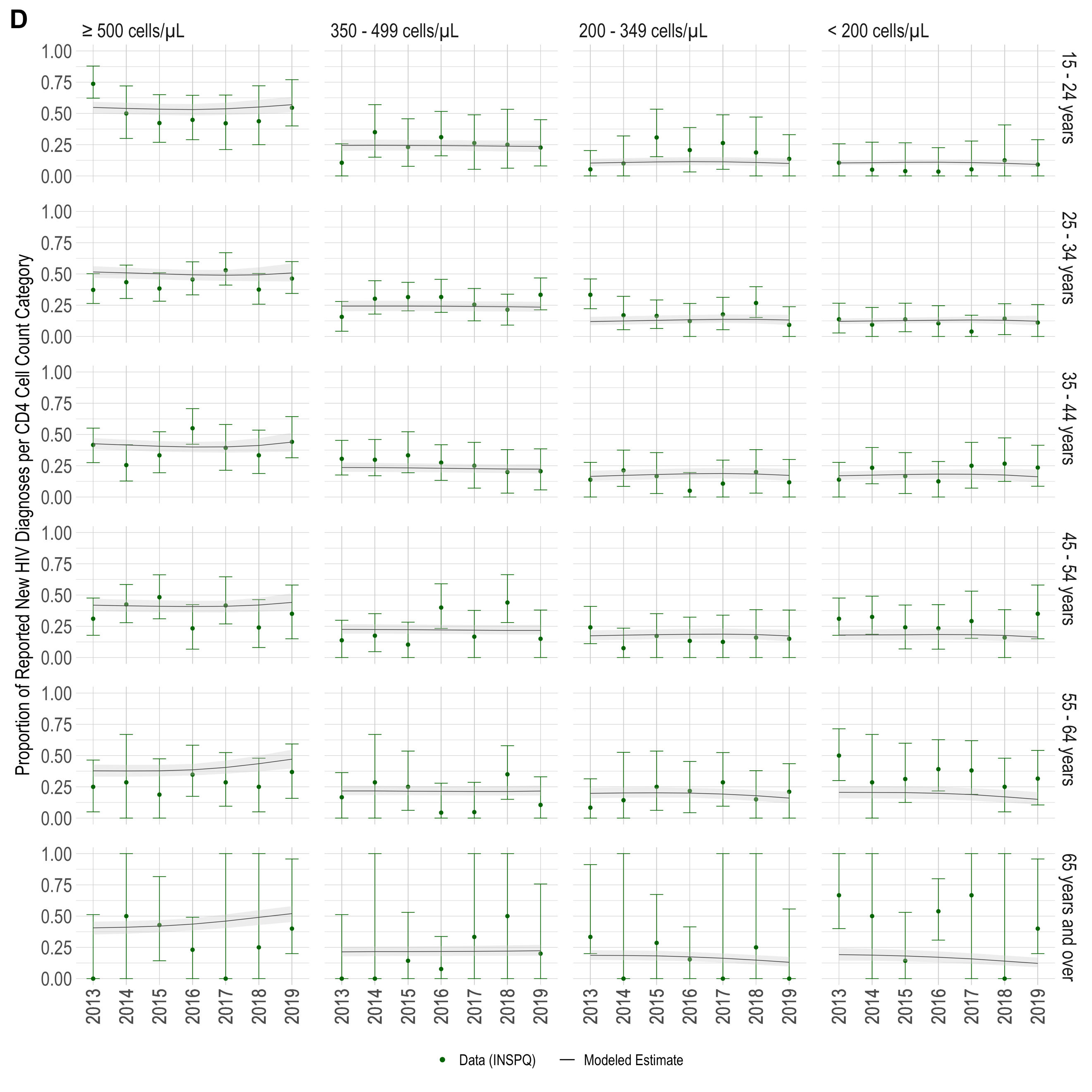 |
| **Figure S5.** Model fits to the age-stratified calibration outcomes among men who have sex with men in the province of Québec: A) number of reported AIDS cases; B) number of reported new HIV diagnoses by age group; C) proportion of reported new HIV diagnoses that recently tested negative by age group; and D) proportion of reported new HIV diagnoses per CD4 cell count category and age group. The black points and lines display the model-predicted outcomes, with the black bars and grey bands showing their corresponding 95% credible intervals. The coloured points and bars display the outcomes from the *Institut national de santé publique du Québec* (INSPQ) data and their corresponding 95% confidence intervals, where applicable. |

## People who inject drugs

| 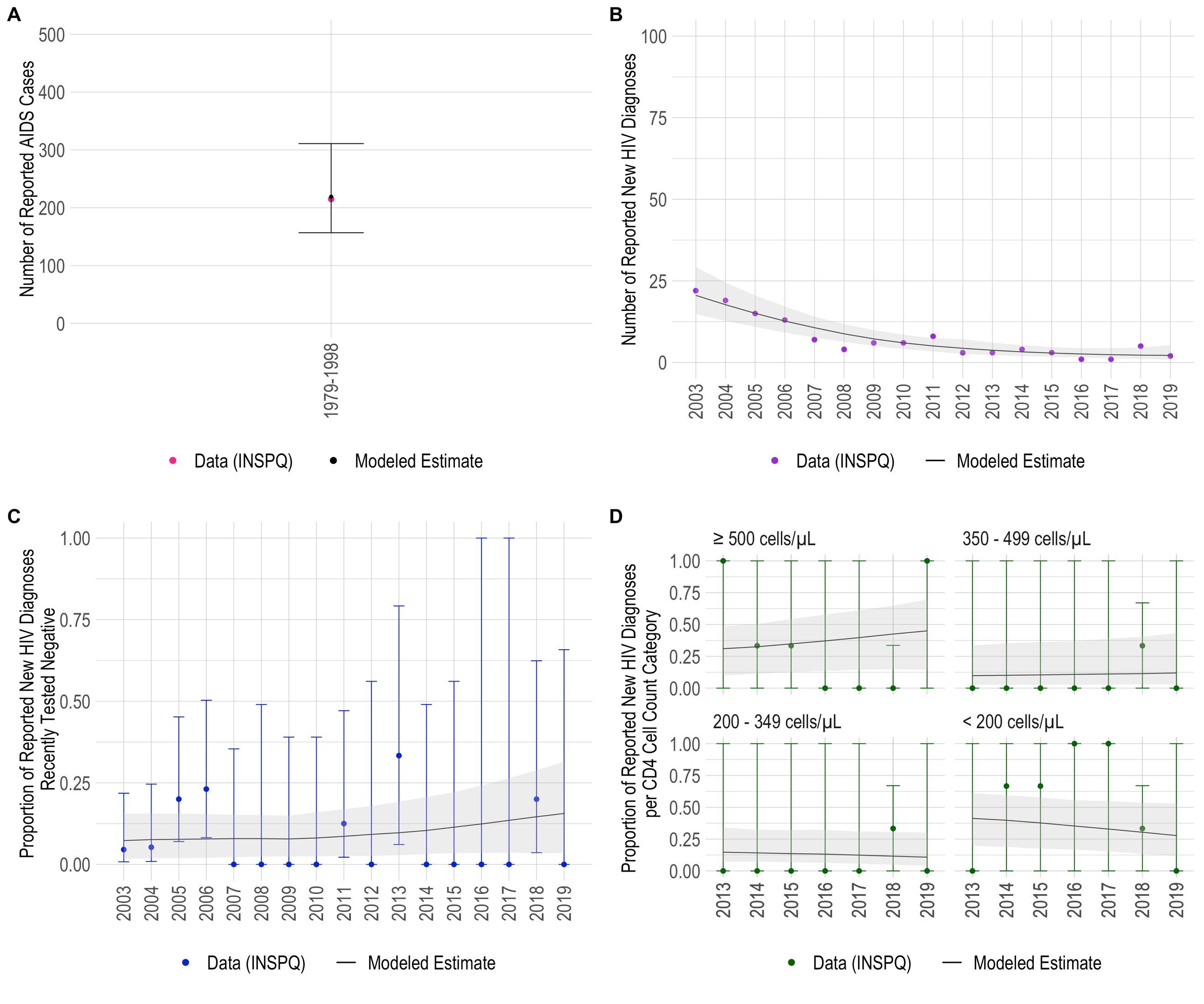 |
| --- |
| **Figure S6.** Model fits to the calibration outcomes among males who injected drugs in Montréal: A) number of reported AIDS cases; B) number of reported new HIV diagnoses; C) proportion of reported new HIV diagnoses that recently tested negative; and D) proportion of reported new HIV diagnoses per CD4 cell count category. The black points and lines display the model-predicted outcomes, with the black bars and grey bands showing their corresponding 95% credible intervals. The coloured points and bars display the outcomes from the *Institut national de santé publique du Québec* (INSPQ) data and their corresponding 95% confidence intervals, where applicable. |

| 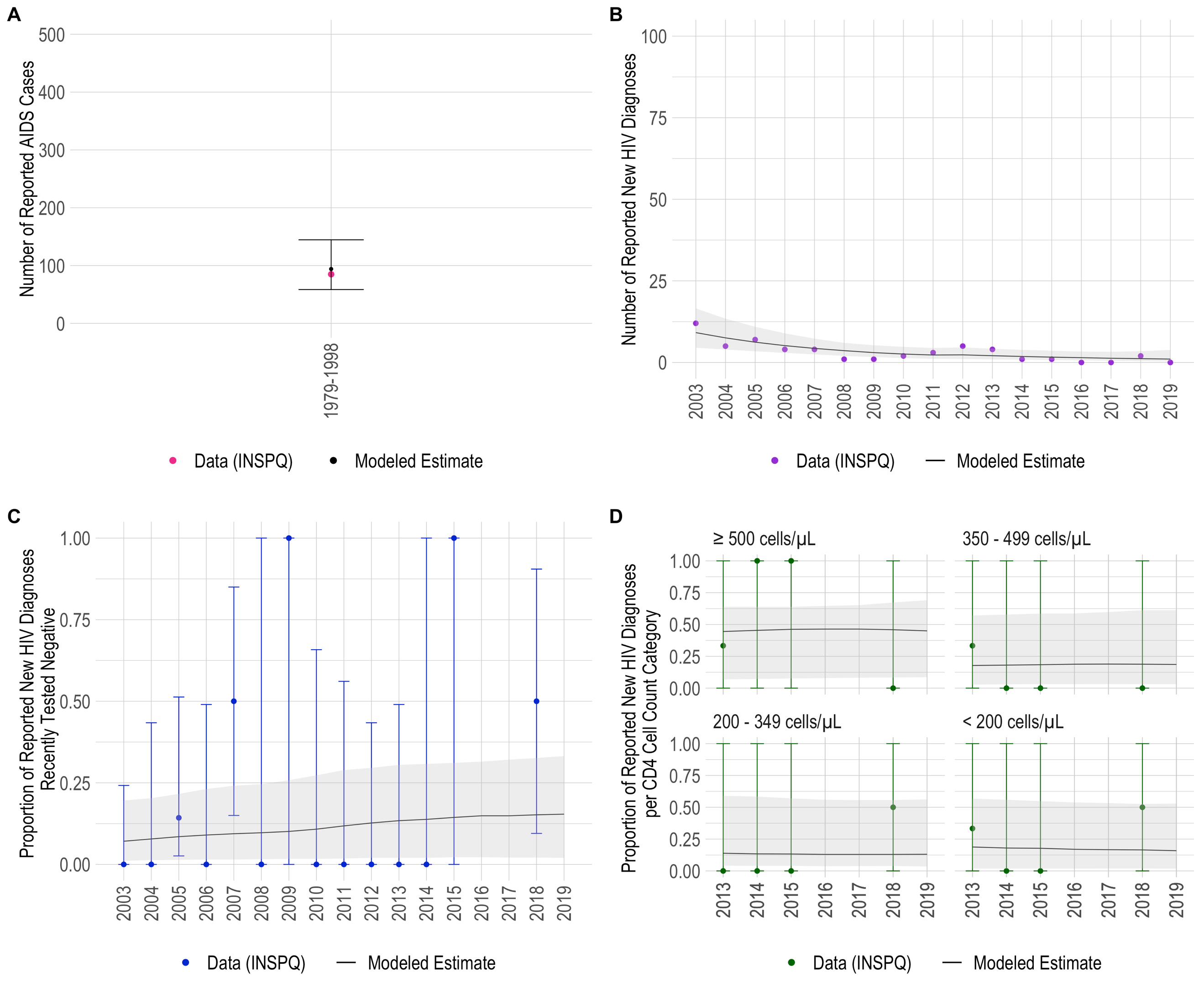 |
| --- |
| **Figure S7.** Model fits to the calibration outcomes among females who injected drugs in Montréal: A) number of reported AIDS cases; B) number of reported new HIV diagnoses; C) proportion of reported new HIV diagnoses that recently tested negative; and D) proportion of reported new HIV diagnoses per CD4 cell count category. The black points and lines display the model-predicted outcomes, with the black bars and grey bands showing their corresponding 95% credible intervals. The coloured points and bars display the outcomes from the *Institut national de santé publique du Québec* (INSPQ) data and their corresponding 95% confidence intervals, where applicable. |

| 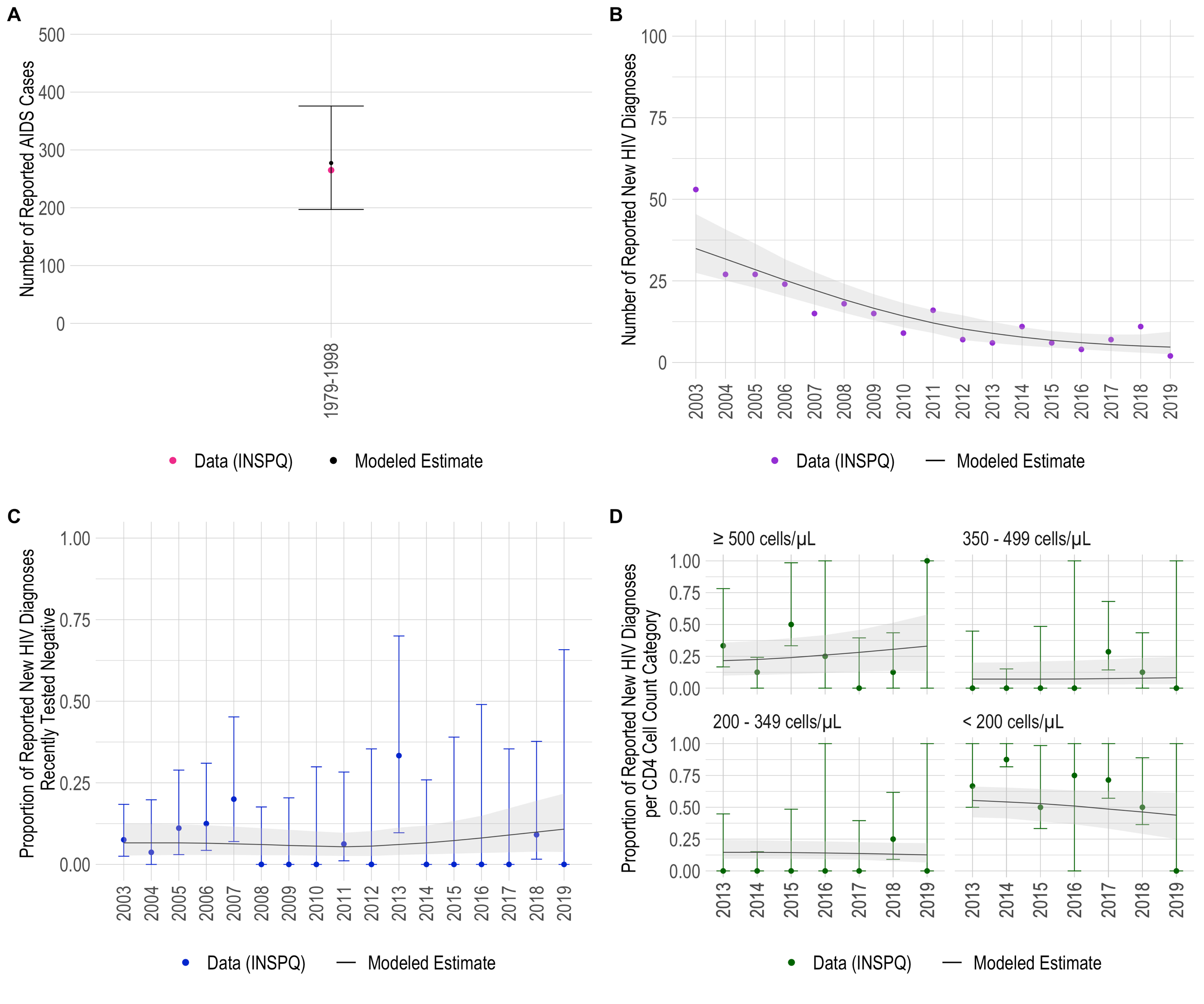 |
| --- |
| **Figure S8.** Model fits to the calibration outcomes among males who injected drugs in the province of Québec: A) number of reported AIDS cases; B) number of reported new HIV diagnoses; C) proportion of reported new HIV diagnoses that recently tested negative; and D) proportion of reported new HIV diagnoses per CD4 cell count category. The black points and lines display the model-predicted outcomes, with the black bars and grey bands showing their corresponding 95% credible intervals. The coloured points and bars display the outcomes from the *Institut national de santé publique du Québec* (INSPQ) data and their corresponding 95% confidence intervals, where applicable. |

| 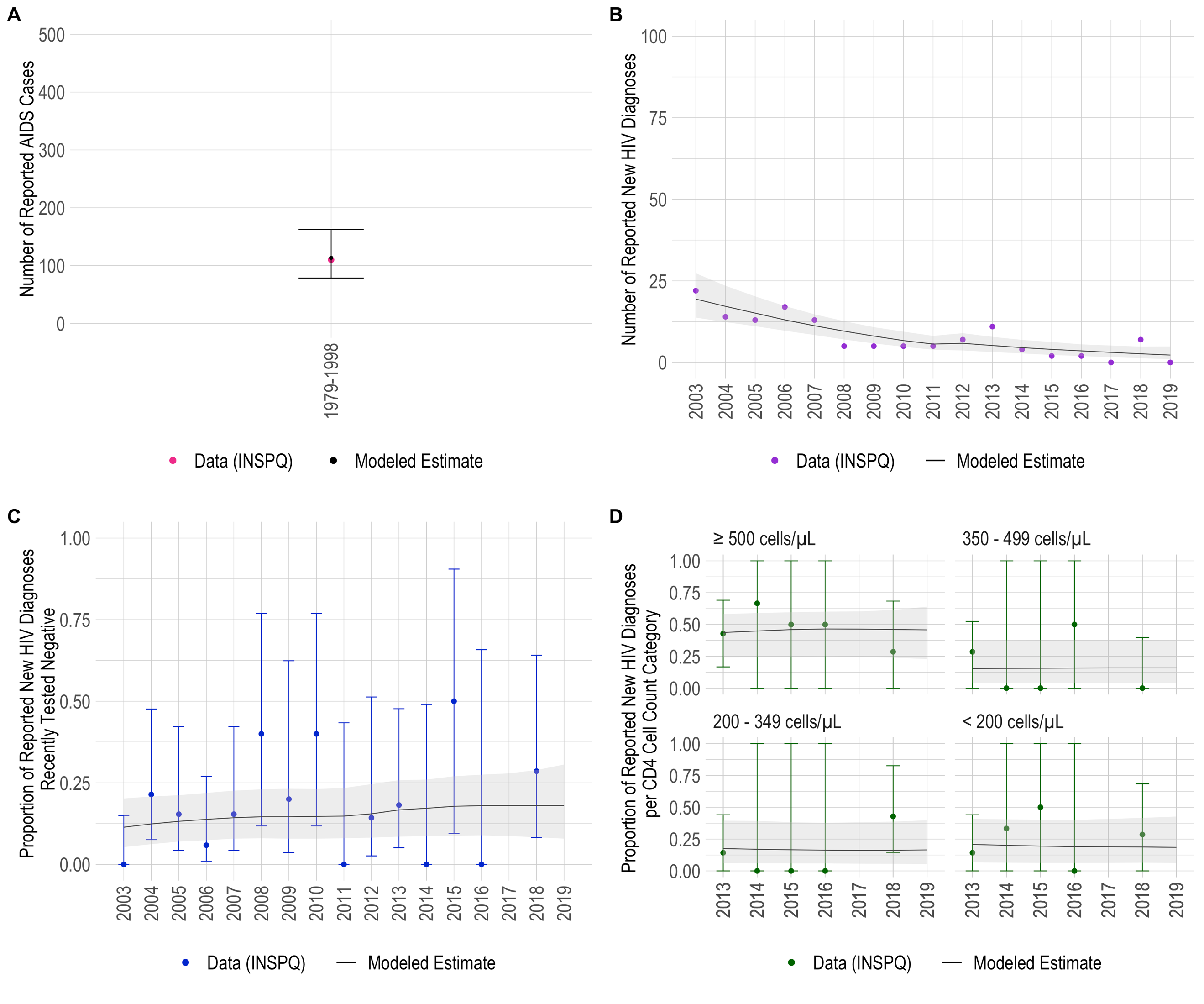 |
| --- |
| **Figure S9.** Model fits to the calibration outcomes among females who injected drugs in the province of Québec: A) number of reported AIDS cases; B) number of reported new HIV diagnoses; C) proportion of reported new HIV diagnoses that recently tested negative; and D) proportion of reported new HIV diagnoses per CD4 cell count category. The black points and lines display the model-predicted outcomes, with the black bars and grey bands showing their corresponding 95% credible intervals. The coloured points and bars display the outcomes from the *Institut national de santé publique du Québec* (INSPQ) data and their corresponding 95% confidence intervals, where applicable. |

# Additional results: main analyses

## Men who have sex with men

| **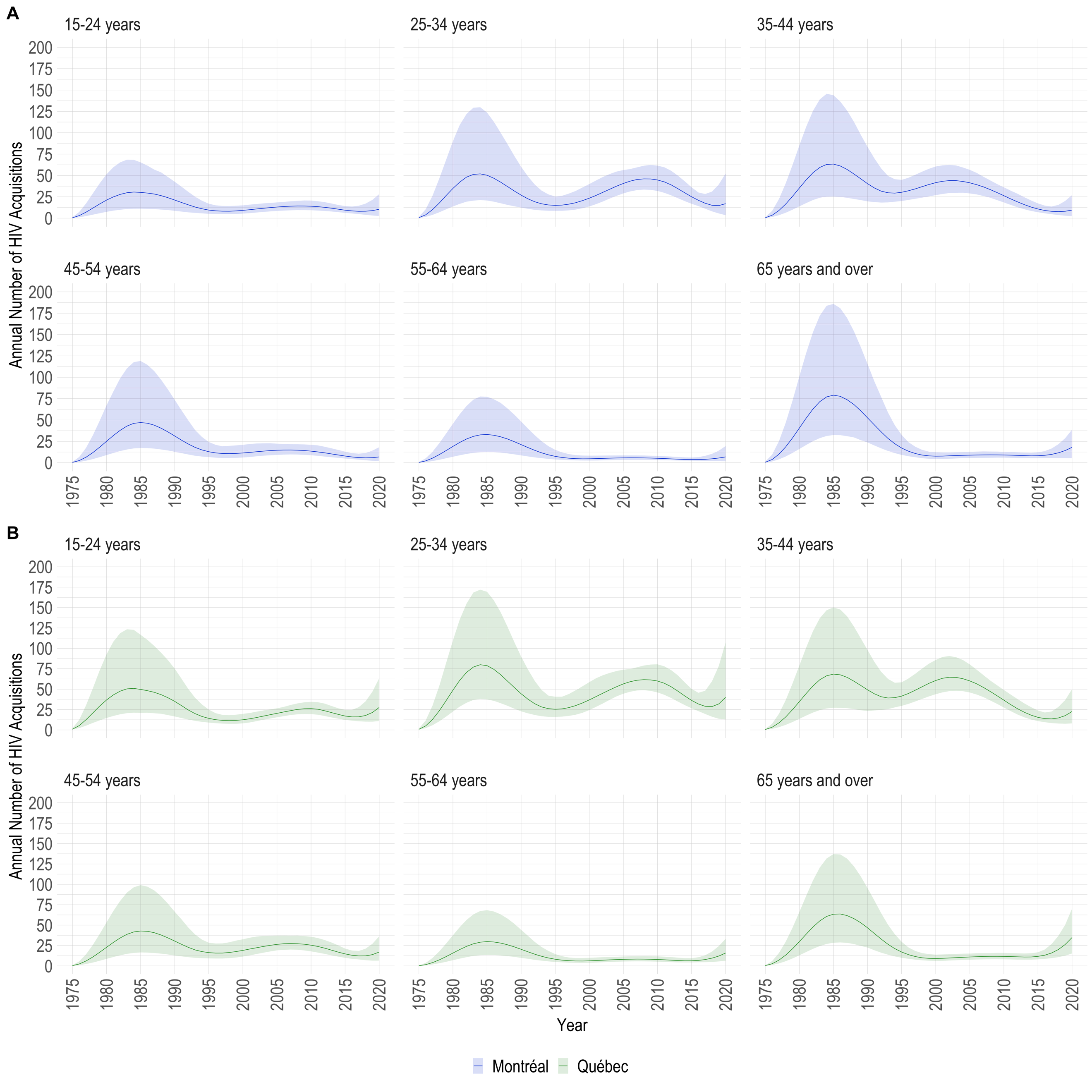** |
| --- |
| **Figure S10.** Estimated age-stratified annual number of HIV acquisitions over 1975-2020 among men who have sex with men in Montréal and the province of Québec, with incidence estimated per 10-year age group. The coloured lines and bands display the posterior median and 95% credible intervals, respectively, with blue representing estimates from the Montréal region (panel A) and green representing estimates for the whole province (panel B). |
| 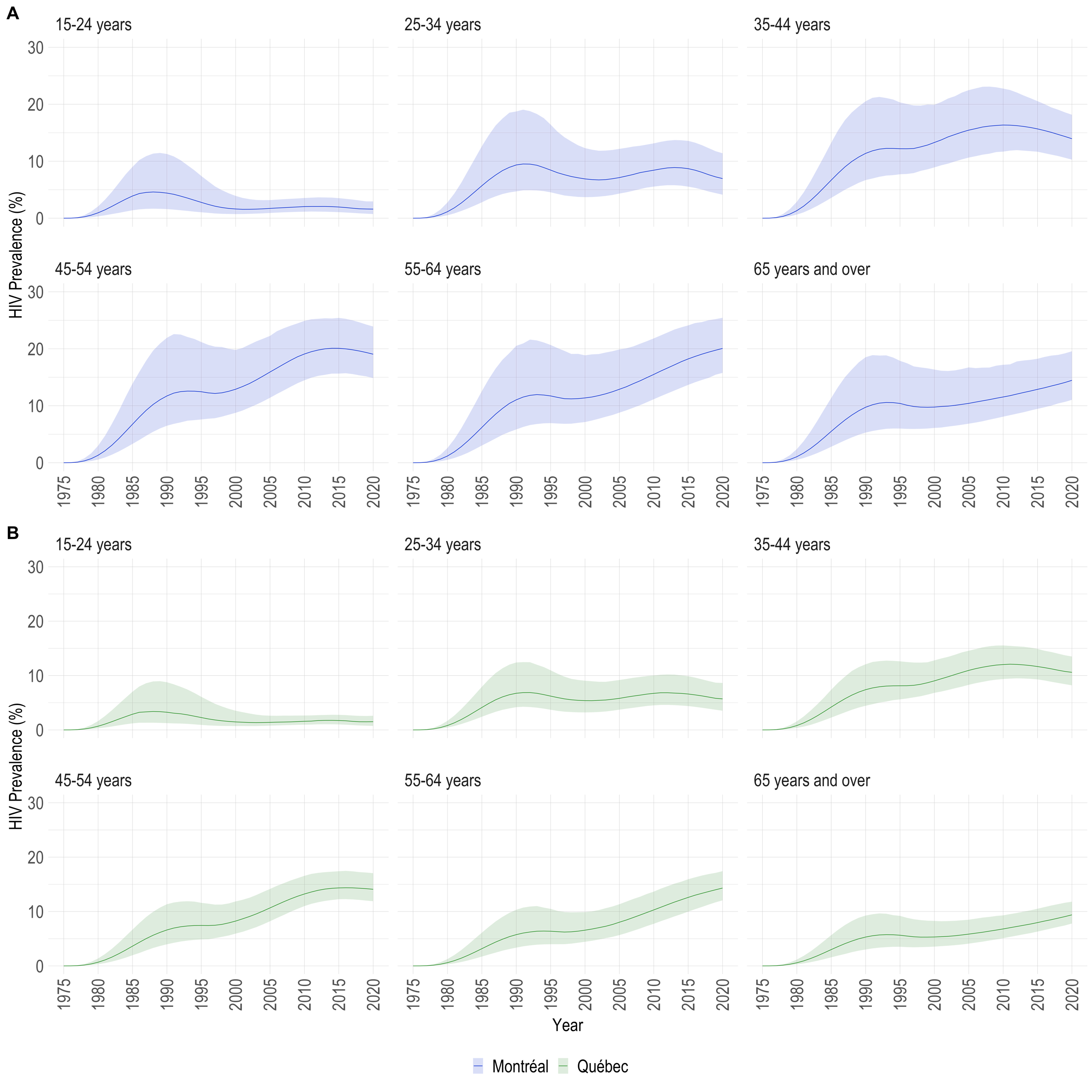 |
| **Figure S11.** Estimated age-stratified HIV prevalence over 1975-2020 among men who have sex with men (MSM) in Montréal and the province of Québec, by 10-year age groups. The coloured lines and bands display the posterior median and 95% credible intervals, respectively, with blue representing estimates from the Montréal region (panel A) and green representing estimates for all of Québec (panel B). |

| 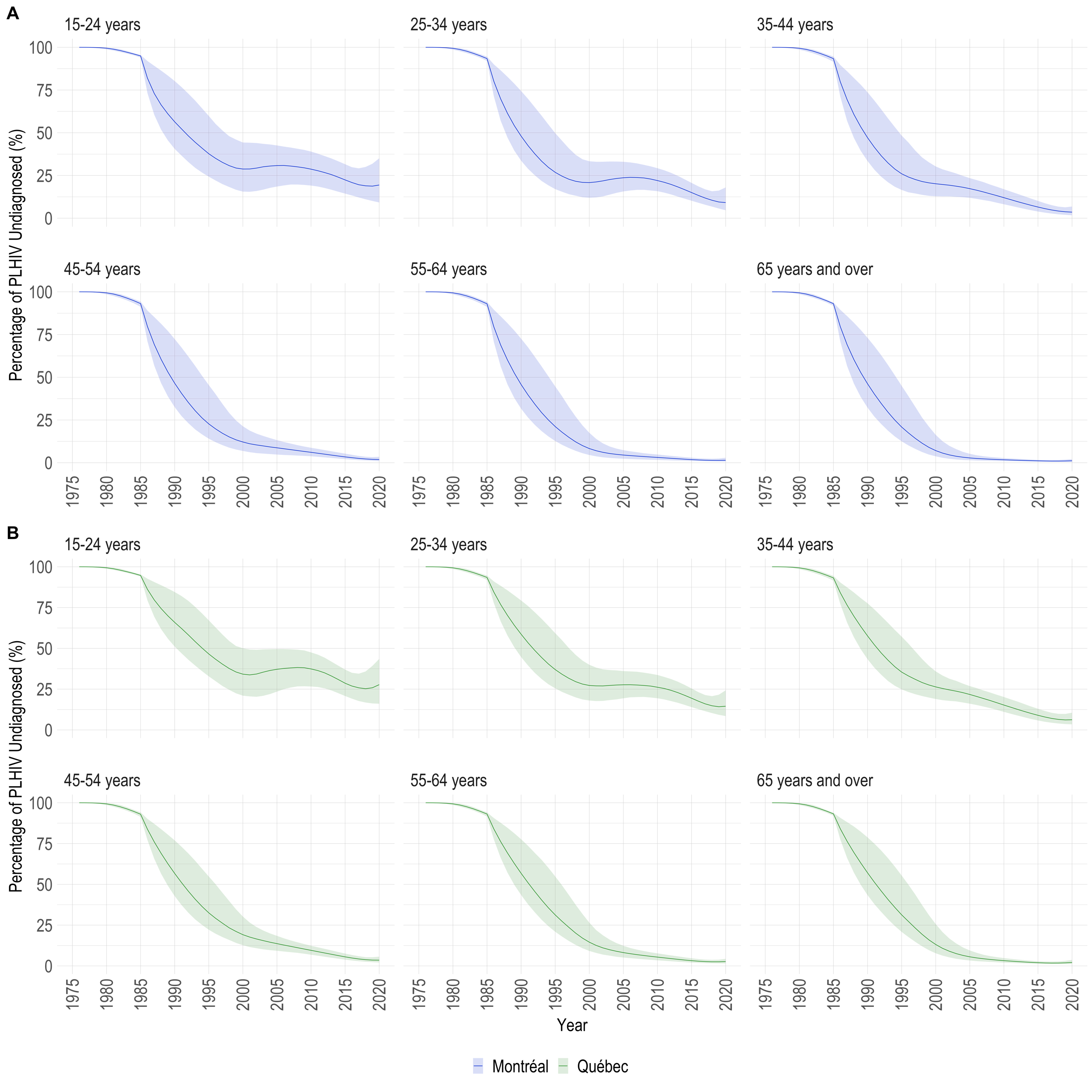 |
| --- |
| **Figure S12.** Estimated age-stratified percentage of people living with HIV (PLHIV) undiagnosed over 1975-2020 among men who have sex with men (MSM) in Montréal and the province of Québec, by 10-year age groups. The coloured lines and bands display the posterior median and 95% credible intervals, respectively, with blue representing estimates from the Montréal region (panel A) and green representing estimates for all of Québec (panel B). |
| 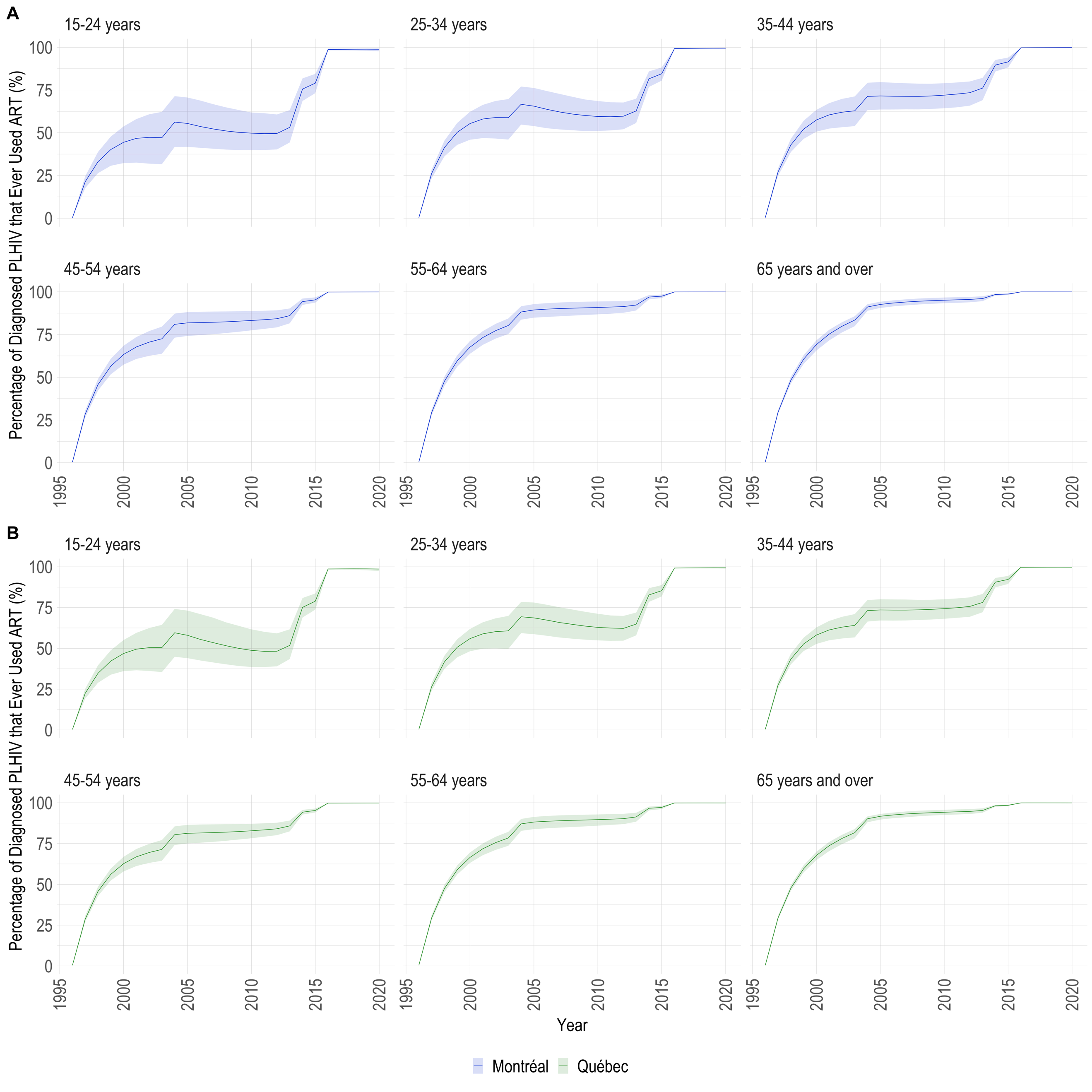 |
| **Figure S13.** Estimated age-stratified percentage of diagnosed people living with HIV (PLHIV) that ever used antiretroviral treatment (ART) over 1975-2020 among men who have sex with men (MSM) in Montréal and the province of Québec, by 10-year age groups. The coloured lines and bands display the posterior median and 95% credible intervals, respectively, with blue representing estimates from the Montréal region (panel A) and green representing estimates for all of Québec (panel B). |

## People who inject drugs

| 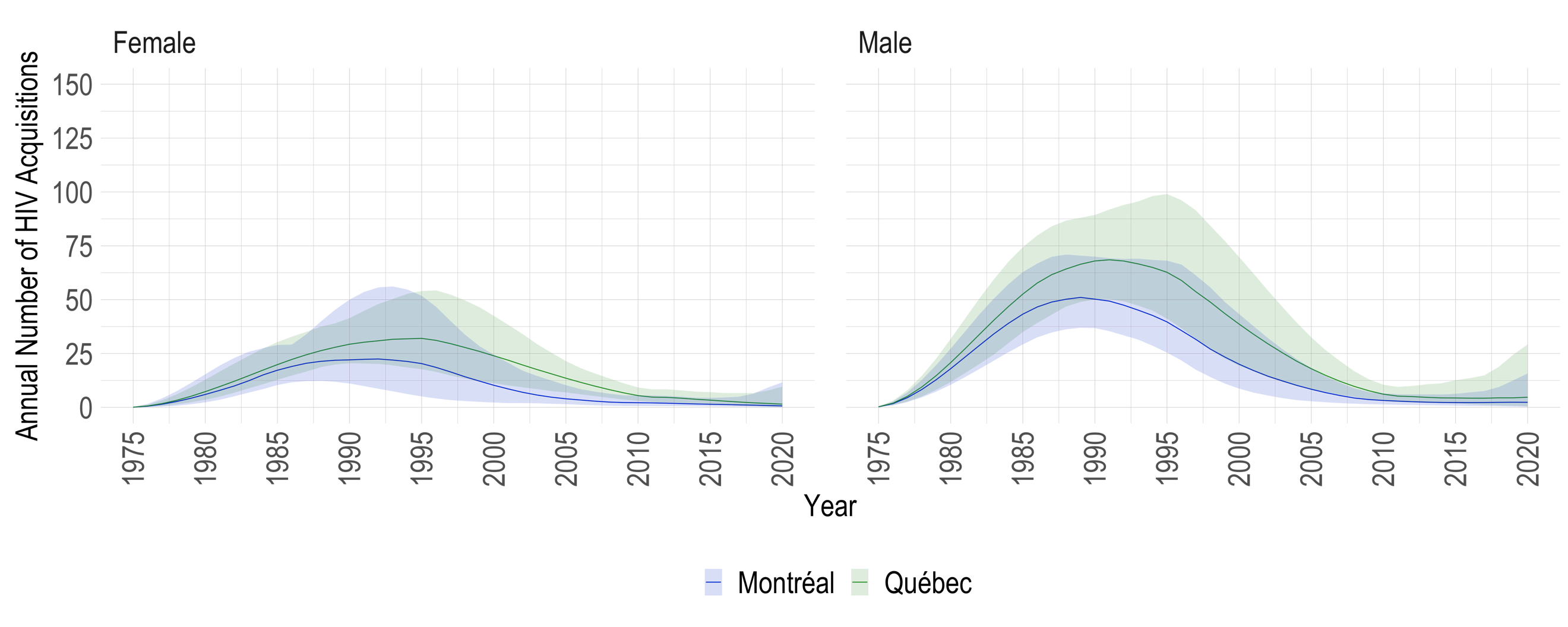 |
| --- |
| **Figure S14.** Estimated annual number of HIV acquisitions over 1975-2020 among active females (panel A) and males (panel B) who injected drugs (PWID) in Montréal and the province of Québec. The coloured lines and bands display the posterior median and 95% credible intervals, respectively, with blue representing estimates from the Montréal region and green representing estimates for all of Québec. |

| 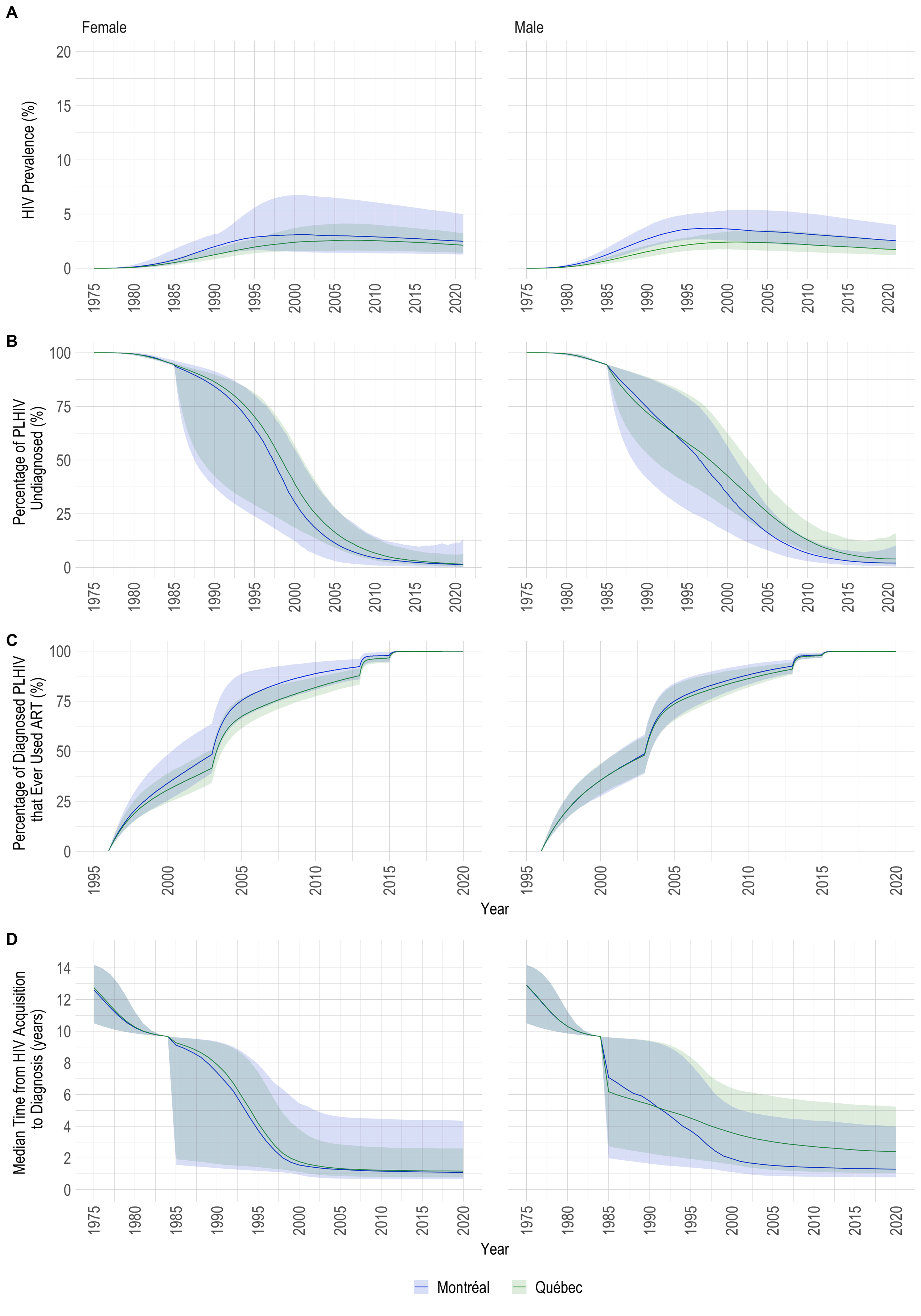 |
| --- |
| **Figure S15.** Estimated HIV prevalence (panel A), percentage of people living with HIV (PLHIV) undiagnosed (Panel B), percentage of diagnosed PLHIV that ever used antiretroviral treatment (ART; panel C), and average time from HIV acquisition to diagnosis (panel D) over 1975-2020 among females and males who ever injected drugs (PWID) overall in Montréal and the province of Québec. The coloured lines and bands display the posterior median and 95% credible intervals, respectively, with blue representing estimates from the Montréal region and green representing estimates for all of Québec. |

## Incidence figure among MSM including available comparison points

| 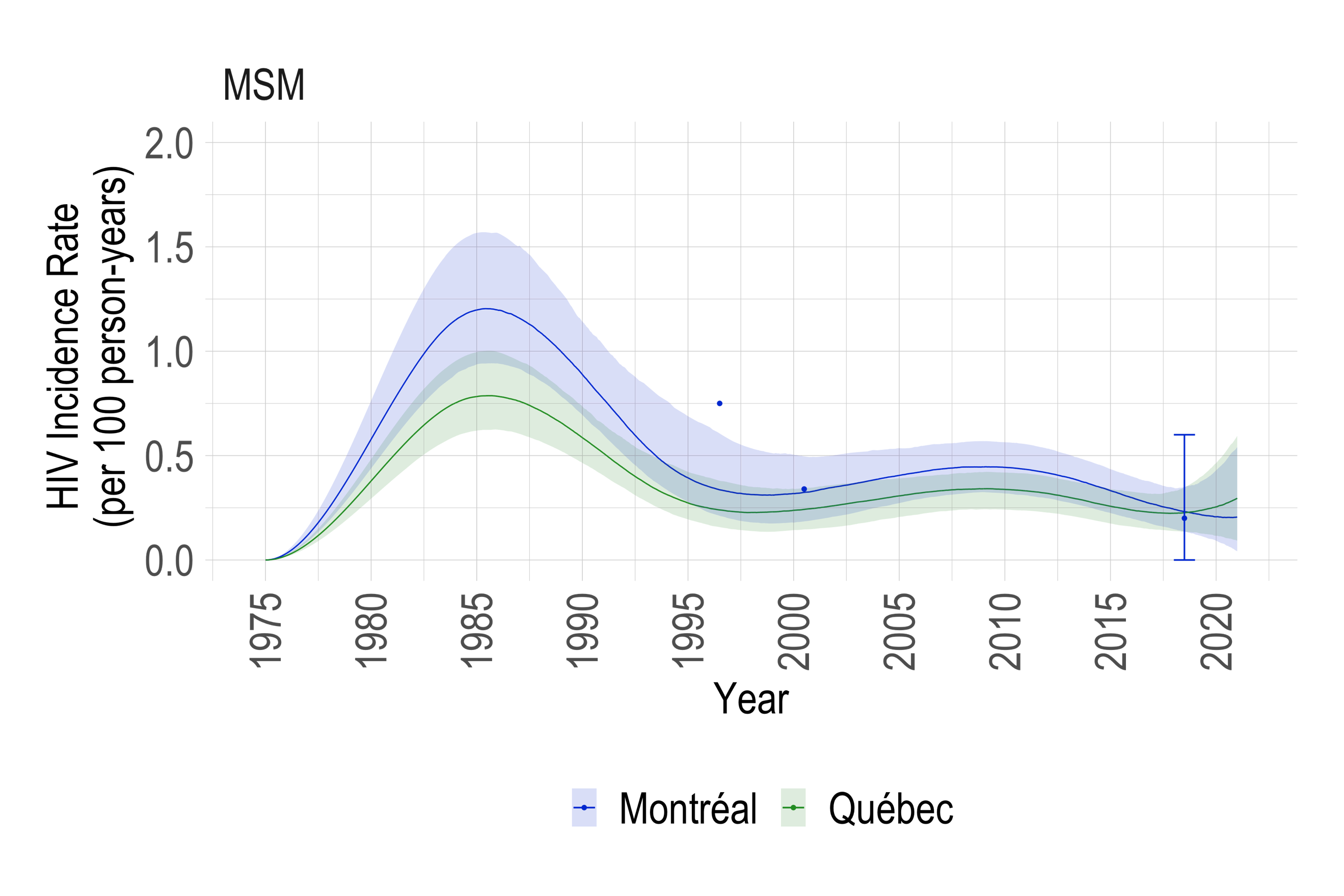 |
| --- |
| **Figure S16.** Estimated HIV incidence rate over 1975-2020 among men who have sex with men (MSM) in Montréal and the province of Québec. The coloured lines and bands display the posterior median and 95% credible intervals, respectively, with blue representing estimates from the Montréal region and green representing estimates for the province of Québec. The points and bars display the Omega study^41^ and Engage (Lambert G, personal communication, Dec. 2021) incidence rate estimates and corresponding 95% confidence intervals, respectively. |

## Additional figures among MSM including available cross-validation comparison points

| 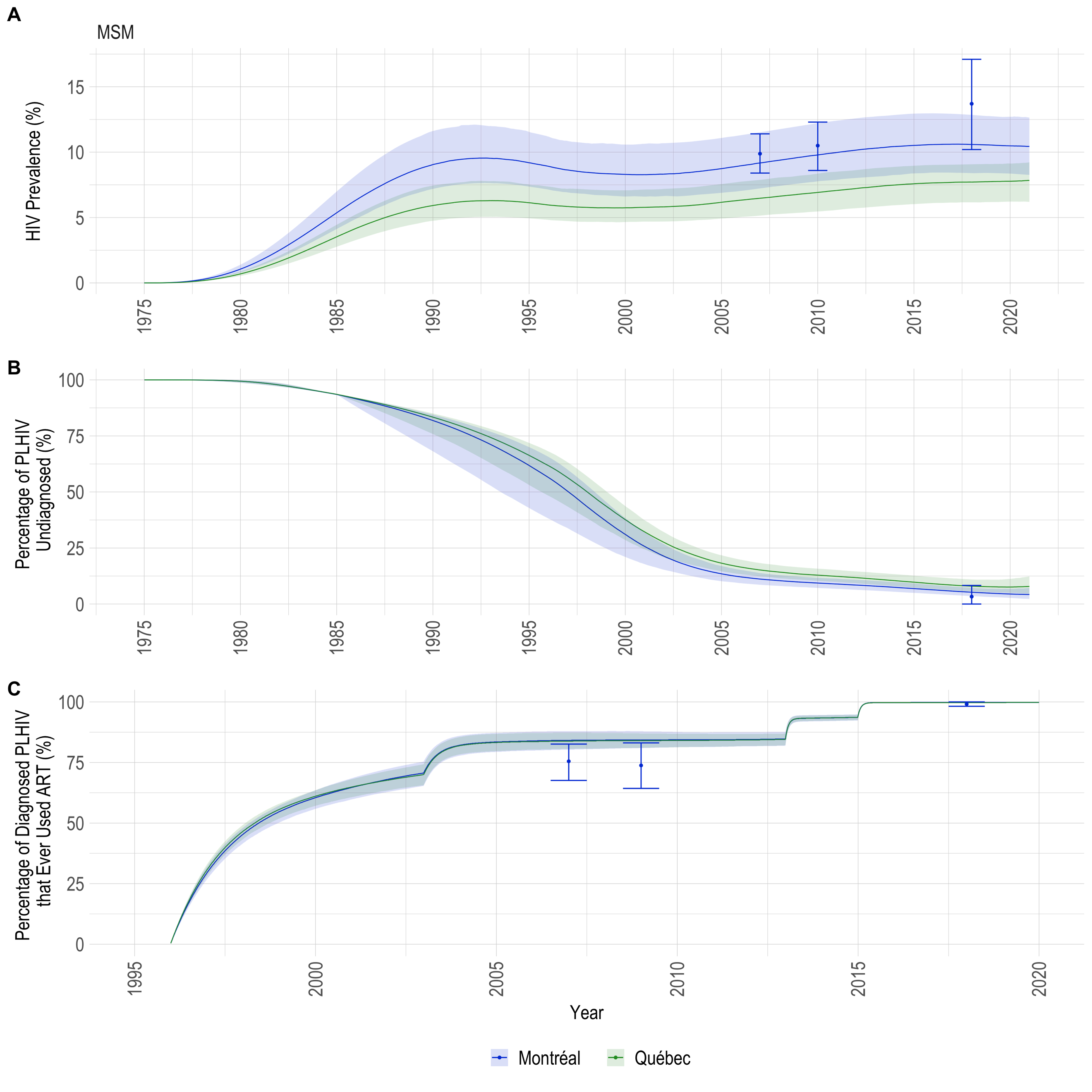 |
| --- |
| **Figure S17.** Estimated HIV prevalence (panel A), percentage of people living with HIV (PLHIV) undiagnosed (panel B), and percentage of diagnosed PLHIV that ever used antiretroviral treatment (ART; panel C) including cross-validation data over 1975-2020 among men who have sex with men (MSM) in Montréal and the province of Québec. The coloured lines and bands display the posterior median and 95% credible intervals, respectively, with blue representing estimates from the Montréal region and green representing estimates for all of Québec. The points and bars in 2007 and 2010 display the estimates from Argus and their corresponding 95% confidence intervals, respectively. The points and bars from 2018 display the Engage estimates and corresponding 95% confidence intervals, respectively. |

## AIDS-related mortality

| 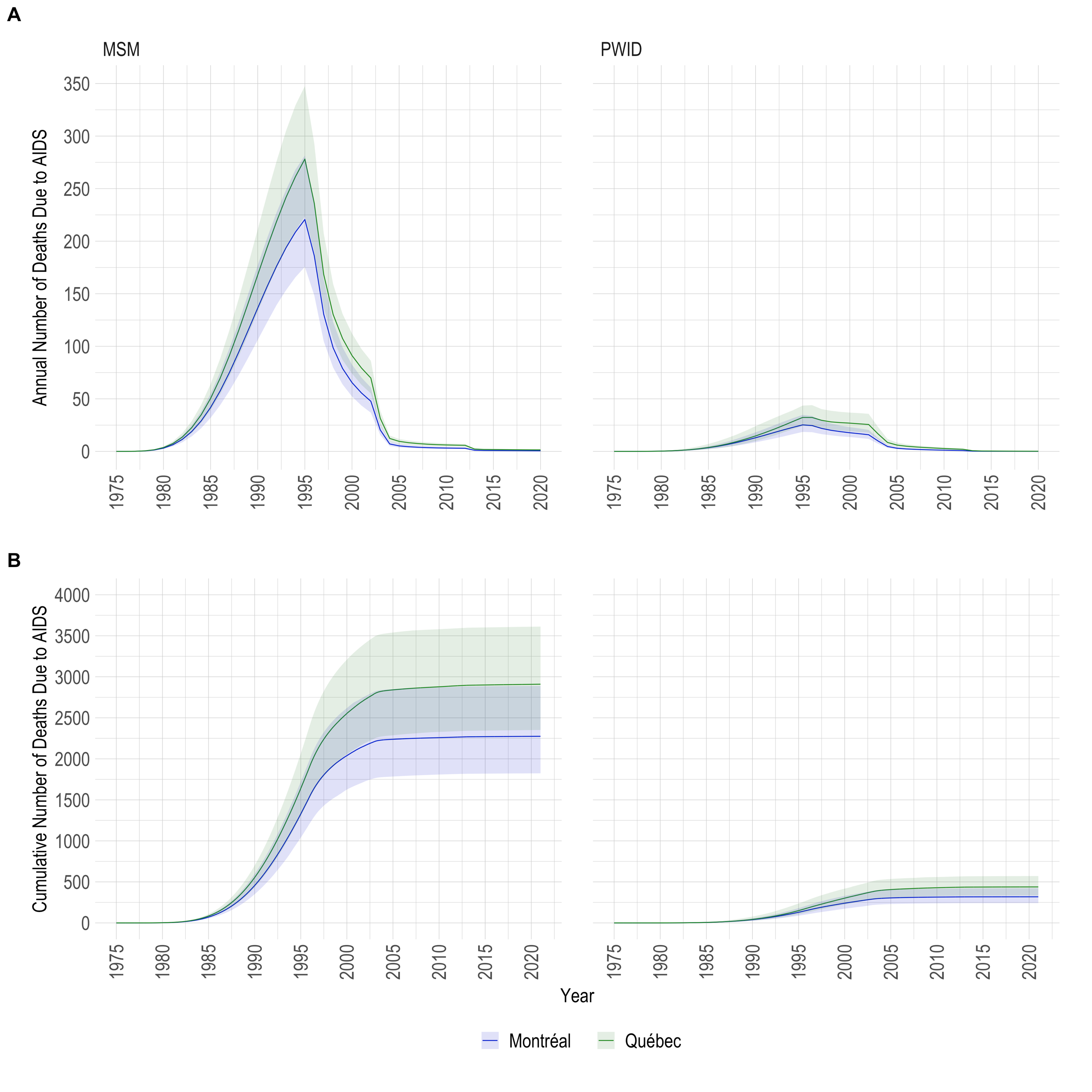 |
| --- |
| **Figure S18.** Estimated counts of AIDS-related mortality over 1975-2020 among men who have sex with men (MSM) and people who injected drugs (PWID) in Montréal and the province of Québec: A) the annual number of AIDS-related deaths; and B) the cumulative number of AIDS-related deaths. The coloured lines and bands display the posterior median and 95% credible intervals, respectively, with blue representing estimates from the Montréal region and green representing estimates for the province of Québec. |

# Additional results: sensitivity analyses

#### Including 2020 data in model calibration

## No reduction in testing rates over March 2020-year end

| 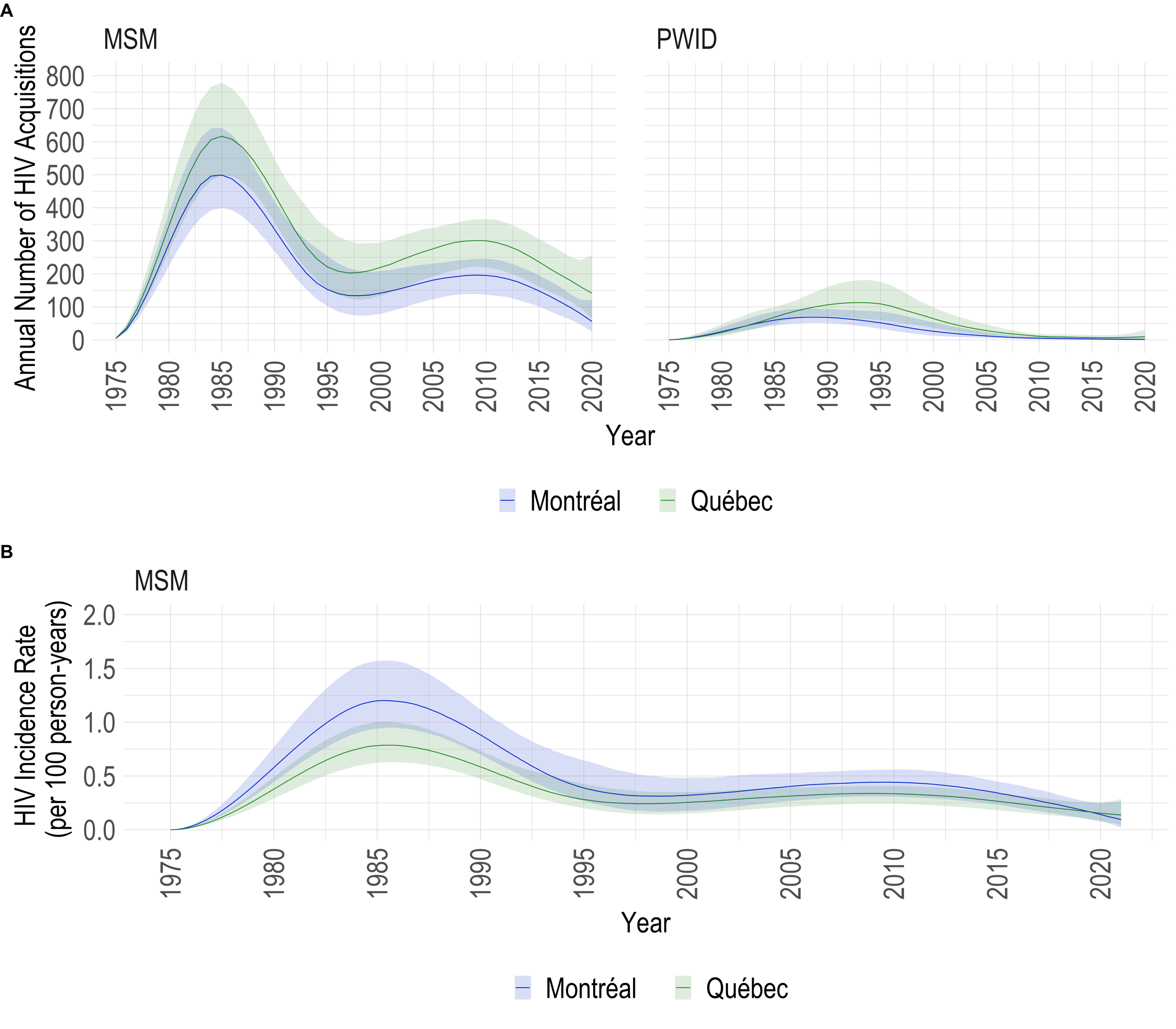 |
| --- |
| **Figure S19.** Estimated HIV incidence over 1975-2020 among men who have sex with men (MSM) and people who injected drugs (PWID) in Montréal and the province of Québec when 2020 data are excluded from model calibration and testing rates are not reduced during the COVID-19 pandemic: A) the annual number of HIV acquisitions among MSM and active PWID; and B) the HIV incidence rate among MSM. Incidence rates are not presented for PWID due to uncertainties in the denominator (the active PWID population size over time). The coloured lines and bands display the posterior median and 95% credible intervals, respectively, with blue representing estimates from the Montréal region and green representing estimates for the province of Québec. |

## 25% reduction in testing rates over March 2020-year-end

| 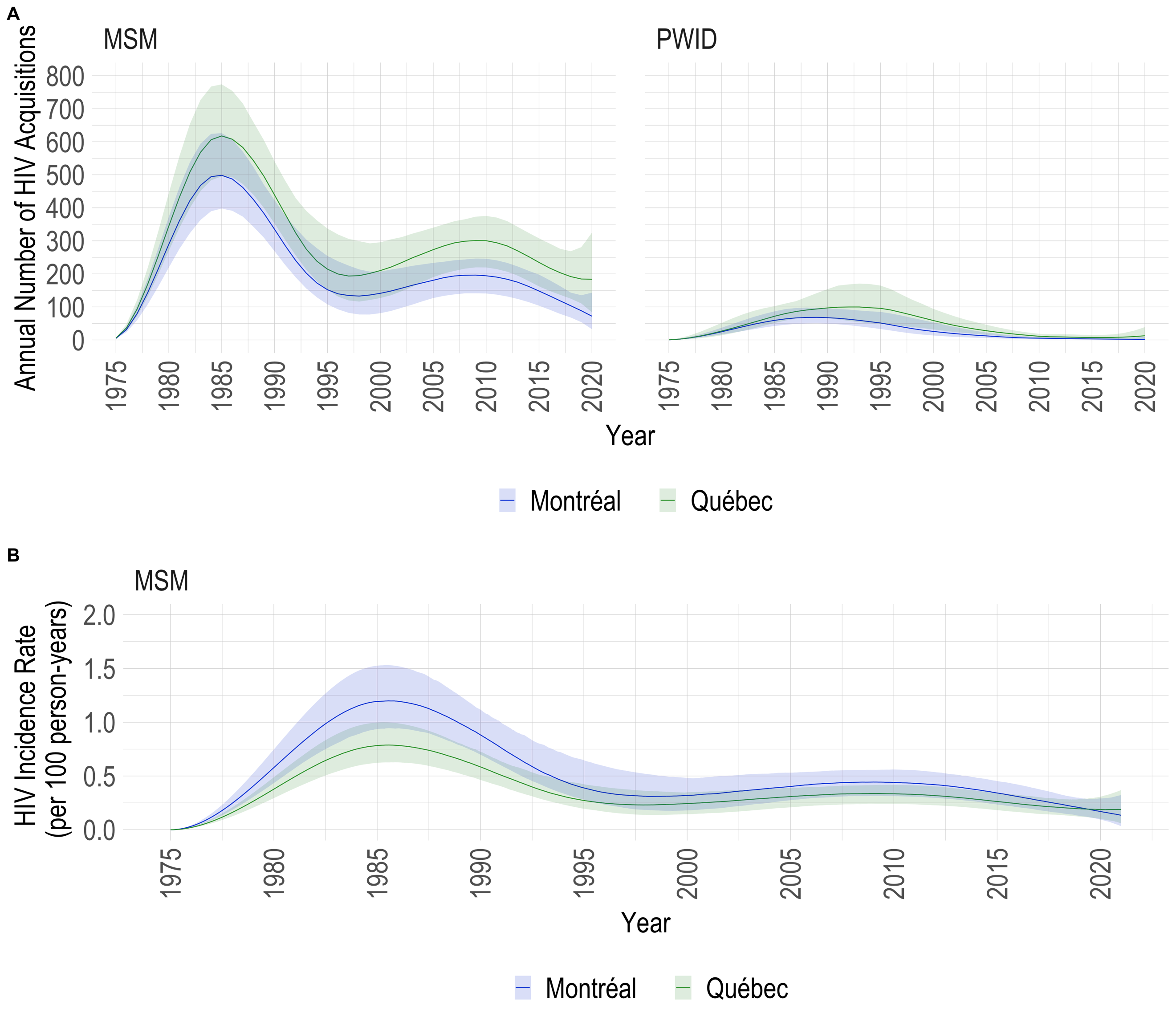 |
| --- |
| **Figure S20.** Estimated HIV incidence over 1975-2020 among men who have sex with men (MSM) and people who injected drugs (PWID) in Montréal and the province of Québec when 2020 data are excluded from model calibration and testing rates are reduced by 25% during the COVID-19 pandemic (March 2020-year-end): A) the annual number of HIV acquisitions among MSM and active PWID; and B) the HIV incidence rate among MSM. Incidence rates are not presented for PWID due to uncertainties in the denominator (the active PWID population size over time). The coloured lines and bands display the posterior median and 95% credible intervals, respectively, with blue representing estimates from the Montréal region and green representing estimates for the province of Québec. |

## 50% reduction in testing rates over March 2020-year-end

| 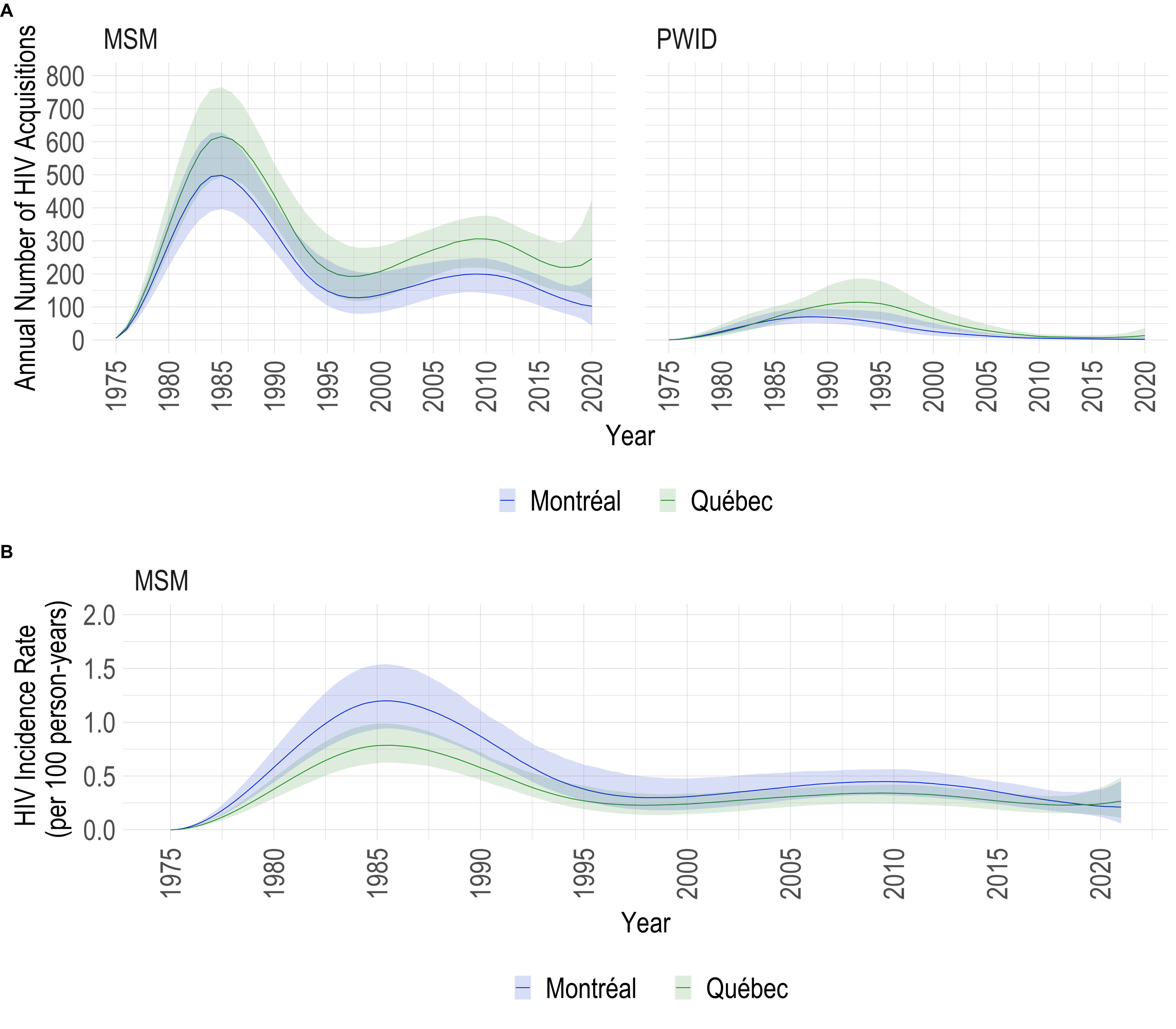 |
| --- |
| **Figure S21.** Estimated HIV incidence over 1975-2020 among men who have sex with men (MSM) and people who injected drugs (PWID) in Montréal and the province of Québec when 2020 data are excluded from model calibration and testing rates are reduced by 50% during the COVID-19 pandemic (March 2020-year-end): A) the annual number of HIV acquisitions among MSM and active PWID; and B) the HIV incidence rate among MSM. Incidence rates are not presented for PWID due to uncertainties in the denominator (the active PWID population size over time). The coloured lines and bands display the posterior median and 95% credible intervals, respectively, with blue representing estimates from the Montréal region and green representing estimates for the province of Québec. |

**Table S14:** Estimated HIV incidence, annual number of acquisitions, and percentage of people living with HIV undiagnosed in recent years (2017-2020) among men who have sex with men and people who inject drugs in Montréal and the province of Québec when 2020 data are included in model calibration and reductions in testing rates are explored^*^.

| **Location** | **Year** | **% reduction in testing rates from March 2020–year end** | **Incidence rate (95%CrI) per 100 PY at year end^†^** | **Annual number of HIV acquisitions (95%CrI)** | **% PLHIV undiagnosed (95%CrI) at year end** |
| --- | --- | --- | --- | --- | --- |
| *Men who have sex with men* | | | | | |
| Montréal | 2017 | 0% | 0.2 (0.1-0.3) | 116 (74-161) | 5.3 (3.6-7.4) |
|  | 2017 | 25% | 0.2 (0.2-0.3) | 118 (80-163) | 5.4 (3.8-7.5) |
|  | 2017 | 50% | 0.3 (0.2-0.4) | 127 (84-170) | 5.7 (4.1-7.8) |
|  | 2018 | 0% | 0.2 (0.1-0.3) | 98 (61-138) | 4.7 (3.1-6.6) |
|  | 2018 | 25% | 0.2 (0.1-0.3) | 104 (68-143) | 4.9 (3.4-6.8) |
|  | 2018 | 50% | 0.2 (0.1-0.4) | 116 (75-163) | 5.2 (3.6-7.2) |
|  | 2019 | 0% | 0.1 (0.1-0.2) | 78 (46-121) | 4.0 (2.6-5.9) |
|  | 2019 | 25% | 0.2 (0.1-0.3) | 89 (54-135) | 4.3 (2.9-6.0) |
|  | 2019 | 50% | 0.2 (0.1-0.4) | 107 (65-170) | 4.9 (3.2-6.9) |
|  | 2020 | 0% | 0.1 (0.0-0.3) | 56 (25-120) | 3.4 (2.1-5.3) |
|  | 2020 | 25% | 0.1 (0.0-0.3) | 72 (32-143) | 4.0 (2.6-6.1) |
|  | 2020 | 50% | 0.2 (0.1-0.5) | 102 (43-190) | 5.2 (3.2-7.8) |
| Province of Québec | 2017 | 0% | 0.2 (0.1-0.3) | 197 (131-271) | 7.7 (5.5-10.0) |
|  | 2017 | 25% | 0.2 (0.1-0.3) | 204 (137-276) | 7.9 (5.6-10.5) |
|  | 2017 | 50% | 0.2 (0.2-0.3) | 220 (150-294) | 8.3 (6.1-10.8) |
|  | 2018 | 0% | 0.2 (0.1-0.3) | 180 (116-250) | 7.0 (4.9-9.3) |
|  | 2018 | 25% | 0.2 (0.1-0.3) | 192 (125-269) | 7.4 (5.2-10.0) |
|  | 2018 | 50% | 0.2 (0.2-0.3) | 220 (148-305) | 7.9 (5.9-10.4) |
|  | 2019 | 0% | 0.2 (0.1-0.3) | 160 (95-242) | 6.4 (4.5-8.7) |
|  | 2019 | 25% | 0.2 (0.1-0.3) | 185 (110-281) | 6.9 (4.7-9.5) |
|  | 2019 | 50% | 0.2 (0.1-0.4) | 226 (141-346) | 7.7 (5.6-10.4) |
|  | 2020 | 0% | 0.1 (0.0-0.3) | 142 (64-257) | 5.9 (3.9-8.5) |
|  | 2020 | 25% | 0.2 (0.1-0.4) | 184 (84-325) | 7.0 (4.5-10.1) |
|  | 2020 | 50% | 0.3 (0.1-0.5) | 246 (124-423) | 8.6 (5.9-12.2) |
| *People who inject drugs (includes active and past injectors)* | | | | | |
| Montréal | 2017 | 0% | - | 2 (1-6) | 2.1 (0.8-5.8) |
|  | 2017 | 25% | - | 3 (1-6.5) | 2.2 (0.8-5.9) |
|  | 2017 | 50% | - | 2 (1-7) | 2.2 (0.7-6.1) |
|  | 2018 | 0% | - | 2 (1-6) | 1.8 (0.7-5.4) |
|  | 2018 | 25% | - | 2 (1-7) | 1.9 (0.7-5.7) |
|  | 2018 | 50% | - | 2 (1-7) | 1.9 (0.6-6.2) |
|  | 2019 | 0% | - | 2 (0-7) | 1.6 (0.5-5.2) |
|  | 2019 | 25% | - | 2 (0-7) | 1.7 (0.6-5.4) |
|  | 2019 | 50% | - | 2 (0-9) | 1.7 (0.5-6.0) |
|  | 2020 | 0% | - | 1 (0-9) | 1.4 (0.4-5.1) |
|  | 2020 | 25% | - | 2 (0-10) | 1.6 (0.5-5.6) |
|  | 2020 | 50% | - | 2 (0-10) | 1.7 (0.4-6.8) |
| Province of Québec | 2017 | 0% | - | 7 (3-15) | 3.3 (1.6-7.8) |
|  | 2017 | 25% | - | 8 (4-17) | 3.8 (1.9-9.1) |
|  | 2017 | 50% | - | 8 (4-17) | 3.4 (1.7-7.4) |
|  | 2018 | 0% | - | 8 (3-17) | 3.1 (1.5-7.5) |
|  | 2018 | 25% | - | 9 (3-21) | 3.7 (1.7-9.7) |
|  | 2018 | 50% | - | 9 (4-20) | 3.3 (1.6-7.7) |
|  | 2019 | 0% | - | 9 (3-22) | 3.1 (1.4-7.8) |
|  | 2019 | 25% | - | 10 (3-28) | 3.7 (1.6-10.3) |
|  | 2019 | 50% | - | 11 (4-27) | 3.3 (1.5-8.5) |
|  | 2020 | 0% | - | 10 (3-30) | 3.2 (1.3-8.3) |
|  | 2020 | 25% | - | 13 (3-39) | 4.0 (1.6-12.0) |
|  | 2020 | 50% | - | 13 (4-36) | 3.9 (1.6-10.2) |

Abbreviations: Credible interval (CrI); person-years (PY); people living with HIV (PLHIV).

^*^Using the same incidence knot attributes shown above in Table S13.

^†^Only presented for men who have sex with men, due to uncertainties in the denominator for people who inject drugs (active injectors).

# References

1. Mangal TD, Pascom ARP, Vesga JF, Meireles MV, Benzaken AS, Hallett TB. Estimating HIV incidence from surveillance data indicates a second wave of infections in Brazil. Epidemics. 2019;27:77-85.

2. van Sighem A, Nakagawa F, De Angelis D, Quinten C, Bezemer D, de Coul EO, et al. Estimating HIV Incidence, Time to Diagnosis, and the Undiagnosed HIV Epidemic Using Routine Surveillance Data. Epidemiology (Cambridge, Mass). 2015;26(5):653-60.

3. Statistics Canada. Table 17-10-0005-01 Population estimates on July 1st, by age and sex [Internet]. 2021 [cited 2022]. Available from: <https://www150.statcan.gc.ca/t1/tbl1/en/tv.action?pid=1710000501>.

4. Statistics Canada. 1976 Census of Canada. Population: Demographic Characteristics, Five-year Age Groups. In: Minister of Supply and Services Canada, editor.: Minister of Industry, Trade and Commerce; 1978.

5. Statistics Canada. 1981 Census of Population, Statistics Canada Catalogue no. 97-570-X1981004. 1982.

6. Statistics Canada. 1986 Census of Canada. Census Divisions and Subdivisions. Quebec: Part 1. Minister of Supply and Services Canada; 1987.

7. Statistics Canada. 1991 Census of Population, Statistics Canada Catalogue no. 95F0168X. 1992.

8. Estimations de la population des régions administratives selon le groupe d'âge et le sexe, âge médian et âge moyen, Québec, 1er juillet 1996 à 2021 [Internet]. 2022 [cited May 29, 2022]. Available from: <https://statistique.quebec.ca/en/document/population-and-age-and-sex-structure-administrative-regions/tableau/population-age-sex-median-administrative-regions-quebec#tri_tertr=06&tri_pop=10>.

9. Milwid RM, Xia Y, Doyle CM, Cox J, Lambert G, Thomas R, et al. Past dynamics of HIV transmission among men who have sex with men in Montréal, Canada: a mathematical modeling study. BMC infectious diseases. 2022;22(1):233.

10. Institut de la statistique du Québec. Enquête québécoise sur la santé de la population. 2014-2015.

11. Traoré I, Camirand H, Baulne J. Enquête québécoise sur la santé de la population, 2008 : analyse des données régionales. Recueil statistique, Québec. Gouvernement du Québec, Institut de la statistique du Québec; 2010.

12. Archibald CP, Jayaraman GC, Major C, Patrick DM, Houston SM, Sutherland D. Estimating the size of hard-to-reach populations: a novel method using HIV testing data compared to other methods. AIDS (London, England). 2001;15 Suppl 3:S41-8.

13. Adrien A, Dassa C, Leaune V, Perron M. Les Québécois face au sida: Attitudes envers les personnes vivant avec le VIH et gestions des risques. Direction de la santé publique, Régie régionale de la santé et des services sociaux de Montréal-Centre; 1998.

14. Public Health Agency of Canada. I-Track: Enhanced Surveillance of HIV, Hepatitis C and associated risk behaviours among people

who inject drugs in Canada. Phase 2 Report.: Centre for Communicable Diseases and Infection Control, Infectious Disease Prevention and Control Branch; 2013.

15. Remis R, Leclerc P, Routledge R, Taylor C, Bruneau J, Beauchemin J, et al. Consortium to characterize injection drug users in Canada (Montreal, Toronto and Vancouver). Final report: Toronto. 1998.

16. Leclerc P, Vandal AC, Fall A, Bruneau J, Roy É, Brissette S, et al. Estimating the size of the population of persons who inject drugs in the island of Montréal, Canada, using a six-source capture-recapture model. Drug Alcohol Depend. 2014;142:174-80.

17. Jacka B, Larney S, Degenhardt L, Janjua N, Høj S, Krajden M, et al. Prevalence of Injecting Drug Use and Coverage of Interventions to Prevent HIV and Hepatitis C Virus Infection Among People Who Inject Drugs in Canada. American journal of public health. 2020;110(1):45-50.

18. Statistics Canada. Table 17-10-0006-01 Estimates of deaths, by age and sex, annual [Internet]. 2021 [cited 2022]. Available from: <https://www150.statcan.gc.ca/t1/tbl1/en/tv.action?pid=1710000601>.

19. Mathers BM, Degenhardt L, Bucello C, Lemon J, Wiessing L, Hickman M. Mortality among people who inject drugs: a systematic review and meta-analysis. Bull World Health Organ. 2013;91(2):102-23.

20. Hollingsworth TD, Anderson RM, Fraser C. HIV-1 transmission, by stage of infection. The Journal of infectious diseases. 2008;198(5):687-93.

21. Cori A, Pickles M, van Sighem A, Gras L, Bezemer D, Reiss P, et al. CD4+ cell dynamics in untreated HIV-1 infection: overall rates, and effects of age, viral load, sex and calendar time. AIDS (London, England). 2015;29(18):2435-46.

22. Remis RS, Meunier L, Vandal AC, Palmer RWH, Desrochers D. AIDS Under-reporting may distort the epidemic: the Quebec experience [abstract no. Mo.C.205]. XI International Conference on AIDS; July 7-12; Vancouver, B.C.1996. p. 32.

23. Public Health Agency of Canada. M-Track: Enhanced Surveillance of HIV, Sexually Transmitted and Blood-borne Infections, and Associated Risk Behaviours among Men Who Have Sex with Men in Canada. Phase 1 Report.: Centre for Communicable Diseases and Infection Control, Infectious Disease Prevention and Control Branch, Public Health Agency of Canada; 2011.

24. Lambert G, Cox J, Tremblay F, Gadoury M-A, Frigault L, Tremblay C, et al. ARGUS 2005: Summary of the survey on HIV, viral hepatitis and sexually transmitted and blood-borne infections (STBI) as well as on the associated risk behaviours among Montreal men who have sex with men (MSM). Montreal Public Health Department, Institut national de santé publique du Québec and the Public Health Agency of Canada; 2006.

25. Lambert G, Cox J, Miangotar C, Tremblay C, Alary M, Otis J, et al. Argus 2008-2009: A survey on HIV, viral hepatitis and sexually transmitted infections (STI) as well as associated risk behaviours among Quebec men who have sex with men (MSM). Direction de santé publique se l’Agence de la santé services sociaux de Montréal, Institut national de santé publique du Québec and the Public Health Agency of Canada; 2011.

26. Lambert G, Cox J, Messier-Peet M, Apelian H, Moodie EEM, and the members of the Engage research team. Engage Montréal, Portrait of the sexual health of men who have sex with men in Greater Montréal, Cycle 2017-2018, Highlights. Direction régionale de santé publique, CIUSSS du Centre-Sud-de-l’Île-de-Montréal; 2019 January.

27. Hankins C, Alary M, Parent R, Blanchette C, Claessens C, Group TSW. Continuing HIV Transmission Among Injection Drug Users in Eastern Central Canada: The SurvUDI Study, 1995 to 2000. JAIDS Journal of Acquired Immune Deficiency Syndromes. 2002;30(5):514-21.

28. Leclerc P, Roy É, Morissette C, Alary M, Blouin K. Surveillance des maladies infectieuses chez les utilisateurs de drogue par injection: épidémiologie du VIH de 1995 à 2018 et épidémiologie du VHC de 2003 à 2018. Technical Report. Gouvernement du Québec, Institut national de santé publique du Québec; 2021.

29. Ramsay JO. Monotone regression splines in action. Statistical science. 1988:425-41.

30. Wang W, Yan J. splines2: Regression Spline Functions and Classes. R package version 0.4.4 ed2021.

31. Canadian HIV/AIDS Legal Network. HIV Testing. Info Sheet. 2007 April 26.

32. CATIE. New PHAC testing guide includes recommendations to promote HIV testing during routine medical care 2013 [updated July 13, 2013; cited 2022. Available from: <https://www.catie.ca/prevention-in-focus/new-phac-testing-guide-includes-recommendations-to-promote-hiv-testing-during>.

33. Vynnycky E, White R. An introduction to infectious disease modelling: Oxford University Press; 2010.

34. Menzies NA, Soeteman DI, Pandya A, Kim JJ. Bayesian Methods for Calibrating Health Policy Models: A Tutorial. PharmacoEconomics. 2017;35(6):613-24.

35. Surveillance des cas de syndrome d'immunodéficience acquise (SIDA) cas cumulatifs 1979-2003. Programme de surveillance du sida du Québec, Direction générale de la santé publique, Ministère de la Santé et des Services sociaux.

36. Surveillance des cas de syndrome d'immunodéficience acquise (SIDA) cas cumulatifs 1979-2003: Montréal-Centre (Région 06). Programme de surveillance du sida du Québec, Direction générale de la santé publique, Ministère de la Santé et des Services sociaux.

37. Institut national de santé publique du Québec. Programme de surveillance de l’infection par le virus de l’immunodéficience humaine (VIH) au Québec: Rapport Annuel 2016. Gouvernement du Québec; 2017.

38. Moore DM, Cui Z, Skakoon-Sparling S, Sang J, Barath J, Wang L, et al. Characteristics of the HIV cascade of care and unsuppressed viral load among gay, bisexual and other men who have sex with men living with HIV across Canada’s three largest cities. Journal of the International AIDS Society. 2021;24(4):e25699.

39. Bassett R, Deride J. Maximum a posteriori estimators as a limit of Bayes estimators. Mathematical Programming : A Publication of the Mathematical Optimization Society. 2019;174(1-2):129-44.

40. Gelman A, Carlin JB, Stern HS, Dunson DB, Vehtari A, Rubin DB. Bayesian Data Analysis. 3rd ed: Chapman and Hall/CRC; 2013 Nov. 1. 675 p.

41. Remis RS, Alary M, Otis J, Masse B, Demers E, Vincelette J, et al. No increase in HIV incidence observed in a cohort of men who have sex with other men in Montreal. AIDS (London, England). 2002;16(8):1183-5.
